# Unmetabolized Folic Acid: Biology, Epidemiology, and Clinical Consequences: A Systematic Review

**DOI:** 10.3390/nu18172887

**Published:** 2026-09-03

**Authors:** Richard E. Frye, Daniel A. Rossignol

**Affiliations:** 1 Autism Discovery and Treatment Foundation, Phoenix, AZ 85050, USA; rossignolmd@gmail.com; 2 Rossignol Medical Center, Phoenix, AZ 85050, USA; 3 Rossignol Medical Center, Aliso Viejo, CA 92656, USA

**Keywords:** unmetabolized folic acid, folic acid, 5-methyltetrahydrofolate, folate fortification, dihydrofolate reductase, one-carbon metabolism, folate receptor alpha, systematic review, meta-analysis

## Abstract

**Background:** Mandatory folic acid (FA) fortification prevents neural tube defects but generates unmetabolized folic acid (UMFA) in blood, because hepatic dihydrofolate reductase (DHFR) reduces FA only slowly. Whether UMFA itself, as distinct from high total folate, carries biological consequences is unresolved. **Methods:** We systematically searched six databases (January 1995–May 2026; PROSPERO CRD420261407616) and included 65 publications describing 61 unique primary studies, yielding 71 analytic contributions (15 mechanistic, 24 observational, 32 interventional). The question of whether equimolar (6S)-5-MTHF substitution alters UMFA, erythrocyte folate, or total homocysteine (tHcy) versus FA was evaluated using random-effects meta-analysis with a DerSimonian–Laird estimator for τ^2^ and Hartung–Knapp–Sidik–Jonkman small-sample confidence intervals and was rated with GRADE. Mechanistic and observational streams were synthesized narratively and classified by exposure metric (UMFA, total folate, FA intake, fortification). **Results:** 5-MTHF substitution reduced plasma UMFA (SMD +0.99; 95% CI +0.20 to +1.79; *I*^2^ = 30%; *k* = 3; GRADE low). The two trials reporting erythrocyte folate were directionally discordant (Hedges’ *g* +0.40; +0.08 to +0.73 and −0.38; −0.92 to +0.16; *I*^2^ = 83%) and were not pooled. For tHcy, no pooled estimate could be derived; it is reported qualitatively. Observational UMFA–outcome associations were inconsistent, confounded by total folate, and unreplicated. **Conclusions:** Substitution of (6S)-5-MTHF for FA appears to reduce circulating UMFA. For erythrocyte folate, the two available trials were directionally discordant but insufficient to establish equivalence or non-inferiority. For tHcy, no between-form difference was detected. With two to three trials per outcome, no prespecified margins, and no trial designed for that purpose, the evidence cannot support an equivalence or non-inferiority claim for either outcome and clinical benefit remains unestablished. Current evidence does not establish that UMFA independently causes harm.

## 1. Introduction

### 1.1. One-Carbon Metabolism and the Centrality of Folate

Folate (vitamin B_9_) sits at the center of one-carbon metabolism, the network through which methyl groups are generated, donated, and recycled to support nucleotide biosynthesis, amino acid interconversion, mitochondrial translation, and DNA and histone methylation. The reduced cofactor pool comprises tetrahydrofolate (THF), 5,10-methylenetetrahydrofolate, 5-methyltetrahydrofolate (5-MTHF), and 10-formyltetrahydrofolate, which traffic one-carbon units between purine and thymidylate synthesis, homocysteine remethylation, and generation of S-adenosylmethionine (SAM) [1,2,3,4,5]. Disturbance of this network is implicated in megaloblastic anemia, neural tube defects (NTDs), vascular disease, cancer, and neurocognitive disorders [6].

Natural dietary folates are predominantly polyglutamylated reduced forms, with 5-MTHF the major circulating species [7]. Folic acid (FA; pteroylmonoglutamic acid), the form used in fortification, supplements, and pharmacotherapy, is a fully oxidized synthetic pteridine that does not occur in nature in measurable amounts and must be reduced sequentially to dihydrofolate (DHF) and then THF. Both steps are catalyzed by dihydrofolate reductase (DHFR; EC 1.5.1.3), encoded on chromosome 5q11.2–q13.3 [8].

### 1.2. Mandatory Folic Acid Fortification: A Global Public-Health Intervention

The MRC Vitamin Study randomized 1817 women at high recurrence risk and showed that 4 mg/day periconceptional FA reduced recurrent NTDs by 72% (RR 0.28, 95% CI 0.12–0.71) [9]. The United States introduced mandatory FA fortification of enriched cereal grain products in 1998 at 140 µg per 100 g of flour, and more than 80 countries have since followed. US spina bifida and anencephaly prevalence fell by approximately 28% within five years [10]. Fortification is among the most successful public-health nutrition interventions on record, and nothing in the present synthesis questions its continuation.

Fortification also exposed the whole population, not solely women of reproductive age, to chronic low-dose synthetic FA. Combined with discretionary supplementation, many adults now ingest 600–1000 µg of FA daily [11,12]. Nationally representative United States intake data indicate that fortification has not driven usual FA intake in women of reproductive age above the Institute of Medicine Tolerable Upper Intake Level of 1000 µg/day, the median all-source intake being 661 µg/day [13]; in the Canadian MIREC pregnancy cohort, by contrast, about 25% of participants exceeded 1000 µg/day [14].

### 1.3. The Emergence of Unmetabolized Folic Acid

That FA is not fully reduced after oral intake was first shown in 1997, when single doses above ~200 µg produced a detectable bolus of intact pteroylmonoglutamate in serum within 90 min, termed unmetabolized folic acid (UMFA) [15]. Fortification-level intakes were subsequently shown to generate post-prandial UMFA peaks in healthy adults [16,17], and modern NHANES LC-MS/MS surveillance detected UMFA in >95% of serum samples, although the prevalence above 1 nmol/L was substantially lower and depends on fasting status [18].

The mechanism is a low-capacity reductive step. Human hepatic DHFR is about 1.4% as active per milligram of protein as rat hepatic DHFR, with a roughly five-fold inter-individual range [19]. Portal-vein sampling further showed the human intestinal mucosa to be functionally inactive for FA reduction: approximately 80% of an oral 220 µg FA dose entered the portal vein as intact FA, whereas an equimolar dose of the already-reduced 5-formyl-THF appeared in portal blood almost entirely as reduced folate, principally 5-MTHF [20]. The system therefore appears readily saturable under common fortification-plus-supplement exposures.

### 1.4. Established and Unestablished Actions of Folic Acid

Two molecular interactions of intact FA in human physiology are well characterized and are the ones invoked by proposed UMFA-specific mechanisms: it is a substrate for DHFR [8,19], and in its intact oxidized form it binds the soluble folate-binding protein (FBP) and membrane folate receptors, principally folate receptor-α (FRα), competing with reduced folates for receptor occupancy [21,22]. No additional mechanism specific to intact FA has been established as relevant to the proposed UMFA effects, and no direct human study demonstrates clinical harm from either interaction: DHFR saturation is a pharmacokinetic observation rather than a toxicity, and FRα competition has been shown in vitro [22,23] and in non-human primates [21] but not linked to a clinical deficit. No study has shown that FA exposure lowers cerebrospinal fluid (CSF) folate in humans (Section 4.2).

### 1.5. Why UMFA Is Nevertheless Worth Measuring

Unmetabolized folic acid is nevertheless a biologically distinguishable analyte that is dose-dependent, individually variable, and modifiable by folate form, and several testable mechanisms have been proposed. Unmetabolized folic acid binds FBP and FRα and may competitively inhibit reduced-folate uptake at these high-affinity transporters [22], including at the choroid plexus [21]; FBP-bound UMFA inhibits natural killer (NK) cell cytotoxicity [23,24,25]; and in carriers of the methylenetetrahydrofolate reductase (MTHFR) 677C→T polymorphism, FA intake elevates S-adenosylhomocysteine and disturbs the SAM:SAH ratio [26]. Rodent studies of high-dose FA report altered offspring behavior [27], adverse embryonic outcomes [28], and embryonic delay and growth retardation on a 20-fold supplemented diet [29]; these exposures are supraphysiologic and are mechanism-generating rather than evidence of human risk. Observational cohorts have associated high circulating folate, particularly with low vitamin B_12_, with cognitive impairment and anemia in older adults [30], colorectal cancer recurrence [31], and child neurodevelopmental outcomes [32,33]—a literature that is, as set out in Section 3.6 and Section 4.4, predominantly null or protective.

### 1.6. The FRα–Cerebral Folate Deficiency Axis

Folate Receptor α autoantibodies (FRAAs) are detected in a large proportion of children with autism spectrum disorder (ASD) in clinic-based cohorts—75.3% of 93 children in one series [34] and 76% of 82 children in another [35], with a pooled estimate of 71% (95% CI 64–77%) [36]. Seropositivity is not, however, equivalent to cerebral folate deficiency (CFD): the same autoantibodies occur in 75% of unaffected siblings and 29% of unrelated controls [35], cerebrospinal fluid 5-MTHF was measured in only 16 of the 93 children in the largest ASD series, where values fell below the normative mean (82 ± 31 nmol/L; normal range 44–181 nmol/L) rather than below the normal range [34], and the pooled prevalence of documented CFD in ASD is 38% (95% CI 11–71%; *I*^2^ = 92%), falling to 32% (19–45%) after removal of an outlying study [36]. Titers also fluctuate over time [37], and CFD arises independently of autoantibodies through FOLR1 variants and mitochondrial disease [38], so seropositivity is neither necessary nor sufficient for a CSF folate deficit. Where a CSF deficit is documented, leucovorin (calcium d,l-5-formyl-THF), which bypasses FRα, has been reported to benefit at least one speech, autism-score, or social-reciprocity outcome in one placebo-controlled trial [39], two further placebo-controlled trials [40,41], and one assessor-blinded trial [42]. This literature is contextual only: efficacy in established ASD is not the registered subject of this review, and the authors’ competing interests in this area are declared below.

### 1.7. Objectives

The specific gap this review addresses is narrow and, so far as the authors are aware, has not previously been synthesized: although FA and (6S)-5-MTHF have each been studied extensively against placebo, the head-to-head randomized comparison of the two folate forms at matched dose—the only design that separates folate form from folate dose—has never been pooled. That comparison is the primary contribution of this review; everything else it reports is context for interpreting it.

Accordingly, this review is organized around three explicitly distinct levels of evidence, which are labeled as such in the Results and Discussion and are not interchangeable:

1. Registered evidence. One prespecified PICO question, to which structured eligibility, quantitative synthesis, and GRADE certainty rating were applied: in humans receiving folate supplementation, does substitution of equimolar (6S)-5-MTHF for synthetic FA alter plasma UMFA, erythrocyte folate, or plasma tHcy? This is the only stream from which a certainty-rated answer is drawn.
2. Secondary quantitative evidence. Exploratory pools (S1, S3, S4) that were not part of the registered question are not GRADE-rated and are hypothesis-generating only.
3. Contextual evidence. Mechanistic, observational, and trial-level pharmacology synthesized narratively, addressing (i) the pharmacokinetic basis for UMFA generation, (ii) observational associations between folate exposure metrics and health outcomes and how those metrics are distinguished from one another, and (iii) the dose dependence of UMFA generation. No certainty rating is assigned to this stream, and no conclusion of this review rests on it alone.

## 2. Materials and Methods

### 2.1. Protocol and Registration

The protocol was registered a priori with PROSPERO (CRD420261407616), and reporting follows the PRISMA 2020 statement [43]; the completed checklist is provided as Supplementary Table S9. Formal GRADE ratings, structured PICO eligibility, and prespecified outcome definitions were applied to the registered question only, since GRADE addresses certainty about a specific question; aggregate GRADE ratings are therefore not assigned across heterogeneous contextual evidence.

### 2.2. Eligibility Criteria

Eligible studies enrolled humans of any age, mammalian cells or tissues, or animal models of mammalian folate metabolism, and reported on synthetic FA (from fortified food or supplements), (6S)-5-MTHF, L-methylfolate, or leucovorin. The primary outcome was detection or concentration of UMFA in any biological compartment, or any biological or clinical endpoint plausibly attributable to folate form or dose. Studies of plant or microbial folate metabolism, pediatric leukemia chemotherapy unrelated to UMFA, editorials, single-case reports lacking mechanistic content, retracted papers, unpublished conference abstracts, and superseded duplicate cohort reports were excluded. Reviews, meta-analyses, and other secondary syntheses were also ineligible as included evidence: they were used for citation chasing and contextual orientation only, are cited in the narrative where relevant, and are not counted as included reports, studies, or analytic contributions. Searches covered January 1995 to May 2026 and were restricted to English-language peer-reviewed publications.

For the registered PICO question, eligibility was narrower: Population—humans of any age receiving oral folate supplementation; Intervention—(6S)-5-MTHF or L-methylfolate; Comparator—synthetic FA at an equimolar dose in the same trial; Outcomes—plasma or serum UMFA, erythrocyte folate, plasma tHcy; Design—randomized or randomized crossover trial.

### 2.3. Information Sources and Search Strategy

PubMed/MEDLINE, Embase, Cochrane CENTRAL, Scopus, Web of Science, and Google Scholar were searched in May 2026. The full PubMed search string is reproduced in Supplementary Methods. Citation chasing of three pivotal references [15,39,44], together with hand-search of ClinicalTrials.gov and the bibliographies of relevant US Food and Drug Administration briefing books, yielded additional records.

### 2.4. Selection Process and Data Extraction

Two reviewers independently screened titles, abstracts, and full texts, with discordances resolved by consensus and, when required, by adjudication from a third reviewer. Data were extracted into structured spreadsheets capturing study population, design, intervention, dose, duration, UMFA assay methodology, effect estimates with 95% confidence intervals, and risk-of-bias domains.

**Figure 1:**
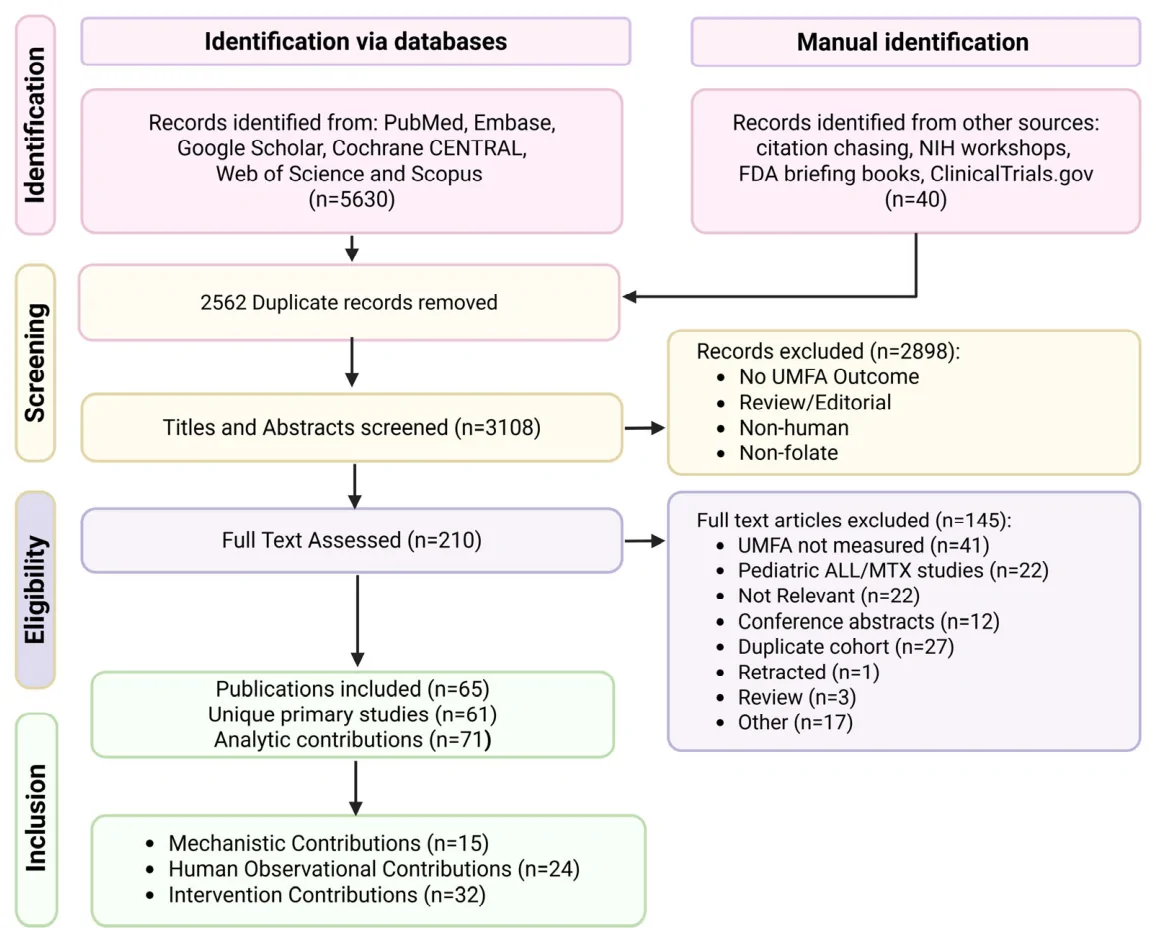
PRISMA 2020 flow diagram. Counts follow the unit-of-analysis rules stated in Section 2.4: 65 unique publications describing 61 unique primary studies met the inclusion criteria and generated 71 analytic contributions (one study-by-outcome entry each) across the evidence tables, so the three stream counts sum to contributions rather than to studies. This figure is original and was newly generated by the authors for this revision and is not reproduced or adapted from any published figure.

Three counts are distinguished throughout. A report is a single publication. A study is a single investigation, which may be described in more than one report. An analytic contribution is one study-by-outcome entry in the evidence tables. The evidence base comprises 65 unique publications describing 61 unique primary studies, which generate the 71 analytic contributions tabulated in Supplementary Tables S1–S3; the difference arises because several investigations report mechanistic and human data, or more than one eligible outcome, in separate places. Where two or more reports described the same cohort or the same randomized participants, the index report was designated the unit of analysis and the companion reports were used only for supplementary outcome values, never as independent contributions to a pool. Reviews, meta-analyses, and other secondary syntheses were used for citation chasing and contextual orientation only; they are cited in the narrative where relevant but are not counted as included reports, studies, or analytic contributions, and they contribute to no pool. The counts shown in Figure 1 are report counts during screening and explicitly distinguish publication, primary study, and analytic contribution counts at inclusion; Supplementary Tables S1–S3 identify the study underlying each analytic contribution.

### 2.5. Exposure Classification of Observational Studies

The observational evidence base is not homogeneous with respect to the exposure actually measured, and pooling or narratively co-presenting studies of directly measured UMFA with studies of total folate, of FA intake, or of population fortification risks attributing to UMFA effects that belong to folate exposure generally. The a priori inclusion rationale is therefore stated explicitly, and every observational study is classified by exposure metric.

The registered protocol admitted observational studies reporting “any biological or clinical endpoint plausibly attributable to UMFA.” Because UMFA is generated by, and is monotonically related to, FA intake [14,15,16,45], studies of FA intake and of fortification were admitted as upstream proxies for UMFA exposure, and studies of total circulating folate were admitted as composite measures containing a UMFA component. This is a defensible sampling frame for a contextual literature, but it is not a valid basis for causal attribution to UMFA. Observational evidence is accordingly stratified into four mutually exclusive exposure classes (Table 1), and conclusions about UMFA specifically are drawn only from Class 1.

### 2.6. Risk of Bias Assessment

Appraisal was tailored to design: Cochrane Risk of Bias 2.0 for randomized controlled trials [69], ROBINS-I for non-randomized human studies [70], and SYRCLE for animal experiments [71]. Each study was appraised independently by two reviewers, with disagreements resolved by consensus and, where consensus was not reached, by adjudication from a third reviewer; no automation tools were used. The tool applied to each contribution is stated in Supplementary Tables S1–S3, and the design-tailored rule is applied without exception: non-randomized human interventions, including single-arm and case-series designs, are appraised with ROBINS-I and not with RoB 2.0, and reviews and other secondary syntheses receive no trial risk-of-bias judgment because they are not included in the evidence. For randomized trials in which the primary analysis was per protocol rather than intention-to-treat, the deviations-from-intended-interventions and missing-outcome-data domains are judged against the effect of assignment to intervention, and the resulting overall judgment is reported per outcome where the analyzed denominators differ across outcomes within a trial. GRADE [72] was applied only to the registered PICO question. The Oxford Centre for Evidence-Based Medicine 2011 Levels of Evidence [44] were additionally applied descriptively to human studies.

### 2.7. Assay Generation and Fasting Status

Two methodological considerations particular to this literature deserve emphasis. First, assay generation affects interpretability: the affinity-HPLC method underlying much pre-2010 work co-elutes the catabolite MeFox with FA and overestimates true UMFA 2–3-fold [73], whereas LC-MS/MS has been the operative gold standard since 2010 and underpins modern NHANES surveillance [74,75]; the foundational Troen [23] and Morris [30,49] findings were therefore interpreted with quantitative awareness of this inflation. Second, non-fasting samples show higher odds of UMFA > 1 nmol/L than fasting serum samples [18], so studies were tagged by fasting status and pooled analyses restricted to fasting samples where possible. A single UMFA measurement also conflates dose with time since last ingestion [76].

### 2.8. Data Synthesis

Where at least three independent studies were sufficiently comparable in population, exposure, comparator, and outcome metric, quantitative synthesis was performed using random-effects meta-analysis with a DerSimonian–Laird estimator for τ^2^ and Hartung–Knapp–Sidik–Jonkman small-sample confidence intervals [77,78,79,80]. Hedges’ g standardized mean differences (SMD, with small-sample correction J) were used for continuous biomarkers reported on heterogeneous assays (plasma UMFA, plasma 5-MTHF, erythrocyte folate) and raw mean differences (MD, µmol/L) for plasma tHcy [81].

Heterogeneity was quantified by τ^2^ and *I*^2^. A single prediction-interval rule was applied throughout: because a prediction interval requires τ^2^ to be estimated with usable precision, prediction intervals were computed only for pools with *k* ≥ 5; for smaller pools the interval is uninterpretable and is neither reported nor used in any inference. No pool in this review reached *k* = 5, so no prediction interval is reported anywhere, and no statement in this review rests on one. A pooled estimate was withheld altogether under two conditions: where only two studies contributed and they were directionally discordant, since a two-study HKSJ interval is not informative; and where none of the contributing reports supplied the per-arm arithmetic means and standard deviations that the specified effect metric requires, since any pooled value would then rest on assumptions about distributional form and variance that the sources do not support.

Medians with interquartile range or with full range were converted using Methods 3 and 1 of Wan et al. [82], applying the sample-size-dependent η(*n*) denominator rather than the asymptotic constant IQR/1.35 (Supplementary Methods Section S1), SDs were recovered from standard errors as SE × √*n* [83], and UMFA concentrations were converted from ng/mL to nmol/L using the FA molecular weight 441.4 g/mol. Where a report gave the total randomized or analyzed sample without an arm-level breakdown at the outcome time point, per-arm denominators were derived from internal evidence in the source publication wherever that evidence determines them uniquely, and no equal-allocation assumption is used anywhere in the final analysis.

One denominator is not uniquely determined by any reported quantity and is therefore carried as an explicit assumption rather than a derivation: the arm-specific split of the 124 quantifiable plasma-UMFA observations in Troesch 2019 [84]. The derivation procedure and the resulting denominators for the trials contributing to Pools P1 and P2 are set out in Supplementary Methods and in the footnotes to Supplementary Tables S4 and S5. Where per-arm variances were reported only as geometric means with confidence intervals, or were not recoverable from the published report, no effect estimate was constructed, and the finding is reported qualitatively.

When publications reported overlapping outcomes from the same cohort, only the index publication was retained; a 2024 FACT-cohort metabolomics publication [85] overlapping with Murphy et al. [45] was removed. Source-anchored extraction showed that the Wan range estimator applied to Tam et al. [86] was dominated by a single 86.4 nmol/L low-dose outlier and that the primary publication reports no significant between-dose UMFA difference, so an exploratory post-hoc analysis excluding that trial was performed (Supplementary Figure S3b).

Prespecified subgroups were population, exposure dose, assay generation, trial duration, and risk-of-bias domain; prespecified sensitivity analyses were restriction to LC-MS/MS-assayed trials, leave-one-out resampling, and addition of one matched trial reporting an alternative compartment [87]. Funnel plots were not produced because no pool contained a sufficient number of studies for a funnel plot to be interpretable; Egger’s test was prespecified for pools with ≥10 studies and, as no pool reached that threshold, was not performed [88]. Analyses used Python 3.11 (numpy 1.26, scipy.stats 1.12) with procedures algorithmically equivalent to the R *meta* and *metafor* packages [89].

### 2.9. Certainty of Evidence

GRADE [72] was applied to the registered PICO question, with up- or down-grading for risk of bias, inconsistency, indirectness, imprecision, publication bias, dose-response gradient, magnitude of effect, and plausible residual confounding. Contextual mechanistic and observational evidence is appraised descriptively without aggregate GRADE labels.

## 3. Results

### 3.1. Study Selection

Of 5670 records identified (5630 from six databases and 40 from ancillary sources), 3108 unique records were screened after deduplication, and after full-text assessment of 210 reports, of which 145 were excluded, 65 unique publications describing 61 unique primary studies met inclusion criteria, yielding 71 analytic contributions (15 mechanistic, 24 observational, 32 interventional) across the evidence tables (Figure 1; counting rules in Section 2.4); study-level characteristics are given in Supplementary Tables S1–S3. All 51 studies contributing human participant data were descriptively appraised for risk of bias and design quality, with formal GRADE applied to the registered question and OCEBM levels assigned descriptively; pre-2010 affinity-HPLC studies are flagged for assay-generation effects.

### 3.2. Primary Quantitative Synthesis: Folic Acid Versus (6S)-5-MTHF (Pools P1–P3)

**Figure 2:**
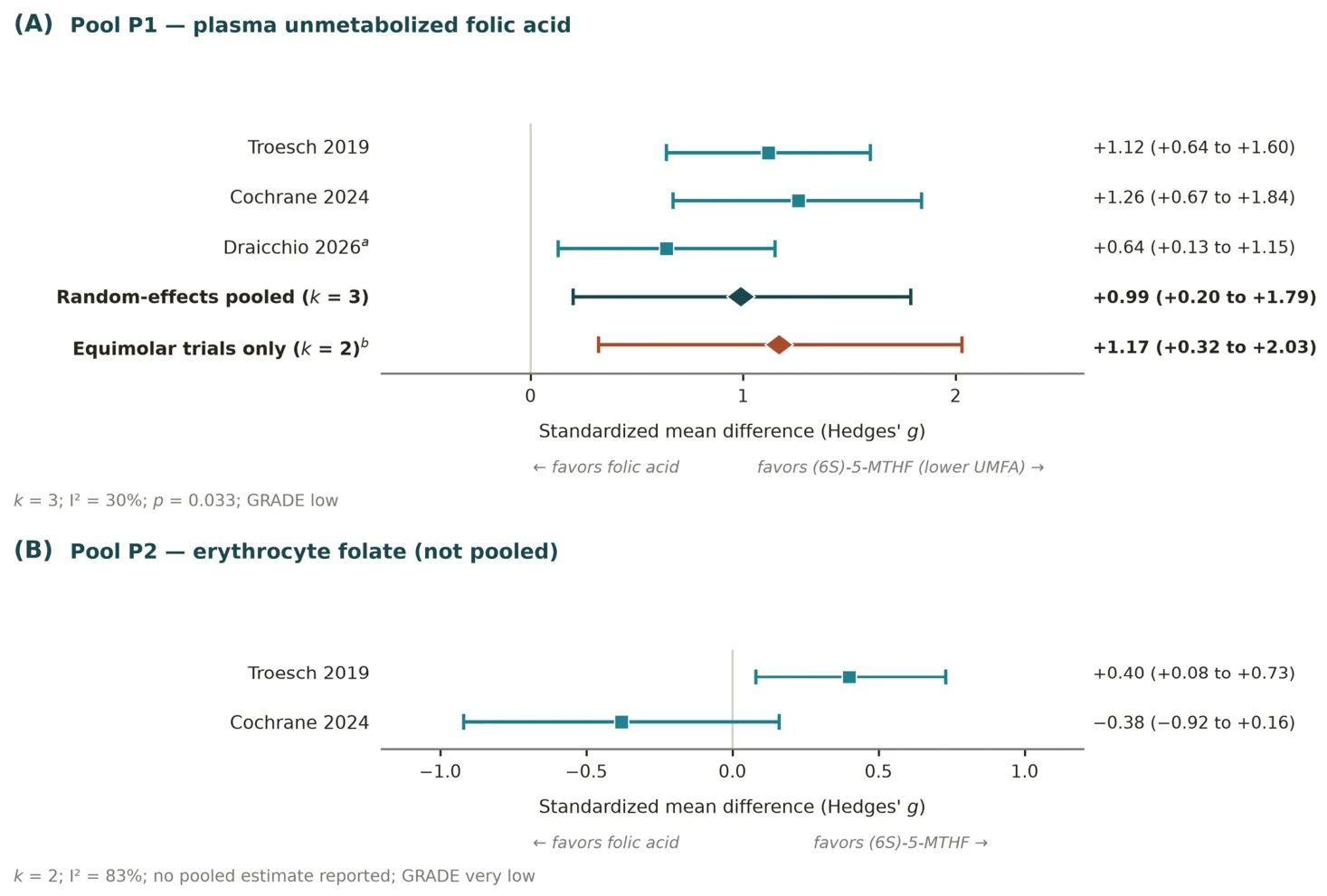
Study-level forest plot of the registered folic acid versus (6S)-5-MTHF comparisons. Panel (**A**): plasma unmetabolized folic acid (UMFA), Pool P1—the three contributing trials, the random-effects pooled estimate, and the prespecified-criterion sensitivity analysis restricted to the two strictly equimolar trials. Panel (**B**): erythrocyte folate, Pool P2—the two contributing trials shown individually; no pooled estimate is presented, because the trials are directionally discordant (*I*^2^ = 83%) and a two-study Hartung–Knapp interval is not informative. Squares show individual trial estimates and diamonds pooled or sensitivity estimates; horizontal lines are 95% Hartung–Knapp–Sidik–Jonkman confidence intervals. Pool P3 (plasma total homocysteine) is not displayed, because neither of its two eligible contributing reports supplies recoverable per-arm arithmetic means and standard deviations at the outcome time point; no pooled estimate was derived for that outcome, and it is reported qualitatively in Section 3.2. No clinical-irrelevance zone is shaded, because no minimal clinically important difference has been established for plasma total homocysteine (Section 4.5). ^a^ Retained under a declared post-registration amendment; this trial did not use strictly equimolar dosing (Section 2.1). ^b^ Leave-one-out sensitivity analysis, prespecified in the registered protocol as an analysis type and applied here at the registered equimolar-dose criterion, restricted to the two strictly equimolar trials [84,90] (Section 2.1). Effect sizes for the two median-reported trials were derived using the Wan et al. Method 3 estimator specified in Section 2.8. The per-arm analyzed denominators for plasma UMFA in Troesch 2019 [84] are an explicit assumption rather than a reported or recovered quantity; sensitivity of Panel (**A**) to that assumption, and the direction of bias introduced by limit-of-quantification censoring, are reported in Supplementary Table S4b. Study-level effect sizes for the secondary pools are given in Supplementary Figures S1, S3, S3b and S4. This figure is original and was newly generated by the authors from data extracted from the source publications [84,90,91]. Author-generated using Matplotlib (3.11.1); not adapted from any published figure.

The registered question comprised three prespecified outcome pools. Source-anchored extraction established that only one of them (Pool P1, plasma UMFA) contained the inputs required for a defensible quantitative synthesis; a random-effects meta-analysis with HKSJ adjustment was therefore performed for Pool P1 only, and Pools P2 and P3 are reported without pooled estimates for the specific reasons given below (Figure 2). Study-level inputs are given in Supplementary Table S4 for Pool P1 and Supplementary Table S5 for Pools P2 and P3.

Pool P1—plasma UMFA (*k* = 3). The pooled Hedges’ g SMD was +0.99 (95% HKSJ CI +0.20 to +1.79; *p* = 0.033; *I*^2^ = 30%; τ^2^ = 0.031), with all three head-to-head trials directionally concordant in favor of 5-MTHF [84,90,91] (Figure 2A). Both trials reporting medians were re-derived for this revision using the Wan et al. Method 3 estimator exactly as specified in Section 2.8 and Supplementary Methods Section S1. The infant-formula trial [84] also contributes a less precise estimate than in the originally submitted analysis: plasma UMFA at the final visit was analyzed in 124 of its 244 per-protocol infants rather than in the full randomized sample, because many samples fell below the limit of quantification. The source does not report how those 124 observations were distributed across the three groups, so the per-arm denominators used here are an explicit assumption rather than a recovered quantity, and both the allocation and the censoring it reflects are addressed by the sensitivity analyses in Supplementary Table S4b. Study-level inputs and their derivation are given in Supplementary Table S4. No prediction interval is reported, in accordance with the single prediction-interval rule stated in Section 2.8. The previously labeled Schön/Obeid 2024 entry [92] was removed from this pool because the source evaluates a 1:1 FA/(6S)-5-MTHF mixture rather than a head-to-head contrast.

One of the three trials, Draicchio 2026 [91], does not strictly satisfy the registered equimolar-dose criterion: the two prenatal multivitamins delivered approximately 588 µg (6S)-5-MTHF (1000 µg dietary folate equivalents, DFE) versus 800 µg FA (approximately 1330 µg DFE), a difference of roughly 40% on a molar basis, and the products also differed in other B vitamins, choline, and vitamin D. Its retention is declared as a post-registration protocol amendment (Section 2.1), and the corresponding prespecified-criterion equimolar-only sensitivity analysis excluding it preserves the direction and magnitude of the effect and eliminates heterogeneity: SMD +1.17 (95% HKSJ CI +0.32 to +2.03; *p* = 0.036; *I*^2^ = 0%; *k* = 2). The two remaining trials [84,90] both used strictly equimolar dosing, are directionally concordant, and yield an interval that excludes the null, so the registered effect does not depend on retaining the non-equimolar trial. That interval nonetheless remains wide and rests on a two-study HKSJ estimate carrying a single degree of freedom, and its lower bound is compatible with a small effect, so it constrains rather than establishes the magnitude (Supplementary Table S4).

Pool P2—erythrocyte folate (*k* = 2). Only two of the head-to-head trials measured erythrocyte folate [84,90]; Draicchio 2026 [91] measured serum, placental, and cord folate forms but not erythrocyte folate, and the human-milk report of the Cochrane trial describes the same randomized participants rather than an independent trial. The two eligible trials are directionally discordant. In the infant-formula trial [84], total red-cell folate at the final visit was higher with 5-MTHF (907.0 ± 192.8 in 69 infants versus 839.4 ± 142.4 nmol/L in 81 infants; Hedges’ g +0.40, 95% CI +0.08 to +0.73), whereas in the pregnancy trial [90] endline erythrocyte folate was numerically lower with 5-MTHF (1826 ± 471 in 29 participants versus 1998 ± 421 nmol/L in 25 participants; Hedges’ g −0.38, 95% CI −0.92 to +0.16). Per-arm analyzed denominators for both trials were recovered from the source publications rather than assumed, as documented in Supplementary Table S5. Heterogeneity between them is extreme (Q = 5.89; *I*^2^ = 83%), and a two-study Hartung–Knapp interval spans more than four standardized units; no pooled estimate is therefore reported (Figure 2B). In both trials erythrocyte folate remained well above deficiency thresholds—all participants in the pregnancy trial were above 906 nmol/L—but the adjusted between-group interval in that trial reached 400 nmol/L in favor of FA, and no non-inferiority margin was prespecified, the authors having described the trial as a pilot. These data are accordingly incapable of supporting an equivalence or non-inferiority claim for erythrocyte folate.

Pool P3—plasma tHcy (k = 2; not pooled). In the pregnancy trial [90]—the only randomized, equimolar, intention-to-treat head-to-head contrast in the pool—endline and postpartum tHcy are reported only in an online supplementary table that could not be retrieved, the article text stating only that all values remained below the hyperhomocysteinemia cut-off. The four-arm trial [93] reports homocysteine not in the retrieved 2006 publication but in its companion report [94], which gives a significant time × treatment interaction (*p* < 0.001) and no significant difference in tHcy reduction among the three supplemented arms (*p* > 0.05) without recoverable per-arm variances. Constructing a pooled estimate from these reports would require imputing distributional form and variance and would generate a numerically precise value that the primary sources do not support; the withholding rule stated in Section 2.8 therefore applies, and the registered tHcy outcome is reported qualitatively. Qualitatively, neither contributing report detected a statistically significant between-form difference in plasma tHcy, and the reported concentrations remained within the normal range, below the hyperhomocysteinemia threshold of 13 µmol/L. This is an absence of detected difference in two small, incompletely reported comparisons, and not a demonstration that the two folate forms are equivalent for this outcome. No minimal clinically important difference has been established for plasma tHcy, so the between-form differences visible in the individual reports (of the order of 0.1–0.6 µmol/L) cannot be characterized as either clinically negligible or clinically meaningful; for orientation only, they are of the same order as ordinary within-person biological variation in fasting tHcy, whose coefficient of variation of 8.3–23% [95,96] corresponds to roughly 0.4–0.7 µmol/L at the concentrations observed. As with erythrocyte folate, these data are incapable of supporting an equivalence or non-inferiority claim for tHcy: with no pooled estimate, no prespecified margin, and only two eligible comparisons, the absence of a detected difference reflects the limits of the available reporting as much as the underlying biology.

Collectively, the registered pools indicate that (6S)-5-MTHF substitution reduced circulating UMFA; that erythrocyte-folate findings are directionally discordant across the two trials measuring it and insufficient to establish equivalence or non-inferiority; and that neither of the two eligible reports detected a statistically significant between-form difference in plasma tHcy, although the contributing reports do not permit that outcome to be quantified in a pooled analysis and the absence of a detected difference is therefore uninformative about equivalence. Residual heterogeneity in P1 is plausibly explained by population, dose, duration, and assay generation rather than direction of effect, and is eliminated when the single non-equimolar trial is removed.

### 3.3. Secondary Quantitative Synthesis (Pools S1, S3, S4)

Pool S1—plasma 5-MTHF (*k* = 3; Supplementary Figure S1). The pooled SMD was +0.48 (95% HKSJ CI −0.80 to +1.76; *I*^2^ = 78%), directionally favoring 5-MTHF but imprecise owing to heterogeneity in assay sensitivity and dose [97]. With *k* = 3, no prediction interval is reported, in accordance with the single prediction-interval rule stated in Section 2.8; with *I*^2^ = 78%, this pool is hypothesis-generating only.

Pool S3—dose-response of FA on UMFA (*k* = 3; Supplementary Table S7 and Supplementary Figure S3). Three trials contributed: Murphy/FACT [45] (1 versus 4–5 mg/day in pregnancy), Tam [86] (1 versus 5 mg/day), and Fleming [98] (400 versus 800 µg/day in pregnancy), with per-trial mean differences of +11.3, −16.1, and +1.0 nmol/L, respectively. The pooled HKSJ MD was −1.2 nmol/L (95% CI −33.7 to +31.4; *I*^2^ = 85%). The pooled estimate is directionally null and hypothesis-generating, and the negative Tam estimate reflects a single low-dose outlier on the Wan range-estimated scale rather than a reported between-dose difference; the dose-response argument rests instead on trial-level pharmacology (Section 3.7). Excluding Tam post hoc, the pooled MD reverts to +4.6 nmol/L (95% HKSJ CI −58.0 to +67.2; *I*^2^ = 69%; Supplementary Figure S3b).

Pool S4—Methylenetetrahydrofolate reductase 677C→T × FA on tHcy (*k* = 3, genotype-stratified, not pooled; Supplementary Table S7 and Supplementary Figure S4). Of the three reports originally cited, only one [99] reports genotype-stratified change in tHcy: the CSPPT primary report [100] reports genotype-stratified folate but not tHcy, and the remaining report [26] specifies S-adenosylhomocysteine rather than tHcy as its outcome. The pool has been rebuilt from the three randomized reports that do supply the outcome, each entered once.

In the genotype-stratified trial of 1 mg/d for three months [99], the placebo-corrected reduction was −2.15 µmol/L (95% CI −3.02 to −1.28) in CC and −2.02 µmol/L (−2.55 to −1.49) in CT; its TT estimate is reported in Supplementary Table S7 but is not interpretable at face value because it is dominated by placebo-arm variability in 17 participants. In the genotype-stratified analysis of the large hypertension trial [101], the adjusted between-group reductions were −0.99 µmol/L (−1.21 to −0.76) in CC, −1.31 µmol/L (−1.49 to −1.12) in CT and −3.27 µmol/L (−3.84 to −2.70) in TT. In the eight-arm dose-ranging trial [102], the differences from the 0 mg arm at the matching 0.8 mg/d dose were −2.8, −1.5 and −5.1 µmol/L in CC, CT and TT, respectively. Because population, dose, duration, and the form of the reported estimates differ substantially across the three trials, no pooled genotype estimate or per-allele trend is reported. Across the three reports, TT participants generally showed a larger tHcy reduction than CC or CT participants, but the genotype pattern was not monotonic within every trial, and no pooled genotype trend was estimated.

### 3.4. Risk of Bias and Certainty for the Registered Question

Risk of bias for the head-to-head trials is tabulated in Supplementary Table S3, and the GRADE domain judgments and summary of findings for the registered question are given in Supplementary Table S8. Certainty for plasma UMFA (Pool P1) is low, downgraded once for serious risk of bias and once for serious imprecision. The risk-of-bias downgrade reflects that two of the three contributing trials are at high risk of bias for this outcome: in the infant-formula trial [84] plasma UMFA was analyzed in only 124 of 244 per-protocol infants, and in the non-equimolar trial [91] the only reported analysis was per protocol, with no intention-to-treat analysis. The imprecision downgrade reflects that the pooled interval extends from a small to a very large effect on only three trials with a combined analyzed sample of fewer than 200 participants for this outcome, and that its lower bound is additionally conditional on an assumed per-arm allocation in the largest trial (Supplementary Table S4b). The prespecified-criterion equimolar-only sensitivity analysis excludes the null, so it is no longer cited in support of this downgrade; the downgrade rests on the width of the interval and the fragility of its lower bound, not on non-significance. It was not downgraded for indirectness, and the reasoning is stated explicitly: the outcome is the analyte named in the registered PICO question rather than a proxy for it, so the comparison is direct with respect to population, intervention, comparator, and outcome as registered; the three trials, although drawn from different populations (infants and pregnant participants), were directionally concordant, and heterogeneity was low to moderate (*I*^2^ = 30%) and disappeared when the single non-equimolar trial was removed. It was not downgraded for inconsistency for the same reason. One qualification is stated openly rather than left implicit: the third trial [91] delivered a non-equimolar, multi-nutrient intervention, which is indirect with respect to the registered comparison of equimolar folate forms. Rather than settle that by aggregate judgment, the prespecified equimolar-only analysis supplies the estimate restricted to directly relevant evidence (+1.17, +0.32 to +2.03; k = 2), and it is concordant in direction and magnitude with the three-trial pool. Had indirectness instead been rated serious for the body of evidence as a whole, certainty for Pool P1 would fall to very low, and readers who take that view should read the rating accordingly. Population diversity in a pharmacokinetic contrast of this kind broadens applicability rather than making the evidence indirect. The counterweight is that UMFA is a surrogate analyte of uncertain clinical meaning: low certainty applies to the conclusion that substitution lowers circulating UMFA, and expressly not to any conclusion about clinical benefit. No patient-important clinical outcome was prespecified in the registered outcome set or synthesized and GRADE-rated in this review. Although the Troesch infant-formula trial reported growth and weight-gain outcomes, these were outside the registered PICO outcomes evaluated here. Certainty is very low for erythrocyte folate, downgraded once for serious inconsistency (directionally opposed estimates, *I*^2^ = 83%) and twice for very serious imprecision (*k* = 2; no pooled estimate is reportable at all, the two trial estimates point in opposite directions on a combined analyzed sample of 204 participants, and no non-inferiority margin was prespecified), so that a randomized body beginning at high certainty and downgraded three times yields very low, and very low for plasma tHcy, downgraded twice for very serious imprecision (no pooled estimate derivable from either contributing report, and no prespecified margin) and once for serious risk of bias arising from incomplete outcome reporting in both contributing reports [90,93]: endline values are available only in an unretrievable online supplement in one trial, and no recoverable per-arm variances are reported in the other. These ratings are not extended to the contextual literature, and publication bias could not be formally assessed in any pool.

### 3.5. Contextual Evidence I: Mechanistic Studies

Fifteen mechanistic analytic contributions were synthesized (Supplementary Table S1), with a small core of foundational enzymology and human pharmacokinetics underpinning a broader set of downstream observations.

The most robust finding concerns DHFR saturation. Human hepatic DHFR activity is about 1.4% that of rat hepatic DHFR per milligram of protein, with a roughly five-fold inter-individual range attributable in part to the DHFR 19-base-pair deletion polymorphism [8,19,103]. Two independent human pharmacokinetic studies converged on an approximate threshold of 200 µg of FA per eating occasion above which UMFA is commonly detected in serum within 90 min [15,16], although the threshold varies between individuals. Direct hepatic portal-vein sampling showed that approximately 80% of an oral 220 µg FA dose traverses the intestinal mucosa unmodified, whereas an equimolar dose of 5-formyl-THF appears in portal blood almost entirely as reduced folate [20]; the mucosa therefore appears functionally inactive as a site of FA reduction, with hepatic DHFR carrying most of the reductive burden.

Downstream effects build outward from this foundation. FBP-bound UMFA suppresses NK-cell cytotoxicity in vitro at concentrations as low as 1.0 nmol/L [23], and 5 mg/day FA for 90 days in healthy adults produced an 11.9-fold rise in plasma UMFA at day 45 with reduced NK cytotoxicity [24]. FRα blockade by FA was shown in vivo by [^18^F]AzaFol positron emission tomography in non-human primates at plasma concentrations of 63–90 ng/mL [21], far above fortification-level exposure, establishing competition at the receptor rather than a functional central folate deficit. In MTHFR 677CT and 677TT carriers, 2.4 mg/day FA elevated plasma SAH [26]. High FA consumption leads to pseudo-MTHFR deficiency, altered lipid metabolism, and liver injury in mice [104]. In mice, a 20-fold FA-supplemented diet produced ~10-fold higher maternal plasma folate with embryonic delay, growth retardation, and thinner ventricular walls [29], and other rodent studies report high-FA effects on methylation and embryonic development [27,28,105]; these exposures are superphysiologic and not directly translatable. Antifolate pharmacology supports a functional distinction between oxidized and reduced folates, leucovorin being a reduced-folate rescue agent after methotrexate or edatrexate exposure [106,107], and DHFR inhibition or saturation is expected to impair FA utilization disproportionately [8,108].

Overall, the mechanistic evidence supports a DHFR-saturation model of UMFA generation triangulated across enzyme kinetics, crossover pharmacokinetics, and portal-vein sampling. The evidence that circulating UMFA produces downstream biological consequences is weaker and rests largely on in vitro systems and supraphysiologic animal exposures.

### 3.6. Contextual Evidence II: Observational Studies

Twenty-four observational analytic contributions were included, classified by exposure metric in Table 1 and profiled individually in Supplementary Table S2. Findings are reported by class.

#### 3.6.1. Class 1—Directly Measured UMFA

Modern LC-MS/MS surveillance indicates that circulating UMFA is detectable in >95% of US serum samples; the proportion exceeding 1 nmol/L is substantially lower, including approximately 21% of fasting adults in NHANES 2007–2008. Older studies using different assays and detection thresholds reported lower prevalence estimates, particularly in fasting samples [18,47,48,109]. At standard prenatal doses of 400 µg/day, maternal UMFA remains modest and cord-blood UMFA is generally below the limit of quantification [51,52,110], while Obeid found FA > 0.20 nmol/L in 44% in maternal blood and 55% of cord blood in normal pregnancies [111]. whereas at the 4–5 mg/day doses prescribed for epilepsy and prior NTD pregnancies [57,58] maternal UMFA is ~2.5-fold higher than at 1 mg/day [45]. In the Canadian MIREC cohort, UMFA was detectable in nearly all participants, with higher concentrations at higher FA doses [14].

Only two cohorts have related directly measured UMFA to autism-related outcomes, and they disagree (Supplementary Table S6 and Supplementary Figure S2). In the Boston Birth Cohort (*n* = 567; 92 ASD cases), children in the highest cord-plasma UMFA quartile had higher ASD odds than the lowest (adjusted OR 2.26, 95% CI 1.08–4.75), an association carried by the Black subgroup (adjusted OR 9.85, 2.53–38.31) and null in non-Black children (0.85, 0.30–2.40) [33]. In the Norwegian MoBa antiseizure-medication sub-study (*n* = 227), maternal plasma UMFA at gestational weeks 17–19 was not associated with autistic traits at age 3 or 8 years [46]. The two studies differ in biospecimen, timing, and outcome ascertainment, so neither is a strict replication of the other; reviewing both, Mills and Molloy concluded that “the 2 studies on UMFA to date are contradictory, which speaks against a serious harmful effect” [76].

In older adults, cross-sectional NHANES analyses have repeatedly associated high folate status combined with low vitamin B_12_ with cognitive impairment and macrocytic anemia, an interaction that survived the transition to LC-MS/MS assay [30,48,49,50] and is mechanistically explicable as worsening of the methyl-folate trap when methionine synthase is functionally B_12_-deficient [112]. These analyses are cross-sectional and do not establish temporality.

#### 3.6.2. Class 2—Total Circulating Folate

In the Boston Birth Cohort, very high maternal plasma total folate at delivery (≥60.3 nmol/L) was associated with 2.5-fold ASD odds (95% CI 1.3–4.6) [32]. In a Swedish nested case-control study, higher maternal serum folate at gestational week 14 was associated with ASD (OR 1.70 per SD, 1.22–2.37) but did not survive false-discovery-rate correction across 62 biomarkers and was described by the authors as weak evidence [55]. In Generation R (*n* = 3893), maternal plasma folate was not associated with autistic traits or probable autism [53], and in the HOME study, maternal whole-blood folate was not associated with Social Responsiveness Scale scores [54]. In oncology, colorectal cancer survivors showed a hazard ratio of 1.31 (95% CI 1.02–1.58) for recurrence per twofold increase in post-treatment plasma FA [31], whereas the EPIC analysis of circulating folate forms and breast cancer found no main effect, only an interaction with alcohol intake [113].

#### 3.6.3. Class 3—Folic Acid Intake or Supplementation

This is much the largest class, and it is predominantly null or protective. Cohorts reporting protective associations include MoBa (*n* = 85,176; adjusted OR 0.61, 95% CI 0.41–0.90) [60], the Israeli Meuhedet cohort (*n* = 45,300; RR 0.27, 0.22–0.33) [61], MARBLES (adjusted RR 0.50, 0.30–0.81) [56], and CHARGE (≥600 versus <600 µg/day; adjusted OR 0.62, 0.42–0.92) [114]. Null findings come from the Stockholm Youth Cohort (*n* = 273,107; propensity-matched OR 1.10, 0.83–1.48) [62], the Danish National Birth Cohort (*n* = 87,210; adjusted HR 1.06, 0.94–1.19) [63], and a DNBC subsample [64]. High-dose exposure specifically has been examined in an Israeli nested case-control study drawn from 480,526 children using pharmacy-dispensed cumulative dose, with no significant case-control difference [65], and in the randomized FACT 4 Child follow-up of 4.0–5.1 mg/day FA versus placebo (*n* = 664 assessed at 4–9 years), in which an elevated score on at least one ASD-associated, executive-function, or behavioral measure occurred in 13.5% versus 14.8% (RR 0.91, 0.63–1.33) [115].

Meta-analyses of this class report consistently protective pooled estimates: RR 0.77 (0.64–0.93) [116], a reported 58% reduction across 756,365 children [117], OR 0.57 (0.46–0.72) [118], and RR 0.64 (0.46–0.90) [119]. A broader neurodevelopmental synthesis reported benefit at recommended doses while cautioning that “FA over-supplementation was not associated with an improvement and may have a negative impact” [120]. One Class 3 contribution examined a molecular rather than a neurodevelopmental outcome: in the Belgian MANOE cohort of 115 mother–infant pairs, maternal methyl-group donor intake was related to cord-blood DNA methylation at the *IGF2* differentially methylated region and at *DNMT1*, *LEP*, and *RXRA*. Neither UMFA nor LINE-1 methylation was measured, so this study cannot speak to UMFA specifically and is reported here for completeness of the exposure class [121].

The one high-dose signal in this class for a non-ASD neurodevelopmental outcome is the Spanish INMA cohort, in which ≥1000 µg/day periconceptional FA was associated with lower McCarthy verbal and verbal-memory scores at 4–5 years [66]; most markers in that analysis were null, and the positive findings could reflect multiple comparisons [76]. Finally, higher FA intake attenuates the association between prenatal pesticide exposure and ASD [122].

#### 3.6.4. Class 4—Population Fortification

Ecological analyses have reported temporal associations between fortification and colorectal neoplasia trends [67,68]. These are hypothesis-generating only and cannot be attributed to UMFA, and they sit against pooled randomized evidence in which FA supplementation over approximately five years did not increase overall or site-specific cancer incidence across roughly 50,000 participants [123]. The folate-timing hypothesis, under which folate status influences early neoplastic initiation and established lesions differently, is the most parsimonious reading of the divergence but remains a hypothesis.

#### 3.6.5. FRAA Studies

Studies of blocking and binding folate receptor α autoantibodies (FRAAs) bridge observational epidemiology and a candidate intervention pathway. Folate Receptor α autoantibodies seropositivity is reported in 75–76% of children with ASD in clinic-based series [34,35], but seropositivity is not equivalent to a documented cerebral folate deficit: the same autoantibodies are found in 75% of unaffected siblings and 29% of unrelated controls [35], titers fluctuate over time [37], CSF 5-MTHF is measured in only a minority of seropositive children [124], and the pooled prevalence of documented CFD in ASD is 38% (95% CI 11–71%; *I*^2^ = 92%) [36]. FRAA seropositivity is elevated in mothers of children with NTDs and in pedigrees segregating cerebral folate deficiency [125,126]. Unmetabolized folic acid generated by oral FA may compete with reduced folates at residual unblocked FRα; this is a coherent hypothesis that has not been shown to lower human CSF folate.

#### 3.6.6. Certainty of the Observational Evidence

Taken as a whole, the observational base spans OCEBM 2011 Levels 2a–5: 8 are Level 2b with one further report rated 2b/3b, 2 are Level 3b, 12 are Level 4 with one Level 5; per-study levels are recorded in Supplementary Table S2. For whether directly measured UMFA is associated with offspring ASD, the two available cohorts conflict and the evidence is best described as inconclusive. For whether FA supplementation at recommended doses is associated with offspring ASD, the evidence is consistent and predominantly protective.

### 3.7. Contextual Evidence III: Trial-Level UMFA Pharmacology

The interventional evidence comprised 32 analytic contributions (Supplementary Table S3), spanning randomized trials, randomized crossover trials, and non-randomized human interventions. In single-dose crossover designs, fasting UMFA was rarely detected at ≤100 µg of FA, appeared variably at 200 µg, became universal at 400 µg, and persisted at six weeks under chronic 1 mg/day dosing [15,16,86,127]. At 5 mg/day for 90 days, plasma UMFA rose approximately 11.9-fold by day 45, with a significant fall in NK cytotoxicity [24]. In older adults, 5 mg/day FA with vitamins B6 and B12 for three weeks raised median plasma UMFA from 0.08 to 15.3 nmol/L while lowering tHcy from 17.2 to 9.0 µmol/L [128], and at 400 µg/day FA alone produced detectable circulating UMFA more often than the same dose delivered in a B-complex (76% versus 33%) [127]. In pregnancy, the FACT ancillary sub-study showed ~2.5-fold higher UMFA at 4–5 mg/day than at 1 mg/day [45]. These trials converge on an approximate per-occasion threshold of 200 µg above which UMFA is commonly detected in serum, with the threshold itself varying between individuals.

None of the large cardiovascular and cognitive FA trials—HOPE-2 [129], VITATOPS [130], VISP [131], FACIT [132], NORVIT, B-PROOF [133], and CSPPT [100]—measured UMFA, although at doses of 0.8–5 mg/day UMFA was almost certainly elevated throughout; their broadly neutral results are, at minimum, not consistent with a large UMFA-mediated harm at those doses.

## 4. Discussion

### 4.1. Principal Findings

The registered answer is that substitution of equimolar (6S)-5-MTHF for synthetic FA appears to reduce circulating UMFA (SMD +0.99; 95% CI +0.20 to +1.79; *k* = 3; GRADE low; +1.17, +0.32 to +2.03 in the two strictly equimolar trials). The three registered outcomes are not supported at the same level and are not interchangeable. The UMFA result is registered, quantitatively pooled, and GRADE-rated. The erythrocyte-folate result is a registered secondary outcome that could not be pooled, because the only two trials measuring it are directionally discordant (*I*^2^ = 83%; GRADE very low); those two trials are insufficient to establish equivalence or non-inferiority in either direction. The tHcy result is a registered outcome that could not be quantified at all, because neither of the two eligible contributing reports supplies recoverable per-arm means and standard deviations; neither detected a statistically significant between-form difference, and no minimal clinically important difference has been established against which any difference could be judged (GRADE very low). With two to three trials per outcome, no prespecified non-inferiority margin, and no trial designed for that purpose, the evidence cannot support an equivalence or non-inferiority claim for either erythrocyte folate or tHcy; the absence of a detected difference reflects the limits of the available evidence and its reporting as much as the biology. The UMFA result itself is also sensitive to the exclusion of any single trial and rests in part on a trial in which the UMFA endpoint was measured in about half of the analyzed sample. The registered finding is therefore hypothesis-strengthening rather than confirmatory, and clinical benefit from substitution remains unestablished.

These two outcomes bear directly on what has historically been the central objection to reduced-folate substitution—that 5-MTHF would fail to deliver the hematologic and homocysteine-lowering benefits that justified fortification—but the available randomized data are too few and too incompletely reported to resolve it, and nothing in this synthesis should be read as having done so. For high-risk pregnancies routinely prescribed 4–5 mg/day FA, 5-MTHF substitution warrants formal evaluation in pre-registered confirmatory trials with attention to (6S) stereochemistry and to product equivalence between calcium L-5-MTHF and glucosamine-salt (6S)-5-MTHF formulations [108].

### 4.2. What Folic Acid Is, and Is Not, Established to Do

Folic acid is not an established ligand of any nuclear receptor, is not an established enzyme inhibitor outside the folate cycle and has no established direct genotoxic or epigenotoxic activity independent of the folate pool it enters. Its two established interactions, reduction by DHFR and binding to FBP/FRα [8,19,21,22], have not been shown to produce clinical harm in humans. FRα competition has been demonstrated in vitro [22,23] and in non-human primates at plasma FA concentrations exceeding those produced by fortification-level intake [21] but not linked to a clinical deficit. No study has shown that FA exposure lowers human CSF folate; the claim that UMFA depletes central folate is a mechanistic inference from FRα competition rather than an empirical CSF finding. The rodent and cell-culture literature reporting adverse effects generally uses exposures one to two orders of magnitude above human fortification-level intake and is mechanism-generating rather than translatable. The Centers for Disease Control and Prevention, which has authored and curated the principal safety syntheses in this field, states that circulating UMFA has had “no confirmed adverse effects” [13] and that “no confirmed health risks have been found” [134], consistent with the available evidence.

The properties that distinguish synthetic FA from the reduced folates are summarized in Table 2. The distinction is not one of potency but of metabolic route: FA requires enzymatic reduction before it can participate in one-carbon transfer, whereas 5-MTHF is already the circulating methyl-donor form, and only the former can appear in plasma as an unmetabolized species.

### 4.3. UMFA Versus Total Folate: An Identifiability Problem

A major methodological limitation of this field is that effects of UMFA cannot readily be identified independently of total circulating folate.

FA intake raises UMFA and total folate simultaneously and monotonically [14,15,16,45], so in observational data the two are strongly collinear and any association of UMFA with an outcome may equally reflect total folate, FA intake behavior, or an unmeasured correlate of supplement use. Studies of this design typically report downstream molecular endpoints without measuring UMFA, as in a Belgian cohort of 115 mother–infant pairs that related maternal methyl-group donor intake to cord-blood methylation at IGF2, DNMT1, LEP, and RXRA [121]. Unmetabolized folic acid also constitutes no more than 1–2% of serum total folate [76], making measurement error proportionally large relative to its dynamic range, and a single measurement conflates dose with time since last ingestion; in the Boston Birth Cohort, time since last FA exposure was not recorded [33,76].

**Figure 3:**
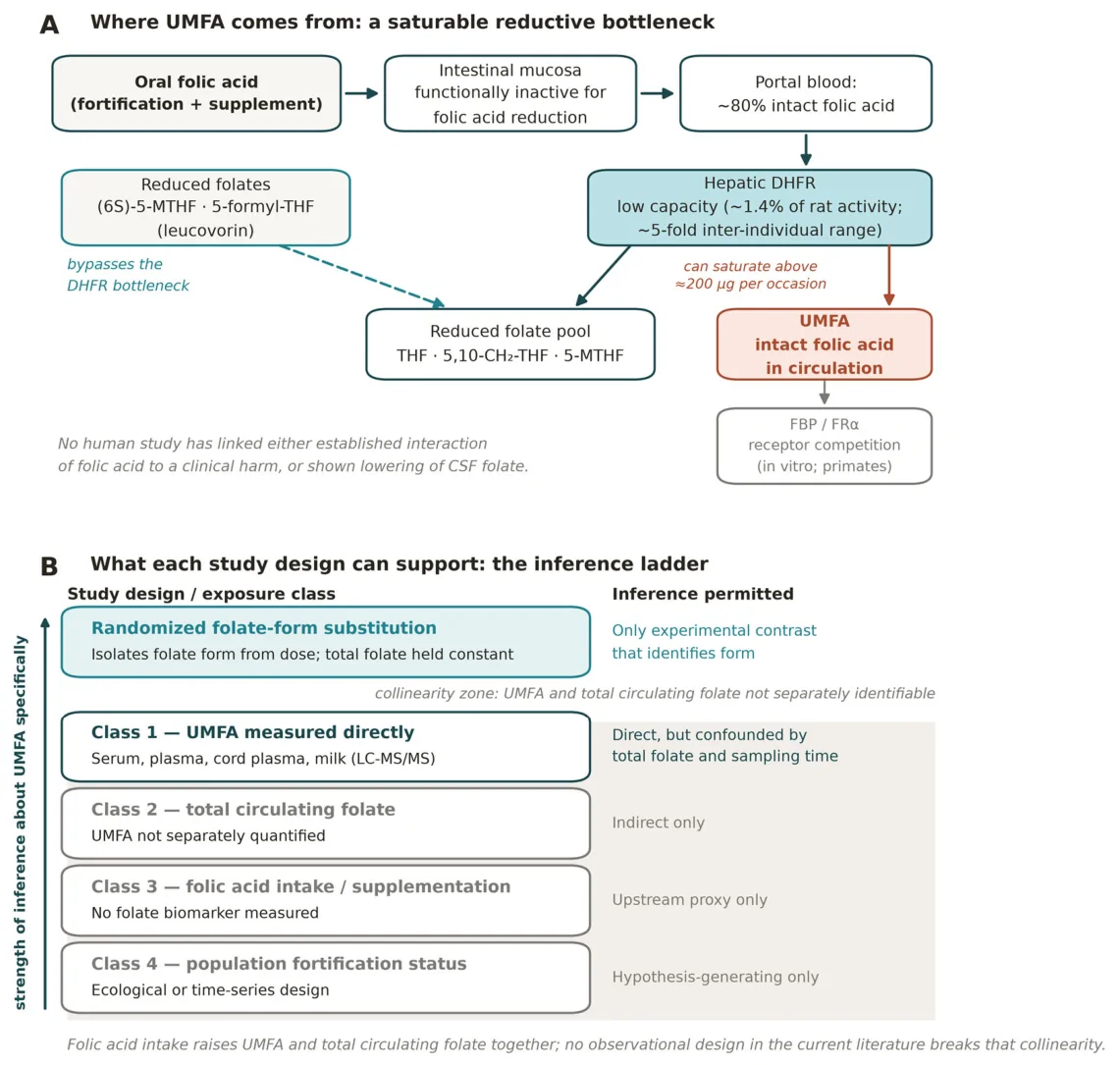
Conceptual framework illustrating (**A**) the generation of UMFA from folic acid through saturation of hepatic DHFR and (**B**) the hierarchy of study designs for inference regarding UMFA-specific effects. Randomized folate-form substitution provides the strongest evidence because it isolates folate form while minimizing confounding by total folate exposure. This figure is original and was newly generated by the authors and is not reproduced or adapted from any published figure.

Consequently, none of the Class 1 studies in Table 1 identifies a UMFA-specific effect (Figure 3). Designs that could in principle do so include within-stratum contrasts comparing UMFA across individuals matched or restricted on total folate, which requires cohorts large enough to break the collinearity; genetic instruments such as the DHFR 19-bp deletion or MTHFR 677C→T, which shift the UMFA:reduced-folate ratio at a given intake [8,103]; randomized folate-form substitution, as in Pools P1–P3, the only currently available experimental contrast that isolates form from dose; and standardized-timing pharmacokinetic sampling, which removes the time-since-ingestion component of measurement error. Future cohorts measuring UMFA should report total folate, reduced folates, fasting status, and time since last supplement as a minimum dataset.

### 4.4. Neurodevelopment in Context

The great majority of the prenatal folate–ASD literature reports null or protective associations. Approximately 497,000 pregnancies across ten non-overlapping prospective cohort and population-based analyses have been examined with null or protective findings [32,46,53,54,56,60,61,62,63,140], rising to roughly one million if the Norwegian nationwide registry and the Israeli 480,526-child dispensing cohort [65] are included, and four independent meta-analyses report pooled protective estimates [116,117,118,119]. No harm signal has been demonstrated in the 400–800 µg/day range delivered by fortification plus a standard prenatal supplement. The contrary hypothesis, that high FA intake contributes to ASD risk, has been argued in a previous narrative review [141] that drew chiefly on ecological and intake-based data and did not analyze directly measured UMFA; it is retained here for context rather than as a primary study.

The principal signal linking directly measured UMFA with ASD derives from a single preterm-enriched cohort of 567 children [33], which has several important limitations, including a time-since-ingestion problem and the small share of total folate that UMFA represents [76], birth order [142], no significant difference in cumulative dispensed FA dose between cases and controls [65], use of ICD code rather than adjudicated ascertainment, single-center retention, absence of maternal UMFA measurement, a preterm-enriched sample, and limited subgroup power [33]. In addition, the other cohort that measured circulating UMFA against ASD-related outcomes was inconclusive [46]. Furthermore, two independent studies failed to show a harm signal of high-dose FA [65,115].

Taken together, the evidence reviewed here provides no consistent signal of harm from prenatal FA at recommended doses and is predominantly consistent with benefit. FA supplementation above the recommended dose during pregnancy is not supported by current evidence. The cord-UMFA finding is best treated as a hypothesis requiring replication with LC-MS/MS quantification, adjudicated diagnoses, recorded time since ingestion, and adequate representation of the subgroup in which the signal was observed.

### 4.5. Vitamin B_12_ Dependence of Homocysteine Remethylation

**Figure 4:**
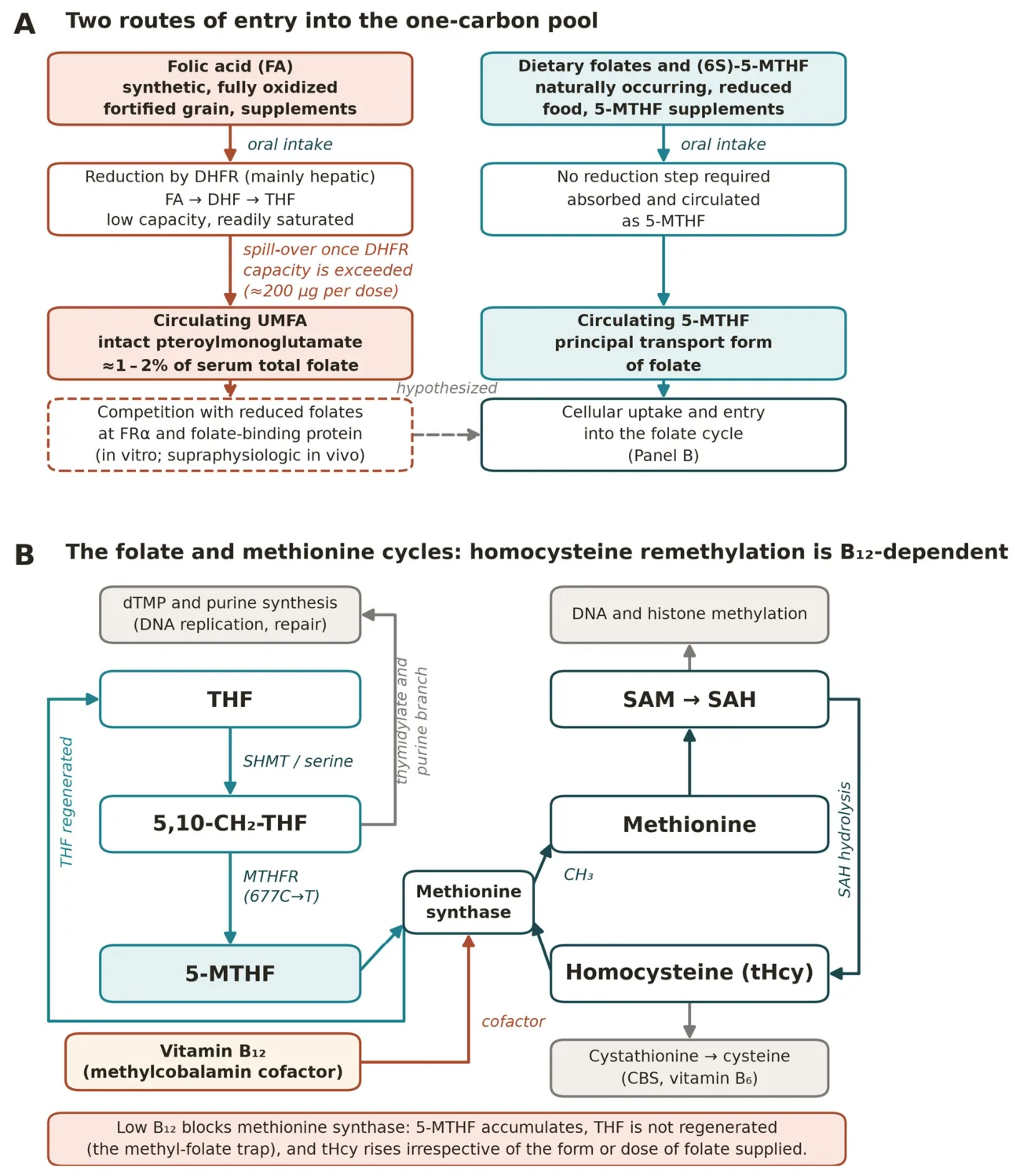
One-carbon metabolism, showing the divergent entry of synthetic folic acid and (6S)-5-MTHF into the folate cycle and the vitamin B_12_-dependent step at which homocysteine is remethylated to methionine. Folic acid must be reduced by DHFR before entering the cycle; when this capacity is exceeded, intact folic acid circulates as UMFA. (6S)-5-MTHF enters directly as the circulating methyl donor. Methionine synthase requires methylcobalamin, so when B_12_ is limiting, 5-MTHF accumulates (the methyl-folate trap), tHcy remains elevated regardless of folate form, and hematologic correction by folate can mask progressive B_12_-related neurological injury. This figure is original and was newly generated by the authors; it is a schematic synthesis of established pathway biochemistry [1,2,8,143] and is not reproduced or adapted from any published figure.

The tHcy endpoint used throughout this review is not a pure index of folate supply, and its interpretation requires explicit attention to vitamin B_12_. Remethylation of homocysteine to methionine is catalyzed by methionine synthase, which requires methylcobalamin as its cofactor and 5-MTHF as its methyl donor (Figure 4). When B_12_ is limiting, methionine synthase activity falls, 5-MTHF accumulates and cannot be recycled to tetrahydrofolate—the methyl-folate trap—and tHcy remains elevated irrespective of the amount or the form of folate provided [138,143]. A between-form comparison of tHcy therefore tests folate delivery only in participants whose B_12_ status is adequate; in B_12_-insufficient participants, neither FA nor 5-MTHF would be expected to normalize tHcy, and a null between-form difference carries no information about relative folate delivery. None of the trials contributing to Pool P3 reported B_12_-stratified results, which is an additional reason to read the null tHcy finding conservatively.

A second constraint on the tHcy endpoint is that it is a surrogate of uncertain clinical meaning. No minimal clinically important difference has been established for plasma tHcy, so no observed difference can be graded against a validated clinical benchmark; the differences observed in Pool P3 are, for orientation, of the same order as ordinary within-person biological variation [95,96]. Observationally, a tHcy concentration about 25% (approximately 3 µmol/L) lower is associated with roughly 11% lower ischemic heart disease and 19% lower stroke risk [144,145], yet pooled randomized evidence from 37,485 participants found that lowering tHcy with B vitamins did not reduce cardiovascular events, cancer, or mortality [146]. On that randomized evidence, and not on any comparison with biological variation, differences in tHcy of the magnitude seen here should not be interpreted as clinically meaningful in either direction. Equally, because no validated benchmark exists, they cannot be characterized as clinically negligible.

This dependence also underlies the principal safety concern attached to high folate exposure in the absence of demonstrated UMFA toxicity: masking. High folate intake corrects the megaloblastic anemia of B_12_ deficiency without correcting the neurological lesion, so hematologic normalization can delay diagnosis while subacute combined degeneration progresses. In NHANES, high serum folate with low B_12_ was associated with anemia (OR 3.1) and cognitive impairment (OR 2.6), whereas high folate with normal B_12_ was associated with lower odds of cognitive impairment (OR 0.4) [30]; higher serum folate in B_12_ deficiency was accompanied by higher tHcy and methylmalonic acid [138]; and in pooled data from three cohorts, low B_12_ with high folate carried an adjusted odds ratio of 3.45 for cognitive impairment [139]. In re-deriving the tolerable upper intake level for folate, the European Food Safety Authority judged the evidence insufficient to establish a causal folate–cognition relationship and set 1000 µg/day of supplemental folate, applying to (6S)-5-MTHF salts as well as to FA [137]; substituting a reduced folate does not remove the masking concern, because both forms replete the folate pool. The clinically actionable interaction in this field is therefore folate–B_12_ rather than UMFA itself: B_12_ status should be assessed before high-dose folate of any form is given to those at risk of B_12_ insufficiency, and tHcy should not be read as a folate-adequacy marker without a concurrent B_12_ measurement [143,147].

### 4.6. FRAA, Cerebral Folate Deficiency, and Leucovorin

In FRAA-positive patients, folate form may matter more than dose. Autoantibodies against FRα were originally described as a cause of cerebral folate deficiency, in which cerebrospinal fluid 5-MTHF is low despite normal serum folate [148], and the same autoantibodies were subsequently reported in low-functioning autism with neurological deficits [124]. Leucovorin uses the reduced folate carrier as an alternative transport pathway and therefore represents a mechanistically plausible reduced-folate strategy [135], and the US Food and Drug Administration approved a leucovorin calcium product for FRα-related cerebral folate transport deficiency on 10 March 2026 [136], after this manuscript was first submitted. It should be stated plainly, however, that no comparative trial has tested whether high-dose FA restores cerebrospinal fluid 5-MTHF when FRα is blocked. The inference that it does not rests on case reports and clinical summaries [36,39], not on a head-to-head comparison, and we do not claim more than that. Controlled-trial evidence in established ASD comprises four small, randomized trials [39,40,41,42] and a systematic review [36]. Comparators, endpoints, and blinding are heterogeneous and samples small, so this evidence is suggestive rather than confirmatory.

A weaker stream extends this axis into pregnancy. Because FRα also mediates transplacental folate transport, pre-conceptional FRAA screening with leucovorin substitution has been proposed as targeted prevention [149], supported only by two uncontrolled series [150,151] and a pilot randomized trial with a per-protocol sample of 18 [152]. No change to perinatal folate recommendations is warranted, and a multicenter confirmatory trial would be required before this signal could be interpreted further. A leucovorin calcium product is FDA approved for cerebral folate transport deficiency associated with FOLR1 dysfunction [136], not ASD, and its use in FRAA-positive ASD or pregnancy is a question for further trials.

### 4.7. Genotype-Aware Folate Strategy

Pool S4 suggests that TT participants may exhibit a larger homocysteine response to folic acid than CC or CT participants, although the genotype pattern was not monotonic within every trial and no pooled genotype trend was estimated. In TT homozygotes—about 10% of European-ancestry populations and a higher fraction of Mexican and Mediterranean-ancestry populations—the available evidence suggests that genotype-stratified studies of reduced folates are warranted [153]. This is a research question rather than a basis for genotype-guided supplementation in practice, and MTHFR 677TT carriers are a plausible early target population for confirmatory substitution trials.

### 4.8. Public Health Framing

A biological-versus-policy tension persists and should be stated plainly [154]. Folic acid fortification has prevented an enormous burden of NTDs and remains among the most cost-effective public-health interventions of the past century, and nothing in the UMFA literature supports discontinuing or weakening it. The question is one of stewardship: whether discretionary high-dose supplementation on top of fortification is justified in individuals who do not need it; whether high-risk pregnant women and FRAA-positive individuals should be evaluated for reduced-folate substitution in trials; and whether older adults with marginal B_12_ status warrant clinical attention to discretionary high-dose FA.

### 4.9. Limitations

Several limitations temper this synthesis. First and most importantly, UMFA and total circulating folate are not separately identifiable in the existing observational literature (Section 4.3), so the contextual observational findings should be read as concerning folate exposure generally. Second, the registered quantitative synthesis rests on two to three small trials per pool, precluding non-inferiority claims, prediction-interval interpretation, and formal small-study testing. Third, pre-2010 affinity-HPLC studies overestimate UMFA 2–3-fold through MeFox co-elution [73]. Fourth, fasting status and time since last ingestion were inconsistently reported, and none of the head-to-head trials stratified or reported outcomes by vitamin B_12_ status, which is a determinant of the tHcy endpoint (Section 4.5). Fifth, for erythrocyte folate, per-arm analyzed denominators were recovered by back-calculation from internally reported distributions and cross-checked against reported test statistics. For the Troesch plasma-UMFA endpoint, however, the arm-specific distribution of the 124 quantifiable observations is not reported and remains an explicit assumption; its effect is examined across the full plausible allocation range in Supplementary Table S4b.

Sixth, inter-individual variation is large, with the DHFR 19-bp deletion, MTHFR 677C→T, and rare FOLR1/FOLR2 variants all plausibly modifying effect, and few studies are genotype-stratified. Seventh, none of the large FA cardiovascular or cognitive trials measured UMFA. Eighth, the search was restricted to English-language publications. Ninth, the contextual streams were not GRADE-rated.

### 4.10. Research Priorities

A pre-registered multicenter pregnancy trial comparing FA and reduced-folate strategies, with cord-blood UMFA and maternal folate status as biomarker endpoints and registry-linked NTD and neurodevelopmental outcomes as clinical endpoints, would directly test the translational implications of this synthesis. A DHFR-genotype-stratified pharmacokinetic study in 19-bp deletion homozygotes versus reference would clarify inter-individual susceptibility. A trial in B_12_-insufficient older adults comparing FA, 5-MTHF, and B_12_ co-supplementation is a reasonable next step, although a long-duration trial powered for cognitive decline raises questions of equipoise and participant burden and would be more feasible as a biomarker-endpoint study nested within an existing cohort. A multicenter confirmatory trial of leucovorin versus FA in FRAA-positive first-trimester pregnancies is required before the pilot prevention signal can be taken further, and replication of the cord-UMFA × ASD analysis in an independent, adequately powered, LC-MS/MS-assayed cohort with adjudicated diagnoses would resolve the principal unreplicated finding in this field. Across all designs, UMFA should be measured by LC-MS/MS, fasting status and timing recorded, total folate and reduced folates reported alongside UMFA, and DHFR, MTHFR, and FRAA status ascertained at baseline.

## 5. Conclusions

This systematic review found that substitution of (6S)-5-MTHF for folic acid appears to reduce circulating UMFA, with low certainty: the three contributing trials are directionally concordant, but two are at high risk of bias for that outcome, the pooled interval extends from a small to a very large effect, and its lower bound is conditional on an assumed per-arm allocation in the largest trial (Supplementary Table S4b). For the two other registered outcomes, the evidence is weaker and asymmetric: the two trials measuring erythrocyte folate were directionally discordant and are insufficient to establish equivalence or non-inferiority, and for plasma tHcy neither eligible report detected a statistically significant between-form difference, but the contributing reports do not permit a pooled estimate, and the absence of a detected difference cannot be read as equivalence. The available randomized evidence is therefore limited and hypothesis-strengthening rather than confirmatory. Beyond that registered finding, current evidence does not establish that UMFA independently causes human harm: the principal established molecular interactions of intact FA that are relevant to proposed UMFA-specific effects are reduction by DHFR and binding to folate-binding proteins and FRα, neither of which has been shown to produce clinical harm; no study demonstrates that FA lowers CSF folate; and the prenatal neurodevelopmental literature is predominantly null or protective, with the single cord-UMFA harm signal unreplicated by the only other UMFA cohort. The findings of this review do not provide evidence to support changing current folic acid fortification policy. Future research should prioritize measuring UMFA properly—with attention to total folate, fasting status, timing, and genotype—and evaluating reduced-folate substitution in the specific populations where folate form, rather than folate dose, is mechanistically likely to matter.

## Figures and Tables

**Table 1:** Exposure classification framework applied to the observational literature on UMFA, with the inferential weight assigned to each class for the question of UMFA-specific effects. The studies named in each row are representative examples drawn from the cited observational literature and are not an exhaustive enumeration; the 24 observational analytic contributions that form the included evidence base are listed individually, with their exposure class, in Supplementary Table S2.

Class	Exposure Measured	Representative Studies	Inference Permitted About UMFA
1	Directly measured UMFA (serum, plasma, cord plasma, or human milk), by affinity-HPLC or LC-MS/MS	Raghavan 2020 (cord UMFA) [33]; Husebye 2022 (maternal plasma UMFA) [46]; NHANES surveillance series [18,47,48]; Morris 2010 [49]; Bailey 2020 [50]; Plumptre 2015 [51]; Sulistyoningrum 2024 [52]; Patti 2022 [14]	Direct, subject to design and confounding limits
2	Total circulating folate (serum, plasma, RBC, or cord total folate), UMFA not separately quantified	Morris 2007 [30]; Raghavan 2018 [32]; Steenweg-de Graaff 2015 [53]; Braun 2014 [54]; Egorova 2020 [55]; Geijsen 2020 [31]	Indirect only; cannot separate UMFA from total folate (see Section 4.3)
3	Folic acid intake or supplementation (self-reported, dispensing-record, or prescribed dose), no folate biomarker	Schmidt 2019 [56]; Alvestad 2022 [57]; Bjørk 2018 [58]; Figueiredo 2009 [59]; Surén 2013 [60]; Levine 2018 [61]; DeVilbiss 2017 [62]; Strøm 2018 [63]; Virk 2016 [64]; Sharman Moser 2019 [65]; Valera-Gran 2017 [66]	Upstream proxy only; effect cannot be attributed to UMFA
4	Population fortification status (ecological or time-series)	Hirsch 2009 [67]; Mason 2007 [68]	Ecological; hypothesis-generating only

**Table 2:** Comparison of synthetic folic acid and reduced folates. This table summarizes established biochemical and regulatory properties for orientation; it is not a summary of comparative clinical performance, which the registered evidence does not establish (Section 3.2 and Section 4.1).

Property	Synthetic Folic Acid (Pteroylmonoglutamic Acid)	Reduced Folates (Natural Food Folates; (6S)-5-MTHF; Leucovorin)
Chemical nature	Fully oxidized, synthetic; not a physiological folate	Reduced (tetrahydro) forms; physiological
Natural occurrence	Does not occur naturally in food; fortification and supplements only	Predominant forms in food and in human plasma and tissue [1,2]
Metabolic requirement before use	Must be reduced by DHFR to DHF then THF; hepatic DHFR capacity is low and appears readily saturated under common supplemental exposures [8,19]	Enter the folate cycle directly; (6S)-5-MTHF is the circulating methyl donor; leucovorin (5-formyl-THF) is interconverted without DHFR [135]
Potential to generate UMFA	Yes—intact folic acid circulates when reduction capacity is exceeded [14,15,16,45]	No—no unmetabolized synthetic species is generated [84,90,91]
Transport into cells and CNS	Uses the reduced folate carrier, PCFT, and FRα; intact folic acid binds FBP and FRα and can compete with reduced folates for receptor occupancy [21,22]	Use the same transporters; leucovorin can enter through the reduced folate carrier when FRα is blocked [135]
Biological activity	No established one-carbon coenzyme activity until reduced; intact FA can bind FBP/FRα	Directly active as methyl donor (5-MTHF) or as a THF-pool precursor (leucovorin)
Homocysteine remethylation	Supports remethylation once reduced to 5-MTHF; requires vitamin B_12_ as the methionine-synthase cofactor (Section 4.5)	Supports remethylation without a reduction step; equally requires vitamin B_12_ (Section 4.5). The randomized evidence reviewed here does not establish comparative performance between the forms (Section 3.2)
Stereochemistry	Single synthetic compound	(6S) is the biologically active diastereomer; racemic preparations deliver an inactive (6R) fraction [108]
Established clinical role	Neural tube defect prevention through fortification and periconceptional supplementation; the evidence base for public-health policy [9,10,11,12]	Rescue and repletion therapy [135]. A leucovorin calcium product is FDA-approved for cerebral folate transport deficiency associated with FOLR1 dysfunction [136].
Regulatory upper intake level	1000 µg/day of supplemental folate [137]	EFSA established an adult UL equivalent to 1000 µg/day folic acid and extended the UL framework to authorized (6S)-5-MTHF salts; see EFSA for form-specific expression [137]
Vitamin B_12_ masking risk	Present—corrects megaloblastic anemia without correcting neurological injury [30,138,139]	Also present; substituting a reduced folate does not remove this risk, because both forms replete the folate pool [137]

## Data Availability

All data underlying this Review are distributed as Supplementary Materials. The analytic code (meta_analyses.py; Python 3.11, numpy 1.26, scipy 1.12) is described in Supplementary Methods Section S8 and is available from the corresponding author on reasonable request. The review protocol is publicly registered at PROSPERO (CRD420261407616).

## Supplementary Materials

The following supporting information can be downloaded at: https://www.mdpi.com/article/10.3390/nu18172887/s1, Supplementary Methods: effect-size construction; conversion of reported summary statistics; random-effects pooling and the Hartung–Knapp–Sidik–Jonkman small-sample adjustment; heterogeneity quantification; duplicate-cohort handling; protocol deviations and post-hoc analyses; prespecified sensitivity analyses; study-data extraction rules; software and reproducibility; and the search strategy with final search strings per source. The Supplementary Methods cite references [15,19,39,43,44,45,59,77,78,79,80,81,82,83,84,86,88,89,90,91,92,98,123,155,156,157,158]. Supplementary Table S1: descriptive evidence profile, mechanistic studies (*n* = 15). Supplementary Table S2: descriptive evidence profile, human observational studies (*n* = 24), with exposure classification. Supplementary Table S3: descriptive evidence profile, interventional trials (*n* = 32), including RoB 2.0 and OCEBM 2011 judgments. Supplementary Table S4: Pool P1 study-level inputs (plasma UMFA). Supplementary Table S5: Pool P2 (erythrocyte folate) and Pool P3 (plasma total homocysteine) study-level inputs. Supplementary Table S6: cord-blood UMFA × ASD evidence from the retrieved cohort source. Supplementary Table S7: Pool S3 and Pool S4 study-level inputs. Supplementary Table S8: GRADE summary-of-findings for the registered folic acid versus (6S)-5-MTHF question. Supplementary Table S9: PRISMA 2020 checklist. Supplementary Table S10: Row-level disposition of the originally submitted evidence ledger. Supplementary Figure S1: Pool S1 forest plot (plasma 5-MTHF). Supplementary Figure S2: cord-blood UMFA category × ASD, Boston Birth Cohort. Supplementary Figure S3: Pool S3 forest plot (high-dose versus standard folic acid). Supplementary Figure S4: Pool S4 three-genotype panel (MTHFR 677C→T × folic acid on plasma total homocysteine).

## Abbreviations

The following abbreviations are used in this manuscript:

Abbreviation	Definition
5,10-CH_2_-THF	5,10-methylenetetrahydrofolate
5-formyl-THF	5-formyltetrahydrofolate (folinic acid; leucovorin)
5-MTHF	5-methyltetrahydrofolate
aHR	adjusted hazard ratio
aOR	adjusted odds ratio
aRR	adjusted risk ratio
ASD	autism spectrum disorder
CFD	cerebral folate deficiency
CI	confidence interval
CSF	cerebrospinal fluid
DHF	dihydrofolate
DHFR	dihydrofolate reductase
FA	folic acid (synthetic pteroylmonoglutamic acid)
FBP	folate-binding protein
FOLR1	folate receptor 1 gene
FRAA	folate receptor alpha autoantibody
FRα	folate receptor alpha
GRADE	Grading of Recommendations, Assessment, Development and Evaluations
HKSJ	Hartung–Knapp–Sidik–Jonkman
HPLC	high-performance liquid chromatography
*I*^2^	proportion of total variability attributable to between-study heterogeneity
LC-MS/MS	liquid chromatography with tandem mass spectrometry
MD	mean difference
MeFox	pyrazino-*s*-triazine derivative of 4α-hydroxy-5-methyltetrahydrofolate
MTHFR	methylenetetrahydrofolate reductase
NHANES	National Health and Nutrition Examination Survey
NK	natural killer (cell)
NTD	neural tube defect
OCEBM	Oxford Centre for Evidence-Based Medicine
OR	odds ratio
PCFT	proton-coupled folate transporter
PICO	population, intervention, comparator, outcome
PRISMA	Preferred Reporting Items for Systematic Reviews and Meta-Analyses
PROSPERO	International Prospective Register of Systematic Reviews
RCT	randomized controlled trial
RFC	reduced folate carrier
RoB 2	Cochrane Risk of Bias tool, version 2
ROBINS-I	Risk Of Bias In Non-randomized Studies of Interventions
RR	risk ratio
SAH	*S*-adenosylhomocysteine
SAM	*S*-adenosylmethionine
SD	standard deviation
SE	standard error
SMD	standardized mean difference
SYRCLE	Systematic Review Centre for Laboratory Animal Experimentation
tHcy	total homocysteine
THF	tetrahydrofolate
UMFA	unmetabolized folic acid
τ^2^	between-study variance

## References

[1] Ducker G.S., Rabinowitz J.D. (2017). One-carbon metabolism in health and disease. Cell Metab..

[2] Lyon P., Strippoli V., Fang B., Cimmino L. (2020). B vitamins and one-carbon metabolism: Implications in human health and disease. Nutrients.

[3] Woeller C.F., Anderson D.D., Szebenyi D.M., Stover P.J. (2007). Evidence for small ubiquitin-like modifier-dependent nuclear import of the thymidylate biosynthesis pathway. J. Biol. Chem..

[4] Kelly G.S. (1998). Folates: Supplemental forms and therapeutic applications. Altern. Med. Rev..

[5] Christensen K.E., Deng L., Leung K.Y., Arning E., Bottiglieri T., Malysheva O.V., Caudill M.A., Krupenko N.I., Greene N.D., Jerome-Majewska L. (2013). A novel mouse model for genetic variation in 10-formyltetrahydrofolate synthetase exhibits disturbed purine synthesis with impacts on pregnancy and embryonic development. Hum. Mol. Genet..

[6] Bailey L.B., Stover P.J., McNulty H., Fenech M.F., Gregory J.F., Mills J.L., Pfeiffer C.M., Fazili Z., Zhang M., Ueland P.M. (2015). Biomarkers of Nutrition for Development—Folate Review. J. Nutr..

[7] Visentin M., Diop-Bove N., Zhao R., Goldman I.D. (2014). The intestinal absorption of folates. Annu. Rev. Physiol..

[8] Abali E.E., Skacel N.E., Celikkaya H., Hsieh Y.C. (2008). Regulation of human dihydrofolate reductase activity and expression. Vitam. Horm..

[9] MRC Vitamin Study Research Group (1991). Prevention of neural tube defects: Results of the Medical Research Council Vitamin Study. Lancet.

[10] Williams J., Mai C.T., Mulinare J., Isenburg J., Flood T.J., Ethen M., Frohnert B., Kirby R.S. (2015). Updated estimates of neural tube defects prevented by mandatory folic acid fortification—United States, 1995–2011. MMWR Morb. Mortal. Wkly. Rep..

[11] Bailey R.L., Dodd K.W., Gahche J.J., Dwyer J.T., McDowell M.A., Yetley E.A., Sempos C.A., Burt V.L., Radimer K.L., Picciano M.F. (2010). Total folate and folic acid intake from foods and dietary supplements in the United States: 2003–2006. Am. J. Clin. Nutr..

[12] Bailey R.L., Pac S.G., Fulgoni V.L., Reidy K.C., Catalano P.M. (2019). Estimation of total usual dietary intakes of pregnant women in the United States. JAMA Netw. Open..

[13] Crider K.S., Qi Y.P., Yeung L.F., Mai C.T., Head Zauche L., Wang A., Daniels K., Williams J.L. (2022). Folic acid and the prevention of birth defects: 30 years of opportunity and controversies. Annu. Rev. Nutr..

[14] Patti M.A., Braun J.M., Arbuckle T.E., MacFarlane A.J. (2022). Associations between folic acid supplement use and folate status biomarkers in the first and third trimesters of pregnancy in the Maternal-Infant Research on Environmental Chemicals (MIREC) Pregnancy Cohort Study. Am. J. Clin. Nutr..

[15] Kelly P., McPartlin J., Goggins M., Weir D.G., Scott J.M. (1997). Unmetabolized folic acid in serum: Acute studies in subjects consuming fortified food and supplements. Am. J. Clin. Nutr..

[16] Sweeney M.R., McPartlin J., Weir D.G., Scott J.M. (2007). Folic acid fortification and public health: Report on threshold doses above which unmetabolized folic acid appear in serum. BMC Public Health.

[17] Sweeney M.R., McPartlin J., Weir D.G., Daly L., Scott J.M. (2006). Postprandial serum folic acid response to multiple doses of folic acid in fortified bread. Br. J. Nutr..

[18] Pfeiffer C.M., Sternberg M.R., Fazili Z., Yetley E.A., Lacher D.A., Bailey R.L., Johnson C.L. (2015). Unmetabolized folic acid is detected in nearly all serum samples from US children, adolescents, and adults. J. Nutr..

[19] Bailey S.W., Ayling J.E. (2009). The extremely slow and variable activity of dihydrofolate reductase in human liver and its implications for high folic acid intake. Proc. Natl. Acad. Sci. USA.

[20] Patanwala I., King M.J., Barrett D.A., Rose J., Jackson R., Hudson M., Philo M., Dainty J.R., Wright A.J., Finglas P.M. (2014). Folic acid handling by the human gut: Implications for food fortification and supplementation. Am. J. Clin. Nutr..

[21] Bagdasarian F.A., Guma E., Tokala R., Yoo C.-H., Downey J.W., Hooker J.M., Zürcher N.R., Wey H.-Y. (2026). [18F]AzaFol PET captures folic acid dynamics at folate receptors in the brain. J. Cereb. Blood Flow Metab..

[22] Sirotnak F.M., Tolner B. (1999). Carrier-mediated membrane transport of folates in mammalian cells. Annu. Rev. Nutr..

[23] Troen A.M., Mitchell B., Sorensen B., Wener M.H., Johnston A., Wood B., Selhub J., McTiernan A., Yasui Y., Oral E. (2006). Unmetabolized folic acid in plasma is associated with reduced natural killer cell cytotoxicity among postmenopausal women. J. Nutr..

[24] Paniz C., Bertinato J.F., Lucena M.R., De Carli E., Amorim P.M.S., Gomes G.W., Palchetti C.Z., Figueiredo M.S., Pfeiffer C.M., Fazili Z. (2017). A daily dose of 5 mg folic acid for 90 days is associated with increased serum unmetabolized folic acid and reduced natural killer cell cytotoxicity in healthy Brazilian adults. J. Nutr..

[25] Partearroyo T., Ubeda N., Montero A., Achon M., Varela-Moreiras G. (2013). Vitamin B12 and folic acid imbalance modifies NK cytotoxicity, lymphocytes B and lymphoproliferation in aged rats. Nutrients.

[26] Zhang R.S., Tang L., Zhang Y., Shi X.L., Shu J., Wang L., Zhang X., Xu Y.-P., Zou J.-F., Wang R. (2022). Effect of folic acid supplementation on the change of plasma S-adenosylhomocysteine level in Chinese hypertensive patients: A randomized, double-blind, controlled clinical trial. J. Clin. Biochem. Nutr..

[27] Barua S., Chadman K.K., Kuizon S., Buenaventura D., Stapley N.W., Ruocco F., Begum U., Guariglia S.R., Brown W.T., Junaid M.A. (2014). Increasing maternal or post-weaning folic acid alters gene expression and moderately changes behavior in the offspring. PLoS ONE.

[28] Mikael L.G., Deng L., Paul L., Selhub J., Rozen R. (2013). Moderately high intake of folic acid has a negative impact on mouse embryonic development. Birth Defects Res. A Clin. Mol. Teratol..

[29] Pickell L., Brown K., Li D., Wang X.L., Deng L., Wu Q., Selhub J., Luo L., Jerome-Majewska L., Rozen R. (2011). High intake of folic acid disrupts embryonic development in mice. Birth Defects Res. A Clin. Mol. Teratol..

[30] Morris M.S., Jacques P.F., Rosenberg I.H., Selhub J. (2007). Folate and vitamin B-12 status in relation to anemia, macrocytosis, and cognitive impairment in older Americans in the age of folic acid fortification. Am. J. Clin. Nutr..

[31] Geijsen A.J.M.R., Ulvik A., Gigic B., Kok D.E., van Duijnhoven F.J.B., Holowatyj A.N., Brezina S., van Roekel E.H., Baierl A., Bergmann M.M. (2020). Circulating folate and folic acid concentrations: Associations with colorectal cancer recurrence and survival. JNCI Cancer Spectr..

[32] Raghavan R., Riley A.W., Volk H., Caruso D., Hironaka L., Sices L., Hong X., Wang G., Ji Y., Brucato M. (2018). Maternal multivitamin intake, plasma folate and vitamin B12 levels and autism spectrum disorder risk in offspring. Paediatr. Perinat. Epidemiol..

[33] Raghavan R., Selhub J., Paul L., Ji Y., Wang G., Hong X., Zuckerman B., Fallin M.D., Wang X. (2020). A prospective birth cohort study on cord blood folate subtypes and risk of autism spectrum disorder. Am. J. Clin. Nutr..

[34] Frye R.E., Sequeira J.M., Quadros E.V., James S.J., Rossignol D.A. (2013). Cerebral folate receptor autoantibodies in autism spectrum disorder. Mol. Psychiatry.

[35] Quadros E.V., Sequeira J.M., Brown W.T., Mevs C., Marchi E., Flory M., Jenkins E.C., Velinov M.T., Cohen I.L. (2018). Folate receptor autoantibodies are prevalent in children diagnosed with autism spectrum disorder, their normal siblings and parents. Autism Res..

[36] Rossignol D.A., Frye R.E. (2021). Cerebral folate deficiency, folate receptor alpha autoantibodies and leucovorin (folinic acid) treatment in autism spectrum disorders: A systematic review and meta-analysis. J. Pers. Med..

[37] Ramaekers V.T., Sequeira J.M., Quadros E.V. (2016). The basis for folinic acid treatment in neuro-psychiatric disorders. Biochimie.

[38] Ramaekers V.T., Segers K., Sequeira J.M., Koenig M., Van Maldergem L., Bours V., Kornak U., Quadros E. (2018). Genetic assessment and folate receptor autoantibodies in infantile-onset cerebral folate deficiency (CFD) syndrome. Mol. Genet. Metab..

[39] Frye R.E., Slattery J., Delhey L., Furgerson B., Strickland T., Tippett M., Sailey A., Wynne R., Rose S., Melnyk S. (2018). Folinic acid improves verbal communication in children with autism and language impairment: A randomized double-blind placebo-controlled trial. Mol. Psychiatry.

[40] Batebi N., Moghaddam H.S., Hasanzadeh A., Fakour Y., Mohammadi M.R., Akhondzadeh S. (2021). Folinic acid as adjunctive therapy in treatment of inappropriate speech in children with autism: A double-blind and placebo-controlled randomized trial. Child Psychiatry Hum. Dev..

[41] Renard E., Leheup B., Guéant-Rodriguez R.M., Oussalah A., Quadros E.V., Guéant J.L. (2020). Folinic acid improves the score of autism in the EFFET placebo-controlled randomized trial. Biochimie.

[42] Zhang C., Chen Y., Hou F., Li Y., Wang W., Guo L., Zhang C., Li L., Lu C. (2025). Safety and efficacy of high-dose folinic acid in children with autism: The impact of folate metabolism gene polymorphisms. Nutrients.

[43] Page M.J., McKenzie J.E., Bossuyt P.M., Boutron I., Hoffmann T.C., Mulrow C.D., Shamseer L., Tetzlaff J.M., Akl E.A., Brennan S.E. (2021). The PRISMA 2020 statement: An updated guideline for reporting systematic reviews. BMJ.

[44] Howick J., Chalmers I., Glasziou P., Greenhalgh T., Heneghan C., Liberati A., Moschetti I., Phillips B., Thornton H., Goddard O. (2011). The 2011 Oxford CEBM Levels of Evidence (Introductory Document).

[45] Murphy M.S.Q., Muldoon K.A., Sheyholislami H., Behan N., Lamers Y., Rybak N., White R.R., Harvey A.L.J., Gaudet L.M., Smith G.N. (2021). Impact of high-dose folic acid supplementation in pregnancy on biomarkers of folate status and 1-carbon metabolism: An ancillary study of the Folic Acid Clinical Trial (FACT). Am. J. Clin. Nutr..

[46] Husebye E.S.N., Wendel A.W.K., Gilhus N.E., Riedel B., Bjørk M.H. (2022). Plasma unmetabolized folic acid in pregnancy and risk of autistic traits and language impairment in antiseizure medication-exposed children of women with epilepsy. Am. J. Clin. Nutr..

[47] Kalmbach R.D., Choumenkovitch S.F., Troen A.M., D’Agostino R., Jacques P.F., Selhub J. (2008). Circulating folic acid in plasma: Relation to folic acid fortification. Am. J. Clin. Nutr..

[48] Bailey R.L., Mills J.L., Yetley E.A., Gahche J.J., Pfeiffer C.M., Dwyer J.T., Dodd K.W., Sempos C.T., Betz J.M., Picciano M.F. (2010). Unmetabolized serum folic acid and its relation to folic acid intake from diet and supplements in a nationally representative sample of adults aged ≥60 y in the United States. Am. J. Clin. Nutr..

[49] Morris M.S., Jacques P.F., Rosenberg I.H., Selhub J. (2010). Circulating unmetabolized folic acid and 5-methyltetrahydrofolate in relation to anemia, macrocytosis, and cognitive test performance in American seniors. Am. J. Clin. Nutr..

[50] Bailey R.L., Jun S., Murphy L., Green R., Gahche J.J., Dwyer J.T., Potischman N., McCabe G.P., Miller J.W. (2020). High folic acid or folate combined with low vitamin B-12 status: Potential but inconsistent association with cognitive function in a nationally representative cross-sectional sample of US older adults participating in the NHANES. Am. J. Clin. Nutr..

[51] Plumptre L., Masih S.P., Ly A., Aufreiter S., Sohn K.J., Croxford R., Lausman A.Y., Berger H., O’Connor D.L., Kim Y.I. (2015). High concentrations of folate and unmetabolized folic acid in a cohort of pregnant Canadian women and umbilical cord blood. Am. J. Clin. Nutr..

[52] Sulistyoningrum D.C., Sullivan T.R., Skubisz M., Palmer D.J., Wood S., Ueland P.M., McCann A., Makrides M., Green T.J., Best K.P. (2024). Maternal serum unmetabolized folic acid concentration following multivitamin and mineral supplementation with or without folic acid after 12 weeks gestation: A randomized controlled trial. Matern. Child Nutr..

[53] Steenweg-de Graaff J., Ghassabian A., Jaddoe V.W.V., Tiemeier H., Roza S.J. (2015). Folate concentrations during pregnancy and autistic traits in the offspring. The Generation R Study. Eur. J. Public Health.

[54] Braun J.M., Froehlich T., Kalkbrenner A., Pfeiffer C.M., Fazili Z., Yolton K., Lanphear B.P. (2014). Brief report: Are autistic-behaviors in children related to prenatal vitamin use and maternal whole blood folate concentrations?. J. Autism Dev. Disord..

[55] Egorova O., Myte R., Schneede J., Hägglöf B., Bölte S., Domellöf E., Ivars A’roch B., Elgh F., Ueland P.M., Silfverdal S.A. (2020). Maternal blood folate status during early pregnancy and occurrence of autism spectrum disorder in offspring: A study of 62 serum biomarkers. Mol. Autism.

[56] Schmidt R.J., Iosif A.M., Guerrero Angel E., Ozonoff S. (2019). Association of maternal prenatal vitamin use with risk for autism spectrum disorder recurrence in young siblings. JAMA Psychiatry.

[57] Alvestad S., Husebye E.S.N., Christensen J., Dreier J.W., Sun Y., Igland J., Leinonen M.K., Gissler M., Gilhus N.E., Tomson T. (2022). Folic acid and risk of preterm birth, preeclampsia, and fetal growth restriction among women with epilepsy: A prospective cohort study. Neurology.

[58] Bjørk M., Riedel B., Spigset O., Veiby G., Kolstad E., Daltveit A.K., Gilhus N.E. (2018). Association of folic acid supplementation during pregnancy with the risk of autistic traits in children exposed to antiepileptic drugs in utero. JAMA Neurol..

[59] Figueiredo J.C., Grau M.V., Haile R.W., Sandler R.S., Summers R.W., Bresalier R.S., Burke C.A., McKeown-Eyssen G.E., Baron J.A. (2009). Folic acid and risk of prostate cancer: Results from a randomized clinical trial. J. Natl. Cancer Inst..

[60] Surén P., Roth C., Bresnahan M., Haugen M., Hornig M., Hirtz D., Lie K.K., Lipkin W.I., Magnus P., Reichborn-Kjennerud T. (2013). Association between maternal use of folic acid supplements and risk of autism spectrum disorders in children. JAMA.

[61] Levine S.Z., Kodesh A., Viktorin A., Smith L., Uher R., Reichenberg A., Sandin S. (2018). Association of maternal use of folic acid and multivitamin supplements in the periods before and during pregnancy with the risk of autism spectrum disorder in offspring. JAMA Psychiatry.

[62] DeVilbiss E.A., Magnusson C., Gardner R.M., Rai D., Newschaffer C.J., Lyall K., Dalman C., Lee B.K. (2017). Antenatal nutritional supplementation and autism spectrum disorders in the Stockholm Youth Cohort: Population based cohort study. BMJ.

[63] Strøm M., Granström C., Lyall K., Ascherio A., Olsen S.F. (2018). Research letter: Folic acid supplementation and intake of folate in pregnancy in relation to offspring risk of autism spectrum disorder. Psychol. Med..

[64] Virk J., Liew Z., Olsen J., Nohr E.A., Catov J.M., Ritz B. (2016). Preconceptional and prenatal supplementary folic acid and multivitamin intake and autism spectrum disorders. Autism.

[65] Sharman Moser S., Davidovitch M., Rotem R.S., Chodick G., Shalev V., Koren G. (2019). High dose folic acid during pregnancy and the risk of autism; the birth order bias: A nested case-control study. Reprod. Toxicol..

[66] Valera-Gran D., Navarrete-Muñoz E.M., Garcia de la Hera M., Fernández-Somoano A., Tardón A., Ibarluzea J., Balluerka N., Murcia M., González-Safont L., Romaguera D. (2017). Effect of maternal high dosages of folic acid supplements on neurocognitive development in children at 4–5 y of age: The prospective birth cohort Infancia y Medio Ambiente (INMA) study. Am. J. Clin. Nutr..

[67] Hirsch S., Sanchez H., Albala C., de la Maza M.P., Barrera G., Leiva L., Bunout D. (2009). Colon cancer in Chile before and after the start of the flour fortification program with folic acid. Eur. J. Gastroenterol. Hepatol..

[68] Mason J.B., Dickstein A., Jacques P.F., Haggarty P., Selhub J., Dallal G., Rosenberg I.H. (2007). A temporal association between folic acid fortification and an increase in colorectal cancer rates may be illuminating important biological principles: A hypothesis. Cancer Epidemiol. Biomark. Prev..

[69] Sterne J.A.C., Savović J., Page M.J., Elbers R.G., Blencowe N.S., Boutron I., Cates C.J., Cheng H.Y., Corbett M.S., Eldridge S.M. (2019). RoB 2: A revised tool for assessing risk of bias in randomized trials. BMJ.

[70] Sterne J.A., Hernán M.A., Reeves B.C., Savović J., Berkman N.D., Viswanathan M., Henry D., Altman D.G., Ansari M.T., Boutron I. (2016). ROBINS-I: A tool for assessing risk of bias in non-randomized studies of interventions. BMJ.

[71] Hooijmans C.R., Rovers M.M., de Vries R.B.M., Leenaars M., Ritskes-Hoitinga M., Langendam M.W. (2014). SYRCLE’s risk of bias tool for animal studies. BMC Med. Res. Methodol..

[72] Guyatt G.H., Oxman A.D., Vist G.E., Kunz R., Falck-Ytter Y., Alonso-Coello P., Schünemann H.J. (2008). GRADE: An emerging consensus on rating quality of evidence and strength of recommendations. BMJ.

[73] Fazili Z., Sternberg M.R., Paladugula N., Whitehead R.D., Chen H., Pfeiffer C.M. (2014). The loss of 5-methyltetrahydrofolate in human serum under suboptimal preanalytical conditions can only partially be recovered by an oxidation product. J. Nutr..

[74] Nelson B.C., Dalluge J.J., Margolis S.A. (2001). Preliminary application of liquid chromatography-electrospray-ionization mass spectrometry to the detection of 5-methyltetrahydrofolic acid monoglutamate in human plasma. J. Chromatogr. B Biomed. Sci. Appl..

[75] Pfeiffer C.M., Fazili Z., McCoy L., Zhang M., Gunter E.W. (2004). Determination of folate vitamers in human serum by stable-isotope-dilution tandem mass spectrometry and comparison with radioassay and microbiologic assay. Clin. Chem..

[76] Mills J.L., Molloy A.M. (2022). Lowering the risk of autism spectrum disorder with folic acid: Can there be too much of a good thing?. Am. J. Clin. Nutr..

[77] IntHout J., Ioannidis J.P., Borm G.F. (2014). The Hartung-Knapp-Sidik-Jonkman method for random effects meta-analysis is straightforward and considerably outperforms the standard DerSimonian-Laird method. BMC Med. Res. Methodol..

[78] Sidik K., Jonkman J.N. (2002). A simple confidence interval for meta-analysis. Stat. Med..

[79] DerSimonian R., Laird N. (1986). Meta-analysis in clinical trials. Control. Clin. Trials.

[80] Hartung J., Knapp G. (2001). On tests of the overall treatment effect in meta-analysis with normally distributed responses. Stat. Med..

[81] Hedges L.V., Olkin I. (1985). Statistical Methods for Meta-Analysis.

[82] Wan X., Wang W., Liu J., Tong T. (2014). Estimating the sample mean and standard deviation from the sample size, median, range and/or interquartile range. BMC Med. Res. Methodol..

[83] Luo D., Wan X., Liu J., Tong T. (2018). Optimally estimating the sample mean from the sample size, median, mid-range, and/or mid-quartile range. Stat. Methods Med. Res..

[84] Troesch B., Demmelmair J., Gimpfl M., Hecht C., Lakovic G., Roehle R., Sipka L., Trisic B., Vusurovic M., Schoop R. (2019). Suitability and safety of L-5-methyltetrahydrofolate as a folate source in infant formula: A randomized-controlled trial. PLoS ONE.

[85] Martinez-Morata I., Wu H., Galvez-Fernandez M., Ilievski V., Bottiglieri T., Niedzwiecki M.M., Goldsmith J., Jones D.P., Kioumourtzoglou M.-A., Pierce B. (2024). Metabolomic effects of folic acid supplementation in adults: Evidence from the FACT Trial. J. Nutr..

[86] Tam C., O’Connor D.L., Koren G. (2012). Circulating unmetabolized folic acid: Relationship to folate status and effect of supplementation. Obstet. Gynecol. Int..

[87] Cochrane K.M., Elango R., Devlin A.M., Hutcheon J.A., Karakochuk C.D. (2023). Human milk unmetabolized folic acid is increased following supplementation with synthetic folic acid as compared to (6S)-5-methyltetrahydrofolic acid. Sci. Rep..

[88] Egger M., Davey Smith G., Schneider M., Minder C. (1997). Bias in meta-analysis detected by a simple, graphical test. BMJ.

[89] Viechtbauer W. (2010). Conducting meta-analyses in R with the metafor package. J. Stat. Softw..

[90] Cochrane K.M., Elango R., Devlin A.M., Mayer C., Hutcheon J.A., Karakochuk C.D. (2024). Supplementation with (6S)-5-methyltetrahydrofolic acid appears as effective as folic acid in maintaining maternal folate status while reducing unmetabolized folic acid in maternal plasma: A randomized trial of pregnant women in Canada. Br. J. Nutr..

[91] Draicchio F., Hausser J., Sharafi M., Grier-Welch A., Vance A., Alamdari N., Futterman I.D., Chudnoff S., Malysheva O., Caudill M.A. (2026). Using 6S-5-methyltetrahydrofolate instead of folic acid in prenatal multivitamin reduces unmetabolized folic acid concentrations in the mother-fetus dyad: A 24-week randomized controlled trial. Front. Nutr..

[92] Obeid R., Rube E., Schön C., Geisel J. (2024). Serum concentrations of folate forms following supplementation of multimicronutrients with 400 µg or 800 µg mix of (6S)-5-methyltetrahydrofolate and folic acid (1:1) in women of childbearing age. Mol. Nutr. Food Res..

[93] Lamers Y., Prinz-Langenohl R., Brämswig S., Pietrzik K. (2006). Red blood cell folate concentrations increase more after supplementation with [6S]-5-methyltetrahydrofolate than with folic acid in women of childbearing age. Am. J. Clin. Nutr..

[94] Lamers Y., Prinz-Langenohl R., Moser R., Pietrzik K. (2004). Supplementation with [6S]-5-methyltetrahydrofolate or folic acid equally reduces plasma total homocysteine concentrations in healthy women. Am. J. Clin. Nutr..

[95] Rossi E., Beilby J.P., McQuillan B.M., Hung J. (1999). Biological variability and reference intervals for total plasma homocysteine. Ann. Clin. Biochem..

[96] van den Berg M., de Jong S.C., Devillé W., Rauwerda J.A., Jakobs C., Pals G., Boers G.H., Stehouwer C.D. (1999). Variability of fasting and post-methionine plasma homocysteine levels in normo- and hyperhomocysteinaemic individuals. Neth. J. Med..

[97] Prinz-Langenohl R., Bramswig S., Tobolski O., Smulders Y.M., Smith D.E.C., Finglas P.M., Pietrzik K. (2009). [6S]-5-methyltetrahydrofolate increases plasma folate more effectively than folic acid in women with the homozygous or wild-type 677C→T polymorphism of methylenetetrahydrofolate reductase. Br. J. Pharmacol..

[98] Fleming J.M., Rosa G., Bland V., Kauwell G.P.A., Malysheva O.V., Wettstein A., Hausman D.B., Bailey L.B., Park H.J. (2024). Response of one-carbon biomarkers in maternal and cord blood to folic acid dose during pregnancy. Nutrients.

[99] Miyaki K., Murata M., Kikuchi H., Takei I., Nakayama T., Watanabe K., Omae K. (2005). Assessment of tailor-made prevention of atherosclerosis with folic acid supplementation: Randomized, double-blind, placebo-controlled trials in each MTHFR C677T genotype. J. Hum. Genet..

[100] Huo Y., Li J., Qin X., Huang Y., Wang X., Gottesman R.F., Tang G., Wang B., Chen D., He M. (2015). Efficacy of folic acid therapy in primary prevention of stroke among adults with hypertension in China: The CSPPT randomized clinical trial. JAMA.

[101] Huang X., Qin X., Yang W., Liu L., Jiang C., Zhang X., Jiang S., Bao H., Su H., Li P. (2018). *MTHFR* gene and serum folate interaction on serum homocysteine lowering: Prospect for precision folic acid treatment. Arterioscler. Thromb. Vasc. Biol..

[102] Huang X., Bao H., Ding C., Li J., Cao T., Liu L., Wei Y., Zhou Z., Zhang N., Song Y. (2024). Optimal folic acid dosage in lowering homocysteine: Precision Folic Acid Trial to lower homocysteine (PFAT-Hcy). Eur. J. Nutr..

[103] Kalmbach R.D., Choumenkovitch S.F., Troen A.P., Jacques P.F., D’Agostino R., Selhub J. (2008). A 19-base pair deletion polymorphism in dihydrofolate reductase is associated with increased unmetabolized folic acid in plasma and decreased red blood cell folate. J. Nutr..

[104] Christensen K.E., Mikael L.G., Leung K.Y., Lévesque N., Deng L., Wu Q., Malysheva O.V., Best A., Caudill M.A., Greene N.D.E. (2015). High folic acid consumption leads to pseudo-MTHFR deficiency, altered lipid metabolism, and liver injury in mice. Am. J. Clin. Nutr..

[105] Christensen K.E., Hou W., Bahous R.H., Deng L., Malysheva O.V., Arning E., Bottiglieri T., Caudill M.A., Rozen R. (2016). Moderate folic acid supplementation and MTHFD1-synthetase deficiency in mice, a model for the R653Q variant, result in embryonic defects and abnormal placental development. Am. J. Clin. Nutr..

[106] Allegra C.J., Chabner B.A., Drake J.C., Lutz R., Rodbard D., Jolivet J. (1985). Enhanced inhibition of thymidylate synthase by methotrexate polyglutamates. J. Biol. Chem..

[107] Jolivet J., Jansen G., Peters G.J., Pinard M.F., Schornagel J.H. (1994). Leucovorin rescue of human cancer and bone marrow cells following edatrexate or methotrexate. Biochem. Pharmacol..

[108] Pietrzik K., Bailey L., Shane B. (2010). Folic acid and L-5-methyltetrahydrofolate: Comparison of clinical pharmacokinetics and pharmacodynamics. Clin. Pharmacokinet..

[109] Sweeney M.R., Staines A., Daly L., Traynor A., Daly S., Bailey S.W., Alverson P.B., Ayling J.E., Scott J.M. (2009). Persistent circulating unmetabolised folic acid in a setting of liberal voluntary folic acid fortification. Implications for further mandatory fortification?. BMC Public Health.

[110] Pentieva K., Selhub J., Paul L., Molloy A.M., McNulty B., Ward M., Marshall B., Dornan J., Reilly R., Parle-McDermott A. (2016). Evidence from a randomized trial that exposure to supplemental folic acid at recommended levels during pregnancy does not lead to increased unmetabolized folic acid concentrations in maternal or cord blood. J. Nutr..

[111] Obeid R., Kasoha M., Kirsch S.H., Munz W., Herrmann W. (2010). Concentrations of unmetabolized folic acid and primary folate forms in pregnant women at delivery and in umbilical cord blood. Am. J. Clin. Nutr..

[112] Obeid R., Herrmann W. (2006). Mechanisms of homocysteine neurotoxicity in neurodegenerative diseases with special reference to dementia. FEBS Lett..

[113] Matejcic M., de Batlle J., Ricci C., Biessy C., Perrier F., Yeap B.Y.S., Huybrechts I., Weiderpass E., Boutron-Ruault M., Cadeau C. (2017). Biomarkers of folate and vitamin B12 and breast cancer risk: Report from the EPIC cohort. Int. J. Cancer..

[114] Schmidt R.J., Tancredi D.J., Ozonoff S., Hansen R.L., Hartiala J., Allayee H., Schmidt L.C., Tassone F., Hertz-Picciotto I. (2012). Maternal periconceptional folic acid intake and risk of autism spectrum disorders and developmental delay in the CHARGE (CHildhood Autism Risks from Genetics and Environment) case-control study. Am. J. Clin. Nutr..

[115] Ghiasi M., Zwaigenbaum L., Moddemann D., White R.R., Dingwall-Harvey A.L.J., Grattan K.P., Murray M., Rybak N., Walker H., Lacaze-Masmonteil T. (2025). Follow-up of children born to mothers in Folic Acid Clinical Trial (FACT 4 Child): A prospective cohort study based on a double-blinded randomized controlled trial. BJOG.

[116] Wang M., Li K., Zhao D., Li L. (2017). The association between maternal use of folic acid supplements during pregnancy and risk of autism spectrum disorders in children: A meta-analysis. Mol. Autism.

[117] Iglesias Vázquez L., Canals J., Arija V. (2019). Review and meta-analysis found that prenatal folic acid was associated with a 58% reduction in autism but had no effect on mental and motor development. Acta Paediatr..

[118] Liu X., Zou M., Sun C., Wu L., Chen W.X. (2022). Prenatal folic acid supplements and offspring’s autism spectrum disorder: A meta-analysis and meta-regression. J. Autism Dev. Disord..

[119] Li M., Francis E., Hinkle S.N., Ajjarapu A.S., Zhang C. (2019). Preconception and prenatal nutrition and neurodevelopmental disorders: A systematic review and meta-analysis. Nutrients.

[120] Chen H., Qin L., Gao R., Jin X., Cheng K., Zhang S., Hu X., Xu W., Wang H. (2023). Neurodevelopmental effects of maternal folic acid supplementation: A systematic review and meta-analysis. Crit. Rev. Food Sci. Nutr..

[121] Pauwels S., Ghosh M., Duca R.C., Bekaert B., Freson K., Huybrechts I., Langie S.A.S., Koppen G., Devlieger R., Godderis L. (2017). Dietary and supplemental maternal methyl-group donor intake and cord blood DNA methylation. Epigenetics.

[122] Schmidt R.J., Kogan V., Shelton J.F., Delwiche L., Hansen R.L., Ozonoff S., Ma C.C., McCanlies E.C., Bennett D.H., Hertz-Picciotto I. (2017). Combined prenatal pesticide exposure and folic acid intake in relation to autism spectrum disorder. Environ. Health Perspect..

[123] Vollset S.E., Clarke R., Lewington S., Ebbing M., Halsey J., Lonn E., Armitage J., Manson J.E., Hankey G.J., Spence J.D. (2013). Effects of folic acid supplementation on overall and site-specific cancer incidence during the randomized trials: Meta-analyses of data on 50,000 individuals. Lancet.

[124] Ramaekers V.T., Blau N., Sequeira J.M., Nassogne M.C., Quadros E.V. (2007). Folate receptor autoimmunity and cerebral folate deficiency in low-functioning autism with neurological deficits. Neuropediatrics.

[125] Sequeira J.M., Ramaekers V.T., Quadros E.V. (2013). The diagnostic utility of folate receptor autoantibodies in blood. Clin. Chem. Lab. Med..

[126] Frye R.E., Rossignol D.A. (2014). Treatments for biomedical abnormalities associated with autism spectrum disorder. Front. Pediatr..

[127] Obeid R., Kirsch S.H., Dilmann S., Klein C., Eckert R., Geisel J., Herrmann W. (2016). Folic acid causes higher prevalence of detectable unmetabolized folic acid in serum than B-complex: A randomized trial. Eur. J. Nutr..

[128] Obeid R., Kirsch S.H., Kasoha M., Eckert R., Herrmann W. (2011). Concentrations of unmetabolized folic acid and primary folate forms in plasma after folic acid treatment in older adults. Metabolism.

[129] (2006). The Heart Outcomes Prevention Evaluation (HOPE) 2 Investigators. Homocysteine lowering with folic acid and B vitamins in vascular disease. N. Engl. J. Med..

[130] VITATOPS Trial Study Group (2010). B vitamins in patients with recent transient ischaemic attack or stroke in the VITAmins to Prevent Stroke (VITATOPS) trial: A randomised, double-blind, parallel, placebo-controlled trial. Lancet Neurol..

[131] Toole J.F., Malinow M.R., Chambless L.E., Spence J.D., Pettigrew L.C., Howard V.J., Sides E.G., Wang C.H., Stampfer M. (2004). Lowering homocysteine in patients with ischemic stroke to prevent recurrent stroke, myocardial infarction, and death: The Vitamin Intervention for Stroke Prevention (VISP) randomized controlled trial. JAMA.

[132] Durga J., van Boxtel M.P., Schouten E.G., Kok F.J., Jolles J., Katan M.B., Verhoef P. (2007). Effect of 3-year folic acid supplementation on cognitive function in older adults in the FACIT trial: A randomised, double blind, controlled trial. Lancet.

[133] de Jager C.A., Oulhaj A., Jacoby R., Refsum H., Smith A.D. (2012). Cognitive and clinical outcomes of homocysteine-lowering B-vitamin treatment in mild cognitive impairment: A randomized controlled trial. Int. J. Geriatr. Psychiatry.

[134] Centers for Disease Control and Prevention Folic Acid Safety, Interactions, and Health Outcomes. https://www.cdc.gov/folic-acid/about/safety.html.

[135] Zhao R., Aluri S., Goldman I.D. (2017). The proton-coupled folate transporter (PCFT-SLC46A1) and the syndrome of systemic and cerebral folate deficiency of infancy: Hereditary folate malabsorption. Mol. Asp. Med..

[136] US Food and Drug Administration FDA Approves First Treatment for Patients with Cerebral Folate Transport Deficiency. FDA News Release, 10 March 2026. https://www.fda.gov/news-events/press-announcements/fda-approves-first-treatment-patients-cerebral-folate-transport-deficiency.

[137] Turck D., Bohn T., Castenmiller J., de Henauw S., Hirsch-Ernst K.I., Knutsen H.K., Maciuk A., Mangelsdorf I., McArdle H.J., EFSA Panel on Nutrition, Novel Foods and Food Allergens (NDA) (2023). Scientific opinion on the tolerable upper intake level for folate. EFSA J..

[138] Selhub J., Morris M.S., Jacques P.F. (2007). In vitamin B12 deficiency, higher serum folate is associated with increased total homocysteine and methylmalonic acid concentrations. Proc. Natl. Acad. Sci. USA.

[139] Moore E.M., Ames D., Mander A.G., Carne R.P., Brodaty H., Woodward M.C., Boundy K., Ellis K.A., Bush A.I., Faux N.G. (2014). Among vitamin B12 deficient older people, high folate levels are associated with worse cognitive function: Combined data from three cohorts. J. Alzheimer’s Dis..

[140] Brieger K.K., Bakulski K.M., Pearce C.L., Baylin A., Dou J.F., Feinberg J.I., Croen L.A., Hertz-Picciotto I., Newschaffer C.J., Fallin M.D. (2022). The association of prenatal vitamins and folic acid supplement intake with odds of autism spectrum disorder in a high-risk sibling cohort, the Early Autism Risk Longitudinal Investigation (EARLI). J. Autism Dev. Disord..

[141] Wiens D., DeSoto M.C. (2017). Is high folic acid intake a risk factor for autism?—A review. Brain Sci..

[142] Koren G., Sharman Moser S. (2019). Does high-dose gestational folic acid increase the risk for autism? The birth order hypothesis. Med. Hypotheses.

[143] National Institutes of Health, Office of Dietary Supplements Vitamin B12: Fact Sheet for Health Professionals. https://ods.od.nih.gov/factsheets/VitaminB12-HealthProfessional/.

[144] Homocysteine Studies Collaboration (2002). Homocysteine and risk of ischemic heart disease and stroke: A meta-analysis. JAMA.

[145] Wald D.S., Law M., Morris J.K. (2002). Homocysteine and cardiovascular disease: Evidence on causality from a meta-analysis. BMJ.

[146] Clarke R., Halsey J., Lewington S., Lonn E., Armitage J., Manson J.E., Bønaa K.H., Spence J.D., Nygård O., Jamison R. (2010). Effects of lowering homocysteine levels with B vitamins on cardiovascular disease, cancer, and cause-specific mortality: Meta-analysis of 8 randomized trials involving 37,485 individuals. Arch. Intern. Med..

[147] National Institutes of Health, Office of Dietary Supplements Folate: Fact Sheet for Health Professionals. https://ods.od.nih.gov/factsheets/Folate-HealthProfessional/.

[148] Ramaekers V.T., Rothenberg S.P., Sequeira J.M., Opladen T., Blau N., Quadros E.V., Selhub J. (2005). Autoantibodies to folate receptors in the cerebral folate deficiency syndrome. N. Engl. J. Med..

[149] Bobrowski-Khoury N., Ramaekers V.T., Sequeira J.M., Quadros E.V. (2021). Folate receptor alpha autoantibodies in autism spectrum disorders: Diagnosis, treatment, and prevention. J. Pers. Med..

[150] Giorlandino C., Margiotti K., Fabiani M., Mesoraca A. (2025). Folinic acid supplementation during pregnancy in two women with folate receptor alpha autoantibodies: Potential prevention of autism spectrum disorder in offspring. Clin. Transl. Neurosci..

[151] Giorlandino C., Margiotti K., Fabiani M., Mesoraca A., D’Emidio L., Raffio R., Coco C., Mastrandrea M.L., Pasquale C., Cupellaro M. (2025). Maternal folate receptor alpha autoantibodies and increased fetal nuchal translucency as potential early markers of autism spectrum disorder. Brain Behav..

[152] Giorlandino C., Mesoraca A., Margiotti K., Fabiani M., Cupellaro M., Giorlandino F., Mastrandrea M.L., Raffio R., Pignataro F., Mangiafico L. (2026). Folinic acid supplementation in folate receptor alpha autoantibodies-positive pregnancy: A pilot randomized study on neurodevelopmental outcomes. Reprod. Female. Child Health.

[153] Clément A., Menezo Y., Cohen M., Cornet D., Clément P. (2020). 5-Methyltetrahydrofolate reduces blood homocysteine level significantly in C677T methyltetrahydrofolate reductase single-nucleotide polymorphism carriers consulting for infertility. J. Gynecol. Obstet. Hum. Reprod..

[154] Smith A.D., Kim Y.I., Refsum H. (2008). Is folic acid good for everyone?. Am. J. Clin. Nutr..

[155] Houghton L.A., Sherwood K.L., Pawlosky R., Ito S., O’Connor D.L. (2006). [6S]-5-Methyltetrahydrofolate is at least as effective as folic acid in preventing a decline in blood folate concentrations during lactation. Am. J. Clin. Nutr..

[156] Higgins J.P.T., Thompson S.G., Deeks J.J., Altman D.G. (2003). Measuring inconsistency in meta-analyses. BMJ.

[157] Maruvada P., Stover P.J., Mason J.B., Bailey R.L., Davis C.D., Field M.S., Finnell R.H., Garza C., Green R., Gueant J.L. (2020). Knowledge gaps in understanding the metabolic and clinical effects of excess folates/folic acid: A summary, and perspectives, from an NIH workshop. Am. J. Clin. Nutr..

[158] Cochran W.G. (1954). The combination of estimates from different experiments. Biometrics.

