# Supplementary material for: Unmetabolized Folic Acid: Biology, Epidemiology, and Clinical Consequences: A Systematic Review
Source: Nutrients. 2026 Sep 3;18(17):2887. doi: 10.3390/nu18172887 (PMC13567015; doi:10.3390/nu18172887)
Supplement: Supplementary file 1 [file nutrients-18-02887-s001.zip › Supplementary Methods (revised).pdf]

Supplementary Methods

Quantitative synthesis procedures, study-level data handling, and search strategy.

1. Effect-size construction.

Continuous-outcome pools. For each continuous-outcome pool, Hedges' g [157] standardized mean differences (SMD) were computed from arm-level means, standard deviations, and group sizes using the conventional pooled standard deviation. The small-sample correction $J = 1 - 3 / (4(n_1 + n_2) - 9)$ was applied to each g. Crossover trials with paired-comparison summary statistics were converted to between-arm SMD by treating the paired within-individual SD as the relevant pooled SD; where only paired-difference SD was reported, an imputed correlation of 0.5 was applied (sensitivity analyses with $\varrho = 0.3$ and $\varrho = 0.7$ preserved direction and magnitude). For Pool P3 (plasma total homocysteine, uniformly assayed across trials and clinically interpretable on its native scale), raw between-arm mean differences in μmol/L were the planned effect measure. No pooled estimate is reported because the retained reports do not provide recoverable per-arm variances in a common arithmetic mean ± SD form and include important design limitations.

Cord-UMFA × ASD synthesis. The cord-blood UMFA and autism spectrum disorder evidence was retained as a narrative single-cohort synthesis because only the Boston Birth Cohort, in the retrieved cohort evidence, provided a comparable highest-versus-lowest cord-UMFA category estimate among the retrieved cohort evidence. No log-OR meta-analysis was performed.

2. Conversion of reported summary statistics.

Harmonization to mean ± SD. Continuous outcomes were harmonized to mean and standard deviation (SD) before pooling. Three conversion procedures were used as required by the format reported in the primary publication.

(a) Median with interquartile range (Wan 2014 [77], Method 3). Mean was estimated as (Q1 + median + Q3)/3 and SD as $(Q3 - Q1) / [2 \cdot \Phi^{-1}((0.75n - 0.125)/(n + 0.25))]$, where Φ^{-1} is the inverse standard normal CDF [77].

(b) Median with full range (Wan 2014 [77], Method 1). Mean was estimated as (min + 2 · median + max)/4 and SD as $(\max - \min) / [2 \cdot \Phi^{-1}((n - 0.375)/(n + 0.25))]$.

Application to Pool P1 UMFA inputs. The Troesch 2019 [82] and Cochrane 2024 [83] Pool P1 inputs were reported as medians with interquartile ranges and were converted to mean ± SD with Wan et al. Method 3, applying the $\eta(n)$ denominator given above rather than the asymptotic constant 1.35, before calculation of Hedges' g. Wan et al. Method 1 was the prespecified conversion for any median-with-full-range input; it was not used for either of these Pool P1 inputs because neither supplied a full range. The resulting Pool P1 SMDs are therefore conversion-based approximations.

(c) Mean with standard error of the mean. SD was recovered as $SE \cdot \sqrt{n}$ (Luo et al. 2018 [78]).

Mass-to-molar conversion. Plasma UMFA concentrations reported in mass units (ng/mL) were converted to molar units (nmol/L) using the synthetic folic acid molecular weight of 441.4 g/mol (ng/mL × 2.265 = nmol/L).

Pool S3 per-trial conversion mapping. Pool S3 inputs were derived as follows: Murphy/FACT 2021 [43] (median + IQR → Method 3); Tam 2012 [80] Week-12 values (median + range → Method 1); Fleming 2024 [86] (mean ± SEM at delivery → Luo 2018 [78]; ng/mL × 2.265 → nmol/L).

3. Random-effects pooling and small-sample CI adjustment.

Pooling estimator. All retained meta-analyses used a DerSimonian–Laird (DL) random-effects estimator [152] for the between-study variance τ^2. The pooled point estimate is the inverse-variance-weighted mean of the constituent effect sizes under the random-effects model.

Hartung–Knapp–Sidik–Jonkman (HKSJ) small-sample variance adjustment. To address the well-documented anticonservatism of conventional Wald-type confidence intervals when $k \leq 10$, the HKSJ adjustment was applied uniformly to every pool [75,76,153]. Under HKSJ, the variance of the pooled estimate is $SE^2 = \Sigma w_i(y_i - \mu)^2 / [(k-1) \cdot \Sigma w_i]$ with $w_i = 1/(v_i + \tau^2)$, and the 95% confidence interval is constructed using the t-distribution with df = k − 1. The Sidik–Jonkman t-distribution interval [76] was retained because it improves coverage probability relative to the conventional DerSimonian–Laird normal interval, especially when the number of studies is small or moderate, while requiring no iterative computation. The HKSJ approach is recommended by recent methodological consensus for systematic reviews containing small numbers of trials and is implemented identically to the R metafor package [159]'s method.tau = "DL", test = "knha" configuration.

Behavior at k = 2 and dual-CI reporting. For the exploratory Pool S3 sensitivity analysis with k = 2 the t-distribution has df = 1 and the resulting HKSJ confidence interval is markedly wide; this is reported transparently in the Pool S3 sensitivity analysis (Supplementary Figure S3b), and the corresponding DL normal-CI ($\mu \pm 1.96 \cdot \sqrt{(1/\Sigma w_i)}$) is provided alongside the HKSJ interval to aid interpretation.

95% prediction intervals. A single rule applies throughout: prediction intervals are computed only for pools with $k \geq 5$, using a t-distribution with k − 2 degrees of freedom and variance $\tau^2 + SE^2$(pooled). No pool in this review reaches k = 5; therefore no prediction interval is reported anywhere, including the Pool S3 k = 2 sensitivity analysis.

4. Heterogeneity quantification.

Statistics reported. Between-study heterogeneity was quantified by Cochran's Q [158] (with χ^2 p-value), τ^2 (between-study variance on the effect-size scale), and I^2 (proportion of total variance attributable to between-study heterogeneity). I^2 was interpreted using the conventional Higgins thresholds [154] (25 / 50 / 75% as low / moderate / high).

Pre-specified sources. Pre-specified sources of heterogeneity were population, exposure dose, assay generation, trial duration, and risk-of-bias domain.

5. Protocol deviations and post-hoc analyses.

All departures from the PROSPERO-registered protocol (CRD420261407616) are consolidated here rather than distributed across the Methods, so that the registered analysis plan and the as-executed analysis can be compared in one place. Each deviation is stated with its rationale and its consequence for the reported estimates. No deviation altered the direction of any pooled estimate for the registered folic acid (FA) versus (6S)-5-methyltetrahydrofolate PICO question, and none altered the GRADE certainty ratings, which are determined by Pools P1–P3 alone.

D1. Post-registration stratification of observational evidence into four exposure classes. The registered protocol admitted observational studies reporting "any biological or clinical endpoint plausibly attributable to UMFA," which in practice admitted studies of FA intake, of fortification, and of total circulating folate alongside studies of directly measured UMFA. Because that sampling frame is defensible for a contextual literature but is not a valid basis for causal attribution to UMFA, the observational evidence was stratified post hoc into four mutually exclusive exposure classes (main manuscript Table 1), and conclusions about UMFA specifically are drawn only from Class 1 (directly measured UMFA). Consequence: no change to any pooled estimate; the stratification constrains interpretation rather than the analysis.

D2. Removal of the Schön/Obeid 2024 [85] entry from Pool P1. The entry was removed after source-anchored re-extraction established that the publication evaluates a 1:1 FA/(6S)-5-MTHF mixture rather than a head-to-head contrast of the two folate forms, and therefore does not meet the registered Comparator criterion. Consequence: Pool P1 is reported at k = 3 rather than k = 4.

D3. Duplicate-cohort removal from Pool S3. Each candidate publication was cross-checked against ClinicalTrials.gov registration numbers and against authors' explicit statements about cohort overlap. When two or more publications reported overlapping outcomes from the same underlying trial cohort, only the index publication of that cohort was retained in any given pool. A secondary 2024 FACT metabolomics publication reported the FACT trial cohort (NCT01355159) already represented by Murphy et al. 2021 [43] in Pool S3 and was therefore removed. Consequence: the recomputed k = 3 estimate is the one reported in Supplementary Figure S3 and Supplementary Table S7.

D4. Post-hoc k = 2 sensitivity analysis for Pool S3 (Tam 2012 [80] excluded). Following source-anchored re-extraction it was observed that the Wan-Method-1 standard-deviation estimator applied to Tam et al. 2012 [80] is dominated by a single 86.4 nmol/L low-dose outlier, and that the primary Tam publication reports z = −0.11, P = 0.91 for the between-dose UMFA comparison at Week 12. A sensitivity analysis excluding Tam et al. 2012 [80] (k = 2) was therefore performed and is shown in Supplementary Figure S3b. This analysis was not pre-specified and is reported as exploratory. Consequence: the pooled mean difference moves from −1.2 nmol/L to +4.6 nmol/L, both directionally null with confidence intervals spanning zero; the GRADE certainty rating for the registered question is unaffected because it is determined by Pools P1–P3.

D5. Cord-blood UMFA × autism spectrum disorder evidence retained as a narrative single-cohort synthesis. Only the Boston Birth Cohort, among the retrieved cohort evidence, provided a comparable highest-versus-lowest cord-UMFA category estimate, so the registered intention to pool this outcome could not be executed. Consequence: no log-odds-ratio meta-analysis was performed; the evidence is presented descriptively in Supplementary Table S6 and Supplementary Figure S2.

D6. Egger's test [155] and funnel plots not performed. Egger's test was pre-specified for pools with k ≥ 10 studies. No pool reached that threshold, so the test was not performed, and funnel plots were not produced because no pool contained a sufficient number of studies for a funnel plot to be interpretable. Consequence: publication bias could not be formally assessed in any pool, and this is recorded as a limitation rather than as a GRADE downgrade beyond the imprecision downgrades already applied.

D7. Imputations not specified in the registered protocol. Two imputations were required by the reporting formats of the primary publications. (i) For crossover trials reporting only paired-difference standard deviations, a within-individual correlation of ρ = 0.5 was imputed; sensitivity analyses at ρ = 0.3 and ρ = 0.7

preserved the direction and magnitude of every affected estimate. (ii) Where a standard deviation was reported for the FA arm only, the (6S)-5-MTHF arm standard deviation was imputed from the FA arm on the same outcome, a conservative assumption that biases the pooled estimate toward zero. Consequence: no reversal of direction in any affected pool.

D8. Scope of formal GRADE rating. GRADE, structured PICO eligibility, and pre-specified outcome definitions were applied to the registered question only — whether substitution of (6S)-5-MTHF for FA alters plasma UMFA, erythrocyte folate, or plasma total homocysteine — because GRADE addresses certainty about a specific question. Mechanistic, observational, and exploratory-pool evidence is appraised descriptively with per-study risk-of-bias and OCEBM 2011 [42] judgements and carries no aggregate certainty rating. Consequence: Supplementary Table S8 is the only table in this package that reports a formal GRADE certainty rating.

D9. Declared post-registration amendment for Draicchio 2026 [84] in Pool P1. Draicchio 2026 [84] was retained in Pool P1 only despite a non-equimolar multi-nutrient comparison (approximately 588 μg (6S)-5-MTHF versus 800 μg FA, with approximately 40% more folate on a molar basis in the FA arm; other B vitamins, choline, and vitamin D also differed) and per-protocol-only analysis. Consequence: the amendment is declared in Supplementary Table S4 and is accompanied by the prespecified-criterion equimolar-only sensitivity analysis excluding Draicchio (the protocol prespecified leave-one-out sensitivity analysis as an analysis type and prespecified the equimolar-dose criterion; its application to this trial follows from the amendment); the trial contributes to neither Pool P2 nor Pool P3 because it measured neither erythrocyte folate nor plasma tHcy.

D10. Prediction-interval rule not specified at registration. The registered protocol did not state a minimum pool size for prediction intervals. A single rule was adopted during analysis and applied throughout: prediction intervals are computed only for pools with $k \geq 5$, and for smaller pools they are neither reported nor used in any inference. This rule is stated in Section 2.8 of the main text as an implemented analysis rule and is not characterized as prespecified. Consequence: no pool in this review reached $k = 5$, so no prediction interval is reported anywhere and no statement in this review rests on one.

D11. Rule for withholding a pooled estimate, not specified at registration. The registered protocol did not state conditions under which a pooled estimate would be withheld. Two conditions were adopted during analysis and are stated in Section 2.8: where only two studies contribute and they are directionally discordant, because a two-study Hartung–Knapp–Sidik–Jonkman interval is not informative; and where none of the contributing reports supplies the per-arm arithmetic means and standard deviations that the specified effect metric requires, because any pooled value would rest on assumptions about distributional form and variance that the sources do not support. Consequence: no pooled estimate is reported for Pool P2 (erythrocyte folate, first condition) or for Pool P3 (plasma total homocysteine, second condition); both outcomes are reported study by study, and the GRADE certainty for each is very low.

D12. Replacement of the equal-allocation assumption with recovered per-arm denominators. The originally submitted analysis assumed equal allocation between arms where a report gave only a pooled analysed n. During source-anchored re-extraction the per-arm analysed denominators were recovered from internal evidence in the source publications, as set out in Section 7, and the equal-allocation assumption was removed from the analysis entirely. Consequence: the Troesch 2019 [82] erythrocyte-folate interval widens from +0.40 [+0.14, +0.65] to +0.40 [+0.08, +0.73]; the Cochrane 2024 [83] erythrocyte-folate estimate is unchanged; and the

Troesch 2019 [82] plasma-UMFA contribution to Pool P1 changes from +1.15 [+0.87, +1.43] to +1.33 [+0.84, +1.82], which lowers the precision of the Pool P1 pooled estimate and of its equimolar-only sensitivity analysis. No direction of effect is reversed. The plasma-UMFA figure quoted here was itself subsequently corrected; see D13.

D13. Correction of the Wan Method 3 effect-size derivation for the two median-reported Pool P1 trials. Section 1 of these Supplementary Methods specifies that medians with interquartile ranges are converted using mean = (Q1 + median + Q3)/3 and SD = (Q3 − Q1)/η(n), with $\eta(n) = 2\cdot\Phi^{-1}((0.75n - 0.125)/(n + 0.25))$. Re-verification in response to peer review established that the originally submitted Pool P1 estimates had not been computed that way: the median had been substituted directly for the mean, and the standard deviation had been approximated as IQR/1.35, the asymptotic value of η(n), rather than the sample-size-dependent η(n). Both median-reported trials were re-derived using the specified estimator. Consequence: Troesch 2019 [82] changes from +1.33 [+0.84, +1.82] to +1.12 [+0.64, +1.60], and Cochrane 2024 [83] from +1.05 [+0.50, +1.60] to +1.26 [+0.67, +1.84] (the latter also incorporating the corrected endline denominator of 25 / 29 in place of the randomized 30 / 30). The two trial estimates move closer together, so Pool P1 heterogeneity falls from $I^2 = 46\%$ to $I^2 = 30\%$ and τ^2 from 0.058 to 0.031; the pooled estimate is essentially unchanged, from +1.01 [+0.14, +1.89] to +0.99 [+0.20, +1.79]; and the prespecified-criterion equimolar-only sensitivity analysis changes from +1.21 [−0.56, +2.97], $p = 0.073$ to +1.17 [+0.32, +2.03], $p = 0.036$, so that it now excludes the null. No direction of effect is reversed. The GRADE imprecision downgrade for Pool P1 was re-derived because its originally stated rationale cited the null-inclusive equimolar-only interval; the downgrade is retained on the grounds of interval width and the conditional lower bound, and the certainty rating remains low.

D14. Addition of allocation and censoring sensitivity analyses for Troesch 2019 [82]. The registered protocol did not anticipate that a contributing trial would report a pooled analysed denominator without an arm-specific breakdown. Because the per-arm split of the 124 quantifiable plasma-UMFA observations in Troesch 2019 [82] is not stated in the source and is therefore assumed rather than recovered, two post-registration sensitivity analyses were added in revision and are reported as Supplementary Table S4b: Panel A varies the assumed folic acid / L-5-MTHF allocation across its full plausible range, and Panel B quantifies the direction of bias introduced by limit-of-quantification censoring by simulation. Consequence: Pool P1 varies only between +0.96 and +1.00 across all allocations tested, with every interval excluding the null, and LOQ censoring is shown to bias the standardized mean difference toward the null by approximately 0.15 to 0.18, so the reported trial estimate is conservative with respect to that censoring. Neither analysis alters a registered conclusion; both are declared here as post-registration additions and are not characterized as prespecified.

D15. Correction of the GRADE arithmetic for Pool P2 (erythrocyte folate). The originally submitted Supplementary Table S8 recorded one downgrade for serious inconsistency and one for serious imprecision, then reported the resulting certainty as very low. A randomized body of evidence begins at high certainty, so two single-level downgrades yield low, not very low; the stated rating did not follow from the stated domain judgements. On re-examination the imprecision for this outcome is very serious rather than serious, on four grounds that were already documented but had not been carried into the domain rating: no pooled estimate is derivable at all, the two contributing trial intervals point in opposite directions, the combined analysed sample is 204 participants, and no non-inferiority margin was prespecified. The imprecision downgrade is therefore recorded as two levels, giving three downgrades in total and very-low certainty. The published rating is unchanged; the derivation that supports it has been corrected. No pooled estimate, effect size, or conclusion for Pool P2 is affected.

D16. Reconstruction of Pool S4 (MTHFR 677C→T genotype × folic acid on plasma total homocysteine) and withdrawal of its previously reported pooled estimates. The originally submitted Pool S4 reported pooled genotype-specific mean differences of −0.99 μmol/L [−1.42, −0.56] for CC, −1.26 [−1.61, −0.91] for CT and −1.90 [−2.41, −1.39] for TT, together with a linear trend of −0.46 μmol/L per 677T allele, and attributed these to three cited reports. Source re-verification undertaken in response to peer review established that two of the three cited reports do not measure the pooled outcome: the primary report of the large hypertension trial reports genotype-stratified serum folate but not genotype-stratified change in total homocysteine, and the remaining report specifies change in S-adenosylhomocysteine as its outcome with total homocysteine entering only as a baseline characteristic and model covariate. The pool was therefore not a three-study pool of the stated outcome, and the reported pooled values and per-allele trend could not be reproduced from the cited sources. Those values and that trend have been withdrawn in full and are not reported anywhere in this revision. Pool S4 has been rebuilt from the three randomized reports that do report the outcome, each trial entered once, and is now presented without a pooled estimate under the same rule applied to Pools P2 and P3 in deviation D11, because the contributing trials differ in population, dose and duration to a degree that makes a single pooled estimate uninterpretable. Full provenance for every retained value, including the derivation of the placebo-corrected differences and the reasons one estimate is reported but flagged as not interpretable at face value, is given in the footnote to Supplementary Table S7. This deviation was not pre-specified and arose entirely from post-registration source verification.

D17. Complete source re-verification of the evidence ledger and re-derivation of all study counts. The originally submitted Supplementary Tables S1–S3 contained 101 analytic contributions whose design and key-finding cells had been entered without a second reader confirming each cell against the publication itself. In response to peer review, every one of the 101 rows was re-checked directly against the source: the record was located by author, title, DOI and PubMed identifier; the design, population, exposure, comparator, outcome and reported result were re-read from the publication; and each ledger cell was compared with what the publication states. Twenty-one contributions were confirmed exactly as submitted. Forty-eight were retained with one or more cells corrected. The corrections fall into recurring types, each identified per row in Supplementary Table S10: reversal or contradiction of the submitted direction or statistical significance; misidentification of the study population or named cohort; an incorrect publication year; an incorrect stated intervention or dose; a design label describing a study as randomized, crossover or controlled when the source states otherwise; attribution of a finding to a study that did not measure the relevant analyte; and a risk-of-bias judgement inconsistent with the design as reported. One contribution was reclassified into the interventional table because the cited publication reports a randomized allocation of folic acid. Thirty-one contributions were withdrawn: three because the publication predates the 1 January 1995 eligibility window, eight because the cited publication is a review, meta-analysis, or other secondary synthesis rather than a primary study, eight because no publication matching the cited reference could be retrieved by author, title, DOI or topic search, and twelve because the cited publication's verified content did not support a distinct contribution in that evidence stream, with the publication's genuine contribution retained elsewhere in the ledger. The withdrawn rows for which no publication could be located are reported as unresolved rather than replaced; no substitute source was adopted in order to preserve a previously submitted claim. Six reference-list entries were corrected in the same pass, and one reference cited in the ledger but absent from the reference list was added. Original row identifiers are preserved and are not reused, and the row-level disposition of all 101 originally submitted contributions is given in Supplementary Table S10. All study counts were then re-derived from the rebuilt ledger rather than adjusted arithmetically: the review now includes 65 unique publications describing 61

unique primary studies and generating 71 analytic contributions (15 mechanistic, 24 observational, 32 interventional), of which 51 studies contribute human participant data. No effect-size input to the registered quantitative synthesis changed. Of the rows contributing to Pools P1–P3, only two were altered: the publication year of I10 was corrected from 2023 to 2024, and Houghton 2006 [88] was found ineligible for Pool P3 (deviation D18). Every pooled and unpooled estimate reported for Pools P1–P3 therefore stands as computed in deviations D12 to D15. This deviation was not pre-specified and arose entirely from post-registration source verification.

D18. Exclusion of Houghton 2006 [88] from Pool P3 (plasma total homocysteine). The originally submitted Pool P3 listed three contributing reports, one of which was Houghton et al. 2006 [88]. Source re-verification established that only the (6S)-5-methyltetrahydrofolate versus placebo contrast in that trial was randomized; the folic acid arm was a separately assigned reference group. The folate-form contrast on which Pool P3 depends is therefore not a randomized comparison and does not satisfy the registered eligibility criterion of a head-to-head randomized trial of folate forms. Houghton 2006 [88] has been removed from the pool and is retained in Supplementary Table S5 as an excluded contrast with the reason stated, reducing Pool P3 to two eligible reports and the registered synthesis as a whole to four distinct trial cohorts. This exclusion does not change the qualitative conclusion for plasma total homocysteine, because neither remaining report supplies the per-arm arithmetic means and standard deviations that a mean-difference synthesis requires, and pooling of that outcome had already been abandoned under deviation D11. This deviation was not pre-specified and arose entirely from post-registration source verification. D19. Withdrawal of the pooled secondary synthesis previously counted as an interventional contribution. During re-verification the entry originally numbered O32 was found to be Vollset et al. 2013 [109], a pooled analysis of thirteen randomized folic acid trials, and was initially moved into the interventional table as a declared exception to the secondary-synthesis exclusion on the grounds that it constitutes the largest randomized safety dataset bearing on folic acid exposure. That exception was inconsistent with the eligibility criterion stated in Section 2.2 of the main text and with the unit-of-analysis rule stated in Section 2.4, both of which exclude reviews, meta-analyses, and other secondary syntheses from the count of included reports, studies, and analytic contributions without qualification. Rather than amend the eligibility criteria after the fact to accommodate a single record, the entry has been withdrawn from the evidence ledger and is retained only as a contextual citation where the randomized cancer-safety evidence is discussed. Its largest component trial remains in the ledger in its own right as I32 (Figueiredo 2009 [57]). Consequence: the interventional stream falls from 33 to 32 analytic contributions and the ledger total from 72 to 71; unique publications fall from 66 to 65 and full-text exclusions rise from 144 to 145, so that 210 reports assessed at full text yield 65 included publications. The count of unique primary studies is unchanged at 61, because the withdrawn record's underlying trial is already represented by I32, and the counts of studies contributing human participant data (51) and non-participant units (10) are likewise unchanged. No pool, effect-size input, risk-of-bias judgement, or GRADE rating is affected, because the withdrawn record contributed to no pool. This deviation was not pre-specified and arose from the consistency review carried out before resubmission.

Pre-specified analyses are described in Section 6. Deviations D1, D2, D4, D7(i), D9, D12, and D13 arose during source-anchored re-extraction or re-verification after the protocol was registered; D3, D5, D6, and D8 arose because the retrieved evidence base did not support an analysis the protocol had anticipated; D10 and D11 are analysis rules that the registered protocol did not address and that were adopted during analysis, stated in Section 2.8 of the main text and not characterized as prespecified; D14 comprises post-registration sensitivity

analyses added in revision in response to peer review; D15 is a correction, made in revision, to the recorded derivation of an existing certainty rating; D16 is a withdrawal and reconstruction, made in revision, of an exploratory pool whose previously reported estimates could not be reproduced from their cited sources; D17 and D18 arose from the complete source re-verification of the evidence ledger carried out in revision; and D19 is a withdrawal, made before resubmission, of a record whose retention had been inconsistent with the registered eligibility criteria.

6. Pre-specified sensitivity analyses.

These sensitivity analyses were pre-specified in the PROSPERO-registered protocol (CRD420261407616) for the focused FA-versus-(6S)-5-MTHF quantitative-synthesis component of this systematic review. For every retained pool we computed: (i) leave-one-out re-estimation (each trial omitted in turn); and (ii) restriction to trials using LC-MS/MS UMFA assay where applicable. None of these sensitivity analyses reversed direction or moved the central estimate outside its primary 95% HKSJ CI.

7. Study-data extraction rules.

Reviewer-level extraction. For each included trial, two reviewers independently extracted arm-level mean, SD, n, and (where reported) baseline-adjusted estimates. For pre–post designs we used the per-arm endpoint mean and SD; for crossover designs we used the within-individual difference. Discordant extractions were reconciled by consensus; unresolved discrepancies were adjudicated by a third reviewer.

Per-arm analysed n for Pools P1 and P2. Where a trial reported only a pooled analysed n, per-arm denominators were derived from internal evidence in the source publication wherever that evidence determines them uniquely. No equal-allocation assumption is used anywhere in the final analysis. One denominator is not uniquely determined by any reported quantity and remains an explicit assumption: the arm-specific split of the 124 quantifiable plasma-UMFA observations in Troesch 2019 [82], described below and subjected to sensitivity analysis in Supplementary Table S4b. The distinction is maintained throughout: a derived denominator is the only value consistent with the reported data, whereas an assumed denominator is one plausible value among several.

For **Troesch 2019 [82]** the per-protocol group sizes are not stated numerically. They were recovered by back-calculation from the reported MTHFR C677T genotype percentages against the stated analysed total of n = 238: a complete integer search over all group-size triples summing to 238 returned a single solution consistent with every reported percentage, namely 69 (L-5-MTHF), 81 (folic acid), and 88 (breastfed reference). The derivation was cross-checked against the reported between-group $p = 0.0095$ for erythrocyte folate, which is consistent with approximately 150 participants across the two randomized arms and is not consistent with the 238 previously assumed. Because the reported age and sex distribution corresponds to n = 244, the same 69:81:88 ratio was applied to that total, giving 71 / 83 / 90. Plasma UMFA at visit 4 was analysed in only 124 of the 244 per-protocol infants, many samples having fallen below the limit of quantification. The source does not report how those 124 observations were distributed across the three groups, and no reported quantity determines that split. For the reference analysis the 124 were apportioned in the same 69:81:88 ratio, giving 36 (L-5-MTHF), 42 (folic acid), and 46 (reference); this step is an assumption rather than a derivation, and it is labelled as such in Supplementary Table S4. Because missingness was generated by values falling below the limit of quantification and the L-5-MTHF arm had systematically lower UMFA, a proportional split is expected to overstate the number of quantifiable observations retained in that arm, which makes the assumption conservative with

respect to the reported effect. Supplementary Table S4b Panel A reports the trial and pooled estimates across the full plausible range of allocations, and Panel B quantifies the direction of the censoring bias by simulation. UMFA was reported as medians with interquartile ranges and was converted using Wan et al. Method 3 with the η(n) denominator specified in Section 1 (see deviation D13, which corrects an earlier use of the asymptotic constant IQR/1.35).

For **Cochrane 2024 [83]** the trial randomized 30 and 30 participants and 54 attended the endline visit. The participant-flow caption reports that the folic-acid group contributed n 19 at the postpartum visit, comprising n 18 who had attended endline plus n 1 who delivered prematurely and did not attend endline, and that n 7 endline participants declined the postpartum draw. This gives 25 folic-acid participants analysed at endline and therefore 29 in the (6S)-5-MTHF arm.

Applying these recovered denominators changes the Troesch erythrocyte-folate interval from +0.40 [+0.14, +0.65] to +0.40 [+0.08, +0.73], leaves the Cochrane erythrocyte-folate estimate unchanged, and changes the Troesch plasma-UMFA contribution to Pool P1 from +1.15 [+0.87, +1.43] to +1.33 [+0.84, +1.82], which was in turn corrected to +1.12 [+0.64, +1.60] when the Wan Method 3 estimator was applied as specified (deviation D13). The Pool P1 pooled estimate and its prespecified-criterion equimolar-only sensitivity analysis are reported with the corrected denominators and the corrected estimator throughout. This replacement of the equal-allocation assumption is declared as deviation D12 in Section 5.

Geometric means and imputed SDs. Where a published trial reported geometric means with confidence intervals, log-transformed effect sizes were derived using the standard log-normal back-transformation. Where SDs were reported only for the FA arm, the 5-MTHF arm SD was imputed from the FA arm SD on the same outcome (a conservative assumption that biases the pooled estimate toward zero).

8. Software and reproducibility.

Implementation. All computations were performed in Python 3.11 with numpy 1.26 and scipy.stats 1.12. The implementation is algorithmically equivalent to R's meta (metagen, metabin, metacont) and metafor (rma.uni with test = "knha") packages.

Cross-validation. Cross-validation of pooled estimates and CIs against R metafor on a worked example (Cochrane 2024 [83] + Draicchio 2026 [84] + Troesch 2019 [82] for Pool P1) returned identical pooled point estimates and HKSJ-adjusted 95% CIs to three decimal places.

Code and data availability. The complete analytic code is preserved as meta_analyses.py and the structured study-level inputs are reproduced in Supplementary Tables S4–S7. Source code is available from the corresponding author on reasonable request.

9. Search strategy.

The literature search underpinning this systematic review followed a PRISMA-2020 [41] structured protocol so that the evidence base can be reproduced and audited, and reporting follows the PRISMA 2020 [41] statement throughout (completed checklist, Supplementary Table S9). The registered primary question—whether equimolar (6S)-5-MTHF substitution alters unmetabolized folic acid, erythrocyte folate, or total homocysteine versus folic acid—was registered in PROSPERO (CRD420261407616) and carries the quantitative synthesis (Pools P1–P3, with exploratory Pools S1, S3, and S4) together with formal GRADE certainty rating. The

contextual mechanistic and observational streams were systematically searched and appraised under the same protocol but were synthesized narratively rather than pooled, because the exposure measured differs across those studies; aggregate GRADE labels are therefore not applied to them. This division of a single systematic review into one pooled registered question and two narratively synthesized contextual streams is stated in Sections 2.2, 2.5, and 2.9 of the main manuscript and is reflected in the location entries of the PRISMA checklist.

PubMed Boolean search (executed 13 May 2026). The full Boolean string is reproduced below; parallel translations were applied to Embase, Cochrane CENTRAL, Scopus, Web of Science, and Google Scholar following the syntactic conventions of each database.

> ("unmetabolized folic acid"[tiab] OR UMFA[tiab] OR "circulating folic acid"[tiab] OR "non-methylated folate"[tiab])
> AND ("folic acid"[MeSH] OR "5-methyltetrahydrofolate"[tiab] OR "L-5-MTHF"[tiab] OR "(6S)-5-methyltetrahydrofolic acid"[tiab] OR "leucovorin"[MeSH] OR "5-formyltetrahydrofolate"[tiab] OR "folinic acid"[tiab])
> AND (humans[MeSH] OR human[tiab] OR animal[MeSH] OR cell[tiab] OR pregnancy[tiab] OR "natural killer"[tiab] OR autism[tiab] OR "folate receptor"[tiab] OR DHFR[tiab] OR MTHFR[tiab])
> AND ("1995/01/01"[PDAT] : "2026/05/13"[PDAT])

Final search strings per source (executed 13 May 2026; coverage 1 January 1995 – 13 May 2026; English-language peer-reviewed publications).

The PubMed Boolean string above is the master strategy. Equivalent strings used for each remaining database, registry, and hand-search source are reproduced verbatim below.

PubMed/MEDLINE (NLM). Executed 13 May 2026.

> ("unmetabolized folic acid"[tiab] OR UMFA[tiab] OR "circulating folic acid"[tiab] OR "non-methylated folate"[tiab]) AND ("folic acid"[MeSH] OR "5-methyltetrahydrofolate"[tiab] OR "L-5-MTHF"[tiab] OR "(6S)-5-methyltetrahydrofolic acid"[tiab] OR "leucovorin"[MeSH] OR "5-formyltetrahydrofolate"[tiab] OR "folinic acid"[tiab]) AND (humans[MeSH] OR human[tiab] OR animal[MeSH] OR cell[tiab] OR pregnancy[tiab] OR "natural killer"[tiab] OR autism[tiab] OR "folate receptor"[tiab] OR DHFR[tiab] OR MTHFR[tiab]) AND ("1995/01/01"[PDAT] : "2026/05/13"[PDAT]) AND English[lang]

Embase (Elsevier, Emtree syntax). Executed 13 May 2026.

> ('unmetabolized folic acid':ti,ab,kw OR 'umfa':ti,ab,kw OR 'circulating folic acid':ti,ab,kw OR 'non-methylated folate':ti,ab,kw) AND ('folic acid'/exp OR '5-methyltetrahydrofolate':ti,ab,kw OR 'l-5-mthf':ti,ab,kw OR '(6s)-5-methyltetrahydrofolic acid':ti,ab,kw OR 'folinic acid'/exp OR '5-formyltetrahydrofolate':ti,ab,kw) AND ('human'/exp OR 'animal'/exp OR 'pregnancy'/exp OR 'natural killer cell'/exp OR 'autism'/exp OR 'folate receptor'/exp OR 'dihydrofolate reductase'/exp OR 'methylenetetrahydrofolate reductase'/exp) AND [english]/lim AND [1995-2026]/py

Cochrane CENTRAL (Cochrane Library, CRSO syntax). Executed 13 May 2026.

> #1 ("unmetabolized folic acid" OR UMFA OR "circulating folic acid"):ti,ab,kw #2 MeSH descriptor: [Folic Acid] explode all trees #3 ("5-methyltetrahydrofolate" OR "L-5-MTHF" OR "5-MTHF" OR

“(6S)-5-methyltetrahydrofolic acid”):ti,ab,kw #4 MeSH descriptor: [Leucovorin] explode all trees #5 (“folinic acid” OR “5-formyltetrahydrofolate”):ti,ab,kw #6 #1 AND (#2 OR #3 OR #4 OR #5) #7 #6 with Publication Year from 1995 to 2026, in Trials

Scopus (Elsevier; TITLE-ABS-KEY syntax). Executed 13 May 2026.

TITLE-ABS-KEY(“unmetabolized folic acid” OR “UMFA” OR “circulating folic acid” OR “non-methylated folate”) AND TITLE-ABS-KEY(“folic acid” OR “5-methyltetrahydrofolate” OR “L-5-MTHF” OR “(6S)-5-methyltetrahydrofolic acid” OR “leucovorin” OR “folinic acid” OR “5-formyltetrahydrofolate”) AND TITLE-ABS-KEY(human* OR animal OR cell OR pregnan* OR “natural killer” OR autism OR “folate receptor” OR DHFR OR MTHFR) AND PUBYEAR > 1994 AND PUBYEAR < 2027 AND LANGUAGE(english) AND DOCTYPE(ar OR re OR cp)

Web of Science Core Collection (Clarivate; topic search). Executed 13 May 2026.

TS=(“unmetabolized folic acid” OR UMFA OR “circulating folic acid” OR “non-methylated folate”) AND TS=(“folic acid” OR “5-methyltetrahydrofolate” OR “L-5-MTHF” OR “(6S)-5-methyltetrahydrofolic acid” OR leucovorin OR “folinic acid” OR “5-formyltetrahydrofolate”) AND TS=(human OR animal OR cell OR pregnan* OR “natural killer” OR autism OR “folate receptor” OR DHFR OR MTHFR) Refined by: Languages = ENGLISH; Document Types = ARTICLE OR REVIEW; Timespan = 1995-01-01 to 2026-05-13.

Google Scholar (allintitle / advanced search). Executed 13 May 2026; first 200 results screened (sorted by relevance and by date 2020–2026) to supplement structured database coverage and identify grey literature.

Query 1: “unmetabolized folic acid” OR “UMFA” 1995..2026 Query 2: “5-methyltetrahydrofolate” “folic acid” trial pregnancy Query 3: “folinic acid” “folate receptor” autoantibody autism Query 4: DHFR human “folic acid” reduction Query 5: MTHFR 677 “folic acid” trial homocysteine genotype

ClinicalTrials.gov (NCT registry). Hand-searched 13 May 2026 to identify trials measuring UMFA or comparing FA with reduced folate forms.

Condition or disease: folate OR folic acid OR autism spectrum disorder OR cerebral folate deficiency Other terms: “unmetabolized folic acid” OR UMFA OR 5-MTHF OR “(6S)-5-methyltetrahydrofolate” OR leucovorin OR “folinic acid” Study status: completed | active, not recruiting | recruiting Filters: 1995/01/01 to 2026/05/13

NCT identifiers extracted from included publications were cross-checked to confirm primary trial registration and to identify duplicate-cohort reporting (e.g., a secondary 2024 FACT-cohort publication identified as overlapping with Murphy 2021 [43]).

US Food and Drug Administration hand-search. The FDA briefing-book hand-search covered publicly available materials from the following advisory committee and workshop venues, queried by document keyword “folate” OR “folic acid” OR “5-methyltetrahydrofolate” OR “UMFA” between 1995 and 13 May 2026: (i) FDA Folate Workshop (2008); (ii) FDA Folate Forms Workshop materials referenced by Bailey 2009 [19] and Macfarlane 2020 [156]; (iii) NIH Office of Dietary Supplements “Roles of folate in human nutrition” 2019 workshop briefing book; (iv) FDA orphan-drug designation public dockets containing “leucovorin” or “folinic

acid" between 2010 and 2026. References extracted from these venues that were also indexed in PubMed/Embase were de-duplicated at the title/DOI level.

Citation chasing. Forward and backward citation chasing was performed on three pivotal references: (i) Kelly et al. 1997 [15] (Am J Clin Nutr; PMID 9174474) — the original UMFA pharmacokinetic paper; (ii) Frye et al. 2018 [36] (Mol Psychiatry; PMID 28948862 / 29317760 depending on edition) — the index leucovorin-in-ASD RCT; (iii) Howick et al. 2011 [42] (CEBM) — the OCEBM 2011 [42] framework reference.

PROSPERO and overlap check. PROSPERO was queried on 13 May 2026 with the same Boolean stem to identify ongoing systematic reviews on UMFA; no overlapping protocol was identified.

Number-of-records reconciliation. Structured database and Google Scholar searches identified 5,630 records; ancillary/manual sources identified 40 additional records; total records identified = 5,670. After de-duplication on title/DOI/PubMed-ID, 3,108 unique records were screened at title/abstract; 210 full-text reports assessed for eligibility, of which 145 were excluded (the 122 exclusions recorded at screening plus 23 publications removed at source re-verification under deviation D17: 3 pre-1995, 7 reviews cited as primary studies, 8 with no retrievable matching publication, and 4 whose verified content did not match the extracted claim); 65 unique publications describing 61 unique primary studies, yielding 71 analytic contributions (15 mechanistic, 24 observational, 32 interventional). Row-level disposition of all 101 originally submitted contributions is given in Supplementary Table S10. The PRISMA 2020 [41] flow diagram is Figure 1 of the main manuscript, and the completed PRISMA 2020 [41] checklist is Supplementary Table S9.
