# Supplementary material for: Unmetabolized Folic Acid: Biology, Epidemiology, and Clinical Consequences: A Systematic Review
Source: Nutrients. 2026 Sep 3;18(17):2887. doi: 10.3390/nu18172887 (PMC13567015; doi:10.3390/nu18172887)
Supplement: Supplementary file 1 [file nutrients-18-02887-s001.zip › Supplementary Table S9 — PRISMA 2020 Checklist.pdf]

Supplementary Table S9. PRISMA 2020 [41] Checklist

Table S9a. PRISMA 2020 [41] Main Checklist (27 items, 42 sub-items)

Section and Topic	Item #	Checklist Item	Location Where Item Is Reported
**TITLE**			
**Title**	**1**	Identify the report as a systematic review.	**Reported: Yes** Page 1
**ABSTRACT**			
**Abstract**	**2**	See the PRISMA 2020 [41] for Abstracts checklist.	**Reported: Yes** Page 1
**INTRODUCTION**			
**Rationale**	**3**	Describe the rationale for the review in the context of existing knowledge.	**Reported: Yes** Section 1.1 (one-carbon metabolism); 1.2 (mandatory fortification); 1.3 (emergence of UMFA); 1.4 (established vs. unestablished actions of folic acid); 1.5 (why UMFA is worth measuring); 1.6 (FRα–cerebral folate deficiency axis).
**Objectives**	**4**	Provide an explicit statement of the objective(s) or question(s) the review addresses.	**Reported: Yes** Section 1.7: one registered primary question stated in PICO form (equimolar (6S)-5-MTHF vs. folic acid; outcomes plasma UMFA, erythrocyte folate, plasma tHcy) plus three explicitly labelled contextual questions. Restated in Section 2.2, paragraph 2.
**METHODS**			
**Eligibility criteria**	**5**	Specify the inclusion and exclusion criteria for the review and how studies were grouped for the syntheses.	**Reported: Yes** Section 2.2 (overall inclusion/exclusion criteria, including excluded designs: editorials, single case reports without mechanistic content, retracted papers, unpublished abstracts, superseded duplicate cohort reports; and reviews, meta-analyses, and other secondary syntheses, which were used for citation chasing and contextual orientation only and are not

Section and Topic	Item #	Checklist Item	Location Where Item Is Reported
			counted as included reports, studies, or analytic contributions; English-language, peer-reviewed, 1995–May 2026) and Section 2.2, paragraph 2 (narrower PICO eligibility for the registered question). Grouping: three evidence streams (mechanistic, observational, interventional); observational studies further grouped a priori into four mutually exclusive exposure classes — Section 2.5 and Table 1.
**Information sources**	**6**	Specify all databases, registers, websites, organisations, reference lists and other sources searched or consulted to identify studies. Specify the date when each source was last searched or consulted.	**Reported: Yes** Section 2.3: PubMed/MEDLINE, Embase, Cochrane CENTRAL, Scopus, Web of Science, and Google Scholar, all searched in May 2026 (coverage January 1995–May 2026, Section 2.2); plus citation chasing of three pivotal references, hand-search of ClinicalTrials.gov, and bibliographies of relevant US FDA briefing books. The Figure 1 identification box names the same six databases (n = 5630) alongside a separate manual-identification box for the non-database sources (n = 40).
**Search strategy**	**7**	Present the full search strategies for all databases, registers and websites, including any filters and limits used.	**Reported: Yes** Section 2.3 (summary; full PubMed string reproduced in Supplementary Methods). Limits/filters: English language, peer-reviewed publications, January 1995–May 2026 (Section 2.2). Supplementary Methods also reports conversion formulae.
**Selection process**	**8**	Specify the methods used to decide whether a study met the inclusion criteria of the review, including how many reviewers screened each record and each report retrieved, whether they worked independently, and if applicable, details of automation tools used in the process.	**Reported: Yes** Section 2.4: two reviewers independently screened titles, abstracts, and full texts; discordances resolved by consensus and, where required, by adjudication from a third reviewer. No automation tools were used (stated in Section 2.6).
**Data collection process**	**9**	Specify the methods used to collect data from reports, including how many reviewers collected data from each report, whether they	**Reported: Yes** Section 2.4: extraction into structured spreadsheets by two reviewers with consensus resolution and third-reviewer adjudication; no automation tools. Source-

Section and Topic	Item #	Checklist Item	Location Where Item Is Reported
		worked independently, any processes for obtaining or confirming data from investigators, and if applicable, details of automation tools used in the process.	anchored re-extraction against primary publications is described in Section 2.8 (e.g., Tam et al.). Investigators were not contacted for unreported data.
**Data items**	**10a**	List and define all outcomes for which data were sought. Specify whether all results that were compatible with each outcome domain in each study were sought (e.g. for all measures, time points, analyses), and if not, the methods used to decide which results to collect.	**Reported: Yes** Section 2.2 (primary outcome: detection or concentration of UMFA in any biological compartment, or any biological or clinical endpoint plausibly attributable to folate form or dose) and Section 2.2, paragraph 2 (registered PICO outcomes: plasma/serum UMFA, erythrocyte folate, plasma tHcy). Section 2.8 states the rule for multiplicity across reports of the same cohort (index publication retained only; overlapping FACT metabolomics report removed).
	**10b**	List and define all other variables for which data were sought (e.g. participant and intervention characteristics, funding sources). Describe any assumptions made about any missing or unclear information.	**Reported: Partly - study-level funding sources were not extracted** Section 2.4 (population, design, intervention, dose, duration, UMFA assay methodology, effect estimates with 95% CI, risk-of-bias domains); Section 2.5 (exposure metric classification); Section 2.7 (assay generation and fasting status tagging). Assumptions about missing data are specified in Section 2.8: median/IQR and median/range conversion using Wan et al. Methods 3 and 1, recovery of SD from SE as SE×√n, and molar conversion at 441.4 g/mol. Study-level funding sources were not extracted.
**Study risk of bias assessment**	**11**	Specify the methods used to assess risk of bias in the ǀ **Reported: Yes** Section 2.6: Cochrane RoB 2 for randomized trials, ROBINS-I included studies, including details of the tool(s) used, ǀ for non-randomized human studies, SYRCLE for animal experiments; each study how many reviewers assessed each study and whether they ǀ appraised independently by two reviewers with consensus and third-reviewer worked independently, and if applicable, details of ǀ adjudication; no automation tools. OCEBM 2011 [42] Levels of Evidence applied automation tools used in the process. ǀ descriptively to human studies.	
**Effect measures**	**12**	Specify for each outcome the effect measure(s)	**Reported: Yes** Section 2.8: Hedges' g standardized mean

Section and Topic	Item #	Checklist Item	Location Where Item Is Reported
		(e.g. risk ratio, mean difference) used in the synthesis or presentation of results.	difference with small-sample correction J for continuous biomarkers on heterogeneous assays (plasma UMFA, plasma 5-MTHF, erythrocyte folate); raw mean difference in μmol/L for plasma tHcy; raw mean difference in nmol/L for the dose-response pool (Section 3.3, Pool S3). Observational effect measures are reported as published (OR, RR, HR) in Sections 3.6.1–3.6.4 without pooling.
**Synthesis methods**	**13a**	Describe the processes used to decide which studies were eligible for each synthesis (e.g. tabulating the study intervention characteristics and comparing against the planned groups for each synthesis).	**Reported: Yes** Section 2.8: meta-analysis performed only where at least three independent studies shared population, exposure, comparator, and outcome metric. Study characteristics tabulated in Supplementary Tables S1–S3 by stream; observational studies assigned to exposure classes in Table 1 (Section 2.5), with UMFA-specific conclusions drawn from Class 1 only.
	**13b**	Describe any methods required to prepare the data for presentation or synthesis, such as handling of missing summary statistics, or data conversions.	**Reported: Yes** Section 2.8: Wan et al. Methods 3 and 1 for median with IQR and median with range, using the sample-size-dependent η(n) denominator rather than the asymptotic constant IQR/1.35 (deviation D13); SD recovered from SE as SE×√n; UMFA converted from ng/mL to nmol/L using folic acid molecular weight 441.4 g/mol; only the index publication retained for overlapping cohort reports. Full conversion formulae in Supplementary Methods.
	**13c**	Describe any methods used to tabulate or visually display results of individual studies and syntheses.	**Reported: Yes** Supplementary Tables S1–S3 (descriptive evidence profiles by stream), Table 1 (observational exposure classification), Supplementary Table S4 (Pool P1 inputs), Supplementary Table S5 (Pool P2 and P3 inputs), Supplementary Table S6 (cord-UMFA × ASD cohort evidence), Supplementary Table S7 (Pool S3 and S4 inputs); trials grouped into Pools P1–P3 and S1, S3, S4 per Section 2.8; forest plot in Figure 2 (Panels A–B; Pool P3 is qualitative because its retained reports lack

Section and Topic	Item #	Checklist Item	Location Where Item Is Reported
			recoverable per-arm variances) and Supplementary Figures S1, S3, S3b, and S4; Supplementary Figure S2 (cord-UMFA × ASD); Figure 3 (identifiability of UMFA-specific effects). Funnel plots were not produced (no pool reached an interpretable number of studies).
	**13d**	Describe any methods used to synthesise results and provide a rationale for the choice(s). If meta-analysis was performed, describe the model(s), method(s) to identify the presence and extent of statistical heterogeneity, and software package(s) used.	**Reported: Yes** Section 2.8 and Supplementary Methods Sections 1–3: retained meta-analyses use a random-effects DerSimonian–Laird model with the Hartung–Knapp–Sidik–Jonkman small-sample adjustment; Pool P2 is not pooled and Pool P3 is qualitative because its retained reports do not permit a defensible common-variance estimate. Heterogeneity is quantified by τ^2 and I^2. Prediction intervals are computed only for pools with $k \geq 5$; no pool reaches that threshold, so none is reported. Software is Python 3.11 (numpy 1.26, scipy.stats 1.12), algorithmically equivalent to R meta and metafor. Rationale for narrative synthesis of the mechanistic and observational streams is given in Sections 2.1 and 2.5.
	**13e**	Describe any methods used to explore possible causes of heterogeneity among study results (e.g. subgroup analysis, meta-regression).	**Reported: Yes** Section 2.8: pre-specified subgroups were population, exposure dose, assay generation, trial duration, and risk-of-bias domain. Meta-regression was not performed because no pool exceeded four studies. Assay-generation and fasting-status considerations are set out in Section 2.7.
	**13f**	Describe any methods used to assess robustness of the synthesised results.	**Reported: Yes** Section 2.8: pre-specified sensitivity analyses were restriction to LC-MS/MS-assayed trials, leave-one-out resampling, and addition of one matched trial reporting an alternative compartment. One exploratory post-hoc sensitivity analysis excluding Tam et al. is described and labelled as post hoc (Section 2.8; results in Section 3.3 and Supplementary Figure S3b).

Section and Topic	Item #	Checklist Item	Location Where Item Is Reported
**Reporting bias assessment**	**14**	Describe any methods used to assess risk of bias due to missing results in a synthesis (arising from reporting biases).	**Reported: Partly - no funnel plots or Egger's test were performed** Section 2.8: Egger's test was pre-specified for pools with ≥ 10 studies and, no pool reaching that threshold, was not performed; funnel plots were not produced because no pool contained a sufficient number of studies for a funnel plot to be interpretable. Recorded as deviation D6 in Supplementary Methods Section 5; consequence stated in Sections 3.4 and 4.9.
**Certainty assessment**	**15**	Describe any methods used to assess certainty (or confidence) in the body of evidence for an outcome.	**Reported: Partly - certainty assessment is limited to the registered PICO** Section 2.9: GRADE applied to the registered PICO question, with up- or down-grading for risk of bias, inconsistency, indirectness, imprecision, publication bias, dose-response gradient, magnitude of effect, and plausible residual confounding. Section 2.1 states and justifies the a priori decision not to assign aggregate GRADE ratings to the heterogeneous contextual streams; those are appraised descriptively, with OCEBM levels (Section 2.6).
**RESULTS**			
**Study selection**	**16a**	Describe the results of the search and selection process, from the number of records identified in the search to the number of studies included in the review, ideally using a flow diagram.	**Reported: Yes** Section 3.1 and Figure 1 (PRISMA flow diagram): 5670 records identified across six structured databases, Google Scholar, and ancillary sources; 3108 unique records screened after deduplication; 210 reports assessed at full text, of which 145 were excluded (122 at screening plus 23 removed at source re-verification under deviation D17); 65 unique publications describing 61 unique primary studies, yielding 71 analytic contributions (15 mechanistic, 24 observational, 32 interventional), of which 51 contribute human participant data. Row-level disposition of all 101 originally submitted contributions is in Supplementary Table S10.

<table>
<tr><th>Section and Topic</th><th>Item #</th><th>Checklist Item</th><th>Location Where Item Is Reported</th></tr>
<tr><td></td><td>16b</td><td colspan="2">Cite studies that might appear to meet the inclusion | Reported: Yes Figure 1 reports the 145 full-text exclusions against eleven criteria, but which were excluded, and explain why they | named reasons, which sum to the stated total and reconcile 210 reports assessed were excluded. | at full text with 65 included publications. Seven reasons were applied during | screening and extraction: UMFA not measured (n = 41); pediatric acute | lymphoblastic leukemia or methotrexate studies (n = 22); conference abstract | only (n = 12); duplicate or companion report of an already-included | publication, identified either at screening or at extraction (n = 27); | retracted publication (n = 1); review, meta-analysis, or other secondary | synthesis retained for citation chasing only (n = 3); other reasons (n = 16) | — 122 in total. Complete source re-verification before this revision | (deviation D17) added four further reasons: publication predated the 1 January | 1995 eligibility window (n = 3); review or other secondary synthesis that had | been cited as a primary study (n = 8); no publication matching the cited | reference could be retrieved by author, title, DOI or topic search (n = 8); and | verified content of the cited publication did not match the extracted claim and | supplied no other eligible contribution (n = 4) — 23 in total. The reasons | are repeated in the Figure 1 caption, and the row-level disposition of every | affected entry is given in Supplementary Table S10. Category-level exclusion | criteria are specified in Section 2.2. Individual excluded reports that might | appear eligible are cited and explained in text: Section 2.8 (a 2024 | FACT-cohort metabolomics publication removed as an overlapping report of Murphy | et al.), Section 3.2 (the Schön/Obeid 2024 [85] entry removed from Pool P1 because | the source evaluates a 1:1 folic acid/(6S)-5-MTHF mixture rather than a | head-to-head contrast), and Section 3.2 (Houghton 2006 [88] removed from Pool P3 | because its folate-form contrast was not randomized, deviation D18).</td></tr>
<tr><td>Study characteristics</td><td>17</td><td>Cite each included study and present its characteristics.</td><td>Reported: Yes Supplementary Table S1 (descriptive evidence profile, mechanistic studies), Supplementary Table S2 (descriptive evidence profile, observational studies, with exposure classification), Supplementary Table S3 (descriptive evidence profile, interventional trials); Table 1 (observational exposure classes and inferential weight). Narrative characteristics in Sections 3.5, 3.6.1–3.6.5, and 3.7. All included studies are cited in the reference list.</td></tr>
<tr><td>Risk of bias in studies</td><td>18</td><td colspan="2">Present assessments of risk of bias for each included | Reported: Yes Per-study risk-of-bias judgements are presented for every study. | included study in the descriptive evidence profiles: Supplementary Table S1 | (SYRCLE for animal experiments; not applicable for cell-culture and in vitro | enzymology), Supplementary Table S2 (ROBINS-I with OCEBM 2011 [42] levels), and | Supplementary Table S3 (RoB 2.0 for randomized trials and</td></tr>
</table>

<table>
<tr><th>Section and Topic</th><th>Item #</th><th>Checklist Item</th><th>Location Where Item Is Reported</th></tr>
<tr><td></td><td></td><td colspan="2">ROBINS-I for | non-randomized human interventions, including case-series designs, with OCEBM | 2011 levels; reviews and other secondary syntheses are excluded from that table | and receive no trial judgement). Domain-level judgements for the three | head-to-head trials contributing to the registered question are given in full | in the Supplementary Table S3 footnotes, and the Troesch 2019 [82] judgement is | reported per outcome because the analysed denominators differ across its | outcomes. Section 3.4 summarizes risk of bias for the head-to-head trials | contributing to the registered question and Section 3.1 records that all 51 | studies contributing human participant data were appraised, with pre-2010 | affinity-HPLC studies flagged for assay-generation effects. Judgements are | reported at study level rather than as separate per-domain matrices.</td></tr>
<tr><td>Results of individual studies</td><td>19</td><td>For all outcomes, present, for each study: (a) summary statistics for each group (where appropriate) and (b) an effect estimate and its precision (e.g. confidence/credible interval), ideally using structured tables or plots.</td><td>Reported: Partly - P3 lacks recoverable arithmetic summary statistics Figure 2, Panels A–B (study-level estimates with 95% CI for Pools P1–P2; P3 is presented qualitatively because arithmetic per-arm variances are not recoverable); Supplementary Table S4 (Pool P1 study-level inputs), Supplementary Table S5 (Pool P2 and P3 study-level inputs), Supplementary Table S7 (Pool S3 and S4 study-level inputs); Supplementary Figures S1, S3, S3b, and S4 (forest plots). Per-trial mean differences for Pool S3 are additionally reported in text (Section 3.3). Individual observational estimates with 95% CI are reported in Sections 3.6.1–3.6.4 and Supplementary Table S6; individual mechanistic findings in Section 3.5 and Supplementary Table S1. Pool S1 has no separate input table; its three constituent estimates are shown in Supplementary Figure S1.</td></tr>
<tr><td>Results of syntheses</td><td>20a</td><td>For each synthesis, briefly summarise the characteristics and risk of bias among contributing studies.</td><td>Reported: Yes Sections 3.2 and 3.3 (contributing trials identified by citation and by population, dose, and duration for each pool); Section 3.4 with Supplementary Table S3 (risk of bias among contributing trials) and Supplementary Table S8 (GRADE summary of findings); Section 3.5 (mechanistic stream); Section 3.6 with Table 1 (observational stream by exposure class); Section 3.7 (interventional pharmacology stream).</td></tr>
</table>

<table>
<tr><th>Section and Topic</th><th>Item #</th><th>Checklist Item</th><th>Location Where Item Is Reported</th></tr>
<tr><td></td><td>20b</td><td>Present results of all statistical syntheses conducted. If meta-analysis was done, present for each the summary estimate and its precision (e.g. confidence/credible interval) and measures of statistical heterogeneity. If comparing groups, describe the direction of the effect.</td><td>Reported: Yes Section 3.2 — Pool P1 plasma UMFA: SMD +0.99 (95% HKSJ CI +0.20 to +1.79; $p = 0.033$; $I^2 = 30\%$; $k = 3$); Pool P2 erythrocyte folate: not pooled ($k = 2$; Hedges' g +0.40, +0.08 to +0.73 and −0.38, −0.92 to +0.16; $I^2 = 83\%$); Pool P3 plasma tHcy: pooling abandoned, so no pooled estimate is reported and the outcome is synthesized qualitatively. Section 3.3 — Pool S1 plasma 5-MTHF: SMD +0.48 (−0.80 to +1.76; $I^2 = 78\%$); Pool S3 dose-response: MD −1.2 nmol/L (−33.7 to +31.4; $I^2 = 85\%$); Pool S4 genotype-stratified tHcy responses by MTHFR 677 genotype. No prediction interval is reported because no pool reaches $k = 5$.</td></tr>
<tr><td></td><td>20c</td><td>Present results of all investigations of possible causes of heterogeneity among study results.</td><td>Reported: Yes Section 3.2, final paragraph (residual heterogeneity in Pool P1 attributed to population, dose, duration, and assay generation rather than direction of effect); Section 3.3 (heterogeneity in Pool S1 attributed to assay sensitivity and dose, with the pool designated hypothesis-generating; the negative Pool S3 estimate traced to a single low-dose outlier on the Wan range-estimated scale); Section 3.4 (indirectness from heterogeneous populations); Section 4.9 (assay generation, fasting status, and genotype as effect modifiers). Planned subgroup analyses were not separately reportable because each pool comprised only three to four trials.</td></tr>
<tr><td></td><td>20d</td><td colspan="2">Present results of all sensitivity analyses conducted to | Reported: Partly - only reported sensitivity results are listed Section assess the robustness of the synthesised results. | 3.2 reports the prespecified-criterion equimolar-only sensitivity analysis | excluding Draicchio 2026 [84] from Pool P1: Hedges' g +1.17 (+0.32 to +2.03), $p =$ | 0.036, $I^2 =$ 0%, $k = 2$ — direction and magnitude preserved, heterogeneity | eliminated, and the interval excludes the null. Supplementary Table S4b | reports two further sensitivity analyses added in revision (deviation D14): | Panel A recomputes Pool P1 across the full plausible range of assumed per-arm | allocations for the 124 quantifiable UMFA observations in Troesch 2019 [82] (pooled | estimate +0.96 to +1.00, every interval excluding the null), and Panel B | quantifies by simulation the direction of bias introduced by | limit-of-quantification censoring (attenuation toward the null of | approximately 0.15 to 0.18). Section 3.3 and Supplementary Figure S3b report | the post-hoc exclusion</td></tr>
</table>

Section and Topic	Item #	Checklist Item	Location Where Item Is Reported
		of Tam et al. from Pool S3: MD +4.6 nmol/L (95% HKSJ CI ǀ −58.0 to +67.2; I^2 = 69%). Other leave-one-out analyses were computed but are ǀ not individually displayed.	
**Reporting biases**	**21**	Present assessments of risk of bias due to missing results in each synthesis.	**Reported: Partly - reporting bias could not be formally assessed** Section 3.4: publication bias could not be formally assessed in any pool because no pool reached the pre-specified threshold of ≥ 10 studies for Egger's test, and funnel plots were not produced for the same reason (Supplementary Methods Section 5, deviation D6). Restated as a limitation in Section 4.9. Section 3.7 additionally notes that none of the large folic acid cardiovascular and cognitive trials measured UMFA.
**Certainty of evidence**	**22**	Present assessments of certainty (or confidence) in the ǀ **Reported: Partly - formal GRADE applies only to the registered PICO** Section body of evidence for each outcome assessed. ǀ 3.4: GRADE low for plasma UMFA (Pool P1), downgraded once for serious risk of ǀ bias (two of three contributing trials at high risk of bias for this outcome) ǀ and once for serious imprecision (the pooled interval spans small to very large ǀ effects on a combined analysed sample of fewer than 200 participants, and its ǀ lower bound is additionally conditional on an assumed per-arm allocation in the ǀ largest trial, falling from +0.20 to +0.13 across the allocations tested in ǀ Supplementary Table S4b; following correction of the effect-size derivation in ǀ deviation D13 the prespecified-criterion equimolar-only sensitivity analysis ǀ excludes the null and is no longer cited in support of this downgrade); GRADE ǀ very low for erythrocyte folate (Pool P2), downgraded once for serious ǀ inconsistency and twice for very serious imprecision, because two single-level ǀ downgrades from an initial rating of high would yield low rather than very low ǀ (correction made in revision, deviation D15); and GRADE very low for plasma ǀ tHcy (Pool P3), downgraded twice for very serious imprecision, because no ǀ pooled estimate is derivable and no non-inferiority margin was prespecified, ǀ and once for serious risk of bias from incomplete outcome reporting in both ǀ contributing reports (endline values available only in an unretrievable online ǀ supplement in one trial, and no recoverable per-arm variances in the other); ǀ the non-randomized comparator previously cited in support of this downgrade is ǀ no longer part of the pool, Houghton 2006 [88] having been removed under deviation ǀ D18. Section 3.6.6 gives descriptive certainty for the observational stream ǀ (OCEBM 2011 [42] Levels 2a–5, enumerated per level and matching the per-study ǀ values in Supplementary Table S2 and the range shown in Figure 1). Within that ǀ stream the evidence is inconclusive for directly measured UMFA and offspring ǀ ASD, and consistent and predominantly protective for folic acid supplementation ǀ at recommended doses. Section 2.1 explains why aggregate GRADE	

<table>
<tr><th>Section and Topic</th><th>Item #</th><th>Checklist Item</th><th>Location Where Item Is Reported</th></tr>
<tr><td></td><td></td><td colspan="2">labels are not | applied to contextual evidence. No patient-important clinical outcome was | graded, because none was reported by any head-to-head trial of folate forms; | all three certainty ratings describe biochemical outcomes only, and no | certainty rating for clinical benefit or harm is offered anywhere in this | Review. The Figure 1 inclusion box reports the same three GRADE ratings and no | others, and reports risk-of-bias and OCEBM 2011 [42] judgements as</td></tr>
<tr><td>DISCUSSION</td><td></td><td></td><td></td></tr>
<tr><td>Discussion</td><td>23a</td><td>Provide a general interpretation of the results in the context of other evidence.</td><td>Reported: Yes Section 4.1 (principal findings for the registered question, interpreted as hypothesis-strengthening rather than confirmatory); Section 4.2 (what folic acid is and is not established to do, including CDC safety syntheses); Section 4.4 (neurodevelopmental literature in context, approximately 497,000 pregnancies across ten non-overlapping analyses and four meta-analyses); Section 4.5 (vitamin B12 dependence of homocysteine remethylation); Section 4.6 (FRAA, cerebral folate deficiency, and leucovorin); Section 4.7 (genotype-aware folate strategy); Section 4.8 (public-health framing).</td></tr>
<tr><td></td><td>23b</td><td>Discuss any limitations of the evidence included in the review.</td><td>Reported: Yes Section 4.3 (UMFA and total folate are not separately identifiable; collinearity, measurement error relative to dynamic range, and time-since-ingestion conflation) and Section 4.9, limitations 1–6 (identifiability; two to three small trials per pool; 2–3-fold overestimation by pre-2010 affinity-HPLC assays through MeFox co-elution; inconsistent reporting of fasting status and time since ingestion; large inter-individual variation with few genotype-stratified studies; no UMFA measurement in the large folic acid trials). Also Sections 3.5 and 3.6.4 on supraphysiologic animal exposures and ecological designs.</td></tr>
<tr><td></td><td>23c</td><td>Discuss any limitations of the review processes used.</td><td>Reported: Yes Section 4.9, limitations 7 and 8: the search was restricted to English-language publications, and the contextual</td></tr>
</table>

Section and Topic	Item #	Checklist Item	Location Where Item Is Reported
			streams were not GRADE-rated. Section 2.5 discloses that admitting folic acid intake, total folate, and fortification studies as proxies for UMFA exposure is a defensible sampling frame but not a valid basis for causal attribution to UMFA. Section 3.4 and Section 4.9 note that publication bias could not be formally assessed.
	**23d**	Discuss implications of the results for practice, policy, and future research.	**Reported: Yes** Practice and policy: Sections 4.1 (5-MTHF substitution requires pre-registered confirmatory trials before any change to obstetric practice), 4.6 (no change to perinatal folate recommendations warranted; leucovorin use in FRAA-positive ASD or pregnancy is a question for trials rather than practice), 4.7 (research question, not a basis for genotype-guided supplementation), 4.8 (stewardship of discretionary high-dose supplementation; the review provides no evidence to support changing fortification policy), and Section 5. Future research: Section 4.10 (research priorities) and Section 5.
**OTHER INFORMATION**			
**Registration and protocol**	**24a**	Provide registration information for the review, including register name and registration number, or state that the review was not registered.	**Reported: Yes** Section 2.1 and Abstract (Methods): registered a priori with PROSPERO, registration number CRD420261407616. Also stated in the Data Availability Statement.
	**24b**	Indicate where the review protocol can be accessed, or state that a protocol was not prepared.	**Reported: Partly - only the PROSPERO registration record is identified** Section 2.1 and Data Availability Statement: the review protocol is publicly registered and accessible via PROSPERO (CRD420261407616).
	**24c**	Describe and explain any amendments to information provided at registration or in the protocol.	**Reported: Yes** Supplementary Methods Section 5 ("Protocol deviations and post-hoc analyses") consolidates every departure from the registered protocol in one place, each with its rationale and consequence for the reported estimates. This

Section and Topic	Item #	Checklist Item	Location Where Item Is Reported
		includes D9, the declared post-registration amendment ∣ retaining Draicchio 2026 [84] in Pool P1 only despite approximately 588 μg ∣ (6S)-5-MTHF versus 800 μg FA (approximately 40% more folate on a molar basis ∣ in the FA arm), other differing B vitamins, choline, and vitamin D, and ∣ per-protocol-only analysis; Supplementary Table S4 reports the corresponding ∣ prespecified-criterion equimolar-only sensitivity analysis excluding ∣ Draicchio. The other deviations remain D1 four-class observational-exposure ∣ stratification; D2 Schön/Obeid 2024 [85] removal from Pool P1; D3 duplicate-cohort ∣ removal from Pool S3; D4 post-hoc Tam sensitivity analysis; D5 narrative ∣ single-cohort cord-UMFA × ASD synthesis; D6 nonperformance of Egger’s test and ∣ funnel plots; D7 imputations not specified at registration; D8 scope of formal ∣ GRADE; D10 the prediction-interval rule (computed only for pools with $k \geq 5$), ∣ which the registered protocol did not address and which is therefore stated as ∣ an implemented analysis rule rather than a prespecified one; D11 the rule for ∣ withholding a pooled estimate, also not addressed at registration, under which ∣ no pooled estimate is reported for Pool P2 or Pool P3; D12 replacement of the ∣ equal-allocation assumption used in the original submission with per-arm ∣ denominators derived from the source publications; D13 correction of the Wan ∣ Method 3 effect-size derivation for the two median-reported Pool P1 trials, ∣ which had been computed with the median substituted for the mean and the SD ∣ approximated as IQR/1.35 rather than IQR/η(n); and D14 the addition of ∣ allocation and limit-of-quantification censoring sensitivity analyses for ∣ Troesch 2019 [82], reported as Supplementary Table S4b.	
**Support**	**25**	Describe sources of financial or non-financial support for the review, and the role of the funders or sponsors in the review.	**Reported: Yes** Funding statement: this research received no external funding. Conflicts of Interest statement: the funders had no role in the design of the study; in the collection, analyses, or interpretation of data; in the writing of the manuscript; or in the decision to publish the results.
**Competing interests**	**26**	Declare any competing interests of review authors.	**Reported: Yes** Conflicts of Interest statement: prior involvement in randomized trials of folinic acid in FRAA-positive ASD and in ongoing Investigational New Drug development for leucovorin; R.E.F. is CEO/CSO and D.A.R. is a clinical advisor to Eletala Enterprises Inc.; neither author receives salary or advisory fees from Eletala. Readers are directed to weigh the leucovorin discussion (Section 4.6) accordingly, and the disclosure is cross-referenced in Section 4.6.

Section and Topic	Item #	Checklist Item	Location Where Item Is Reported
**Availability of data, code and other materials**	**27**	Report which of the following are publicly available and where: template data collection forms; data extracted from included studies; data used for all analyses; analytic code; any other materials used in the review.	**Reported: Yes** The Data Availability Statement names each category and where it is available. Template data-collection form: the extraction fields and the arm-level extraction rules applied independently by both reviewers are specified in Supplementary Methods Section 7, and the blank form is available from the corresponding author on reasonable request. Data extracted from included studies: Supplementary Tables S1–S3 (descriptive evidence profiles, with row-level disposition in Supplementary Table S10) and Supplementary Tables S4–S7 (study-level meta-analytic inputs). Data used for all analyses: Supplementary Tables S4–S7, with the conversion formulae applied to reported summary statistics in Supplementary Methods Sections 1–3. Analytic code: meta_analyses.py (Python 3.11, numpy 1.26, scipy 1.12), described in Supplementary Methods Section 8 and available from the corresponding author on reasonable request. Other materials: search strings per source in Supplementary Methods Section 9. The protocol is publicly registered at PROSPERO (CRD420261407616). No separate data repository is used; all review materials are distributed as Supplementary Materials with the article.

Table S9b. PRISMA 2020 [41] for Abstracts Checklist (12 items)

Section and Topic	Item #	Checklist Item	Location in the Abstract
**Title**	**1**	Identify the report as a systematic review.	**Reported: Yes** Title ends ": A Systematic Review"; the Abstract Methods sentence states "We systematically searched six databases".
**Objectives**	**2**	Provide an explicit statement of the main objective(s) or question(s) the review addresses.	**Reported: Yes** Abstract, Background and Methods: the registered PICO question is stated in full (whether equimolar (6S)-5-MTHF substitution alters UMFA, erythrocyte folate, or

Section and Topic	Item #	Checklist Item	Location in the Abstract
			tHcy versus folic acid).
**Eligibility criteria**	**3**	Specify the inclusion and exclusion criteria for the review.	**Reported: Partly - detailed design restrictions and exclusions are outside the abstract** Abstract, Methods: the registered PICO population, intervention, comparator, and outcomes are given, and the three evidence streams are enumerated. Design restrictions and exclusions are not stated within the word limit; full criteria are in Section 2.2.
**Information sources**	**4**	Specify the information sources used to identify studies and the date when each was last searched.	**Reported: Yes** Abstract, Methods: six databases, January 1995–May 2026.
**Risk of bias**	**5**	Specify the methods used to assess risk of bias in the included studies.	**Reported: Partly - GRADE is stated but design-specific risk-of-bias tools are not named** Abstract, Methods: GRADE rating is stated. The design-specific tools (RoB 2, ROBINS-I, SYRCLE) are not named in the Abstract because of the word limit; they are specified in Section 2.6.
**Synthesis of results**	**6**	Specify the methods used to present and synthesise results.	**Reported: Yes** Abstract, Methods: Hartung–Knapp–Sidik–Jonkman random-effects models for the registered question; narrative synthesis with classification by exposure metric for the mechanistic and observational streams.
**Included studies**	**7**	Give the total number of included studies and participants and summarise relevant characteristics of studies.	**Reported: Partly - no total participant count is reported** Abstract, Methods: 65 unique publications describing 61 unique primary studies, yielding 71 analytic contributions (15 mechanistic, 24 observational, 33 interventional). A total participant count is not given because included designs range from in vitro experiments to population sur
**Synthesis of results**	**8**	Present results for main outcomes, preferably indicating the number of included studies and participants for each. If meta-analysis was done,	**Reported: Partly - P2 is not pooled and P3 pooling was abandoned** Abstract, Results: UMFA pooled estimate with 95% CI, I^2, and k (SMD +0.99, +0.20 to +1.79, I^2 = 30%, k = 3); the two

Section and Topic	Item #	Checklist Item	Location in the Abstract
		report the summary estimate and confidence interval. If comparing groups, indicate the direction of the effect.	erythrocyte-folate trials are reported individually and not pooled because they are directionally discordant; and tHcy is reported qualitatively with no pooled estimat
**Limitations of evidence**	**9**	Provide a brief summary of the limitations of the evidence included in the review.	**Reported: Partly - the abstract does not fully detail the small-pool limitations** Abstract, Results: observational UMFA–outcome associations described as inconsistent, confounded by total folate, and unreplicated.
**Interpretation**	**10**	Provide a general interpretation of the results and important implications.	**Reported: Yes** Abstract, Conclusions: substitution of (6S)-5-MTHF for folic acid appears to reduce circulating UMFA; with two to three trials per outcome and no prespecified margins, the evidence cannot support equivalence or non-inferiority, and clinical benefit remains unestablished.
**Funding**	**11**	Specify the primary source of funding for the review.	**Reported: Yes** Abstract, Conclusions: "Funding: no external funding." Consistent with the Funding statement in the back matter. The Abstract is 193 words, within the 200-word limit.
**Registration**	**12**	Provide the register name and registration number.	**Reported: Yes** Abstract, Methods: PROSPERO CRD420261407616.
