# Supplementary material for: Unmetabolized Folic Acid: Biology, Epidemiology, and Clinical Consequences: A Systematic Review
Source: Nutrients. 2026 Sep 3;18(17):2887. doi: 10.3390/nu18172887 (PMC13567015; doi:10.3390/nu18172887)
Supplement: Supplementary file 1 [file nutrients-18-02887-s001.zip › Supplementary Tables S1–S8 S10 Figure S1-S4.pdf]

Supplementary Table S1: Descriptive Evidence Profile, Mechanistic Studies (n = 15). Mechanistic evidence is appraised descriptively using per-study risk of bias (SYRCLE for animal experiments; not applicable for cell-culture or in vitro enzymology) and an indicative certainty judgement; these per-study judgements do not constitute an aggregate certainty rating for the mechanistic evidence base, which is not the focused PICO question of this narrative review.

Operational definition of descriptive-contribution labels (applied per analytic contribution, not as GRADE certainty). Substantive requires all three conditions: a controlled human intervention/crossover, direct human pharmacokinetic/assay-validation study, or direct mechanistic experiment; direct measurement of the stated exposure and outcome; and either independent replication or a validated reference method with corroborating data. **Supporting** requires an informative but less definitive design (for example, an observational, animal, in-vitro, or limited controlled study), direct or near-direct measurement, and no contradictory evidence; independent replication strengthens but is not required for this label. **Suggestive** applies when the design is hypothesis-generating or materially limited, the finding is unreplicated or inconsistent, and/or the exposure or outcome is indirect; it is not used to support a causal conclusion.

#	First Author, Year	Design	Key Finding	RoB / SYRCLE	Contribution
M1	Bailey & Ayling 2009 [19]	Ex vivo DHFR kinetics, human (6 donor livers) vs rat	Human hepatic DHFR activity ~1.4% of rat, with ~5-fold inter-individual variation	Low	Substantive
M2	Patanwala 2014 [20]	Randomized crossover hepatic-portal stable-isotope PK (n = 6)	80 ± 12% of labelled portal folate was unmodified folic acid at 15 min	Low	Substantive
M3	Kelly 1997 [15]	Acute within-subject feeding and single-dose studies; randomization not stated	UMFA appears above ~266 µg/meal in the food regimen and above ~200 µg for single doses	SC	Substantive
M4	Sweeney 2007 [16]	Non-randomized fixed-order dose-descending fortified bread, n = 20 (19 completed)	UMFA detected at 400 µg/d (2 × 200 µg per occasion) but not at 200 or 100 µg/d	SC	Substantive
M5	Bagdasarian 2026 [21]	Non-human-primate [^{18}F]AzaFol PET with kinetic modelling	Folic acid blocks FRα tracer uptake at plasma folic acid 63–90 ng/mL	Low	Supporting

#	First Author, Year	Design	Key Finding	RoB / SYRCLE	Contribution
M7	Paniz 2017 [24]	Single-arm uncontrolled pre-post, FA 5 mg/d × 90 d, n = 30	Serum folate rose ~5-fold and UMFA was detectable in 96.6% at day 45, while NK-cell number and cytotoxicity fell	ROBINS-I High	Supporting
M8	Barua 2014 [27]	Mouse periconceptional FA (0.4 vs 4 mg/kg diet); brain microarray, qRT-PCR/Western, behaviour	Altered offspring brain gene expression and behaviour; *Igf2* methylation was not an outcome of this study	SC	Supporting
M9	Mikael 2013 [28]	Mouse maternal 10× FA diet ± *Mthfr* deficiency; E14.5 histology	Aberrant cardiac development	SC	Supporting
M12	Kalmbach 2008 (*J Nutr* 138:2323) [91]	Cross-sectional genotype × intake analysis, 1215 Framingham Offspring participants	DHFR 19-bp del/del associated with a higher prevalence of high plasma UMFA at folic acid intake ≥500 μg/d (47.0% vs 24.4% wild type; p for interaction = 0.03) and lower erythrocyte folate at intake <250 μg/d; enzyme activity was not measured	Low	Supporting
M13	Christensen 2015 (*Am J Clin Nutr* 101:646) [148]	Mouse 10× FA diet × *Mthfr* genotype, 6 mo	High folic acid inhibited MTHFR activity in vitro and lowered MTHFR protein, 5-methylTHF and SAM/SAH, with hepatocyte degeneration	SC	Supporting
M18	Pfeiffer 2004 (assay)	Stable-isotope-	Validated UMFA	n/a	Substantive

#	First Author, Year	Design	Key Finding	RoB / SYRCLE	Contribution
	[149]	dilution LC-MS/MS candidate reference method, 42 human sera	quantification against radioassay and microbiologic assay		
M20	Kalmbach 2008 (*Am J Clin Nutr* 88:763) [45]	Cross-sectional plasma affinity-HPLC, Framingham Offspring before and after fortification	Circulating folic acid is measurable by affinity-HPLC and was higher after fortification	n/a	Supporting
M26	Pickell 2011 [29]	Mouse 20× FA diet × *Mthfr* genotype; E10.5/E14.5	Embryonic delay and growth retardation; maternal MTHFR deficiency partly ameliorated	SC	Supporting
M32	Zhang 2022 [26]	Randomized double-blind controlled trial, 8 FA dose arms 0.4–2.4 mg/d, n = 1,567	At 2.4 mg/d plasma SAH rose (β = 1.93, 95% CI 0.22–3.64); significant in CT (p = 0.027) and CT+TT (p = 0.005) but not in TT alone (p = 0.095); UMFA was not measured	RoB 2: Some concerns	Suggestive
M34	Martinez-Morata 2024 [79]	Metabolomics sub-study of an RCT (FA 800 μg/d vs placebo × 12 wk, n = 60)	UMFA associated with lower plasma arachidonic acid	SC	Supporting

Supplementary Table S2: Descriptive Evidence Profile, Human Observational Studies (n = 24). Observational evidence is appraised descriptively using per-study ROBINS-I [69] and OCEBM 2011 [42] levels with an indicative certainty judgement; these per-study judgements do not constitute an aggregate certainty rating for the observational evidence base, which is not the focused PICO question of this narrative review.

#	First Author, Year	Cohort / Setting	Key Finding	UMFA	ROBINS-I	Contribution	OCEBM
O1	Pfeiffer 2015 [18]	NHANES 2007–	UMFA	detectable	Mod	Supporting	4

#	First Author, Year	Cohort / Setting	Key UMFA Finding	ROBINS-I	Contribution	OCEBM
		2008, n = 2,707	(>0.3 nmol/L) in >95%; UMFA >1 nmol/L in 33.2% overall and 21.0% of fasting adults ≥20 y			
O2	Bailey 2020 [48]	NHANES 2011–2014, n = 2,420 aged ≥60	Low B12 with high UMFA and DSST <34, OR 2.16 (1.05–4.47)	Mod	Supporting	4
O3	Morris 2007 [30]	NHANES 1999–2002, n = 1,459 aged ≥60	High serum folate with low B12 and cognitive impairment, OR 2.6; UMFA not measured	Mod	Supporting	4
O4	Morris 2010 [47]	NHANES 1999–2002, n = 1,858 aged ≥60	UMFA detected in ~one third; detectable UMFA with low B12 and lower cognitive test scores	Mod	Supporting	4
O5	Plumptre 2015 [49]	Toronto pregnancy cohort, 368 women plus cord blood	UMFA detectable in 97% of maternal and 93% of cord plasma samples	Low	Supporting	2b
O8	Raghavan 2018 [32]	Boston Birth Cohort, 1,257 mother–child pairs	Very high maternal plasma folate (≥60.3 nmol/L) ~2.5× ASD risk; both folate and B12 extremes together ~17.6×	Mod	Supporting	2b
O9	Raghavan 2020 [33]	Boston Birth Cohort, n = 567 (92 ASD)	Cord UMFA Q4 vs Q1 aOR 2.26 (1.08–4.75); Black	Mod	Supporting	2b

#	First Author, Year	Cohort / Setting	Key UMFA Finding	ROBINS-I	Contribution	OCEBM
			children aOR 9.85 (2.53–38.31)			
O10	Schmidt 2019 [54]	MARBLES, 241 high-risk younger siblings	First-month prenatal vitamin use and lower ASD recurrence, aRR 0.50 (0.30–0.81); UMFA not measured	Mod	Supporting	2b/3b
O11	Husebye 2022 [44]	MoBa, 227 antiseizure-medication-exposed children of 203 women with epilepsy	Null: maternal plasma UMFA was not associated with autistic traits or language impairment	Mod	Neutral	2b
O12	Bjørk 2018 [56]	MoBa, 335 antiseizure-medication-exposed children	Absence of periconceptional folic acid and autistic traits, aOR 5.9 at 18 mo and 7.9 at 36 mo; UMFA not measured	Mod	Supporting	2b
O13	Geijsen 2020 [31]	FOCUS consortium, n = 2,024 (296 with detectable folic acid)	Among participants with detectable folic acid, recurrence HR 1.31 (1.02–1.58) per doubling	Mod	Supporting	2b
O15	Matejcic 2017 [100]	EPIC, 2,491 breast-cancer cases / 2,521 controls	Plasma folate and B12 were measured but UMFA was not; the alcohol interaction reported is for B12 (Q4 vs Q1 OR 1.26,	Mod	Suggestive	3b

#	First Author, Year	Cohort / Setting	Key UMFA Finding	ROBINS-I	Contribution	OCEBM
			p-trend 0.05)			
O16	Troen 2006 [23]	105 healthy postmenopausal women	UMFA detectable in 78% of fasting samples; NK cytotoxicity ~23% lower when UMFA was detectable ($p = 0.04$)	Mod	Supporting	4
O17	Hirsch 2009 [65]	Chile, national hospital-discharge rates 1992–96 vs 2001–04	Colon-cancer discharge rate ratios 2.6 (45–64 y) and 2.9 (65–79 y) after fortification; ecological design and UMFA not measured	Serious	Suggestive	4
O19	Pauwels 2017 [150]	MANOE, Belgium, 115 mother–infant pairs	*IGF2* DMR, *DNMT1*, *LEP* and *RXRA* methylation were examined; LINE-1 and UMFA were not measured	Mod	Suggestive	2b
O20	Frye 2013 [34]	93 children with ASD	FRAA positive in 75.3%	Mod	Supporting	3b
O22	Ramaekers 2007 [146]	25 children with early-onset low-functioning autism	CSF 5-MTHF low in 23/25, explained by blocking FR autoantibodies in 19/23; folinic acid normalized CSF 5-MTHF	Mod	Substantive	4
O24	Quadros 2018 [131]	Family-based series, New York: 82 ASD children, 53 siblings, 65 fathers, 70	FRAA positivity 76% in ASD children versus 29% in controls	Mod	Supporting	4

#	First Author, Year	Cohort / Setting	Key UMFA Finding	ROBINS-I	Contribution	OCEBM
		mothers, 52 controls				
O27	Obeid 2010 [151]	87 pregnant women at delivery plus 29 cord samples, Germany	Folic acid >0.20 nmol/L in 44% of women and 55% of cord samples, not explained by supplement use	Mod	Supporting	4
O28	Fazili 2014 [72]	Laboratory specimen-handling and stability experiment	Characterizes MeFox, a 5-methylTHF oxidation product distinct from folic acid, and specimen-handling effects on folate forms; not a population survey	Low	Substantive	n/a
O30	Bailey 2010 [46]	NHANES 2001–2002, n = 1,121 adults ≥60	UMFA detected in 38% of participants	Mod	Supporting	4
O31	Mason 2007 [66]	Ecological time-series, United States and Canada	Colorectal-cancer incidence 4–6 per 100,000 above the pre-fortification trend after 1996 (US) and 1998 (Canada); hypothesis paper with no participants	Serious	Suggestive	4
O35	Giorlandino 2025b [127]	Altamedica centre, Rome: 3,600 first-trimester scans → 27 with NT ≥3.5 mm → 11 followed to 36	4/4 offspring of FRAA-positive mothers developed ASD versus 1/7 of FRAA-negative	Mod	Suggestive	2b

#	First Author, Year	Cohort / Setting	Key UMFA Finding	ROBINS-I	Contribution	OCEBM
		mo				
O36	Sweeney 2009 [96]	50 unfasted blood donors; 20 fasted mothers with 20 paired cord samples, Ireland	UMFA present in 18/20 mothers (1.31% of total plasma folate), 17/20 cord samples and 49/50 blood donors (2.25%)	Mod	Supporting	4

Citation labels for the two Norwegian Mother, Father and Child Cohort (MoBa) entries were corrected against the reference list. The entry previously labelled "Wiens 2016" corresponded to Wiens & DeSoto (Brain Sci 2017;7:149) [67], which is a review and is therefore not an eligible primary observational contribution; it has been removed from this table and from the observational count, and is retained only as a contextual citation. The two eligible primary MoBa contributions are Husebye et al. 2022 [44], which measured maternal plasma UMFA directly (Class 1), and Bjørk et al. 2018 [56], which assessed periconceptional folic acid supplementation in antiepileptic-drug-exposed pregnancies (Class 3). The observational stream total is reconciled accordingly (24 analytic contributions).

Supplementary Table S3: Descriptive Evidence Profile, Interventional Trials (n = 32). Randomized trials are appraised descriptively using RoB 2.0 and non-randomized human interventions using ROBINS-I. Narrative reviews, systematic reviews, meta-analyses, and other secondary syntheses are not included in this table, in this count, or in any analysis, and were used only for citation chasing and contextual orientation. This applies without exception, including to pooled analyses of randomized trials: the pooled analysis of thirteen randomized folic acid trials by Vollset et al. [109] is the largest randomized safety dataset bearing on folic acid exposure and is discussed in the narrative on that basis, but it is a secondary synthesis and is therefore not counted as an analytic contribution (deviation D19). OCEBM 2011 [42] levels and the descriptive-contribution labels defined above are reported per contribution. The formal GRADE certainty rating for the focused FA-versus-(6S)-5-MTHF PICO question (plasma UMFA, RBC folate, plasma tHcy) is reported in the main manuscript Methods and applies only to Pools P1–P3, not to the table as a whole.

Note on pool eligibility. Trials contributing to the focused FA-versus-(6S)-5-MTHF synthesis are identified in Supplementary Tables S4–S5. Pool P1 (plasma UMFA) draws on I10 (Cochrane 2024 [83]), I11 (Troesch 2019 [82]), and I13 (Draicchio 2026 [84]). Pool P2 (erythrocyte folate) reuses only I10 and I11 (k = 2); the Cochrane/Karakochuk human-milk report is the same randomized pregnancy cohort as I10 and contributes neither erythrocyte folate nor plasma tHcy. Pool P3 (plasma tHcy) reuses I10 and includes Lamers 2006 [87]/companion Lamers 2004 [141] (k = 2). Houghton 2006 [88], listed as a third P3 contributor in the originally submitted version, has been excluded from the pool because its folic acid arm was a separately assigned reference group rather than a randomized comparator, so it does not satisfy the registered head-to-head randomized criterion; it is retained in Supplementary Table S5 as an excluded contrast with the reason stated. Draicchio contributes to P1 only because it did not measure erythrocyte folate or plasma tHcy. Thus, P1–P3 draw on four distinct trial cohorts, with the Cochrane cohort counted once despite multiple reports.

#	Trial	Comparator	Primary Outcome	RoB tool	Contribution	OCEBM
I1	Kelly 1997 [15]	Acute within-subject FA dose-escalation; randomization not stated	UMFA above ~266 μg/meal; >200 μg for single doses	SC	Substantive	2b
I2	Sweeney 2007 [16]	Non-randomized fixed-order dose-descending fortified bread	UMFA at 400 μg/d, not at 200 or 100 μg/d	SC	Substantive	2b
I3	Patanwala 2014 [20]	FA vs 5-formylTHF randomized crossover	Portal UMFA 80 ± 12%	Low	Substantive	1b
I4	Paniz 2017 [24]	5 mg/d FA × 90 d, single arm, uncontrolled	UMFA increased (96.6% detectable at day 45) while NK number and cytotoxicity fell	ROBINS-I High	Supporting	3b
I5	Tam 2012 [80]	1.1 vs 5 mg/d FA, randomized open-label	Detectable UMFA rose in both arms at wk 6 and 12 with no between-dose difference; tHcy not measured	SC	Supporting	2b
I6	Murphy 2021 (FACT ancillary) [43]	4.0 mg/d vs placebo on top of ≤1.1 mg/d in pregnancy (n = 19 vs 31)	Serum UMFA 4.6 vs 1.9 nmol/L (p = 0.008); erythrocyte folate unchanged	SC	Supporting	1b
I7	Pentieva 2016 [97]	400 μg/d vs placebo in pregnancy (n = 59 vs 67)	More FA-group women had detectable folic acid but	Low	Substantive	1b

#	Trial	Comparator	Primary Outcome	RoB tool	Contribution	OCEBM
			concentrations did not differ (p = 0.38); cord folic acid detectable in 8/53			
I8	Fleming 2024 [86]	400 vs 800 μg/d FA in pregnancy (n = 16 vs 12 completed)	Dose effect on maternal serum UMFA (p = 0.0225); no cord-blood difference; tHcy not measured	SC	Supporting	1b
I9	Sulistyoningrum 2024 [50]	Multivitamin ± 800 μg/d FA after 12 wk gestation (n = 46 vs 44)	UMFA 0.6 ± 0.7 vs 1.4 ± 2.7 nmol/L; adjusted difference −0.85 (−1.62, −0.08) nmol/L	Low	Substantive	1b
I10	Cochrane 2024 (maternal blood) [83]	FA vs (6S)-5-MTHF 0.6 mg/d in pregnancy; same parent trial as I14	Plasma UMFA 0.6 nmol/L higher (0.2, 1.1) with FA; erythrocyte and serum folate did not differ	Low	Substantive	1b
I11	Troesch 2019 [82]	FA vs L-5-MTHF infant formula	UMFA lower with L-5-MTHF (0.73 vs 1.15 nmol/L); erythrocyte folate higher; weight gain equivalent	RoB 2: High (plasma UMFA); Some concerns (erythrocyte folate) ‡	Substantive	1b
I12	Obeid 2024 [85]	400 vs 800 μg/d 1:1 FA/(6S)-5-MTHF mixture,	Higher serum (6S)-5-MTHF at 800 μg but no	RoB 2: Some concerns	Supporting	1b

#	Trial	Comparator	Primary Outcome	RoB tool	Contribution	OCEBM
		open-label	higher prevalence of detectable UMFA (23.6% vs 13.3%, p = 0.116)			
I13	Draicchio 2026 [84]	FA vs (6S)-5-MTHF in pregnancy, 24 wk	Detectable maternal UMFA 7% vs 31%; both placental samples with detectable UMFA were from the FA arm	RoB 2: High §	Substantive	1b
I14	Cochrane 2023 (human milk) [81]	FA vs (6S)-5-MTHF in pregnancy; same parent trial as I10	Median milk UMFA 11 nmol/L higher (6.4–17) with FA; 28% vs 2% of total milk folate	Low	Supporting	1b
I15	Obeid 2011 [114]	FA 5 mg + B6 40 mg + B12 2 mg vs placebo × 3 wk, n = 74 (median age 82)	UMFA rose from median 0.08 to 15.3 nmol/L and tHcy fell from 17.2 to 9.0 μmol/L; the active arm was a combined regimen, so the effect is not attributable to folic acid alone	Mod	Supporting	1b
I16	Obeid 2016 [113]	400 μg/d FA (n = 31) vs FA + B12 + B6 (n = 27), single-blind, no	Higher UMFA prevalence in the FA-only arm; tHcy fell	RoB 2: Some concerns	Substantive	1b

#	Trial	Comparator	Primary Outcome	RoB tool	Contribution	OCEBM
		placebo	similarly in both (between-arm p = 0.66)			
I17	Miyaki 2005 [90]	FA 1 mg/d vs placebo, randomized within each MTHFR C677T stratum	tHcy fell in all three genotypes, most in TT; significant linear trend with T-allele number	Low	Supporting	1b
I18	HOPE-2 2006 [115]	FA + B-vitamins vs placebo, CV outcomes	Primary events 18.8% vs 19.8% (RR 0.95, 0.84–1.07); UMFA not measured	Low	Suggestive	1b
I19	VITATOPS 2010 [116]	FA + B-vitamins after TIA or stroke	Composite 15% vs 17% (RR 0.91, 0.82–1.00); UMFA not measured	Low	Suggestive	1b
I20	VISP 2004 [117]	High- vs low-dose B-vitamin formulation after stroke	No treatment effect on any endpoint (RR 1.0, 0.8–1.1); comparator was low-dose vitamins, not placebo; UMFA not measured	Low	Suggestive	1b
I21	FACIT 2007 [118]	800 μg/d FA × 3 y, cognition as a secondary endpoint	Memory 0.132 (0.032–0.233) and information-processing speed 0.087 (0.016–0.158) improved; UMFA not measured	Low	Suggestive	1b

#	Trial	Comparator	Primary Outcome	RoB tool	Contribution	OCEBM
I22	VITACOG / de Jager 2012 [119]	FA 0.8 mg + B12 0.5 mg + B6 20 mg vs placebo × 2 y in MCI	tHcy 30% lower; executive function (CLOX) stabilized (p = 0.015); UMFA not measured	Low	Suggestive	1b
I23	CSPPT 2015 [120]	FA + enalapril vs enalapril alone, stratified by MTHFR C677T	First stroke 2.7% vs 3.4% (HR 0.79, 0.68–0.93); UMFA not measured	Low	Suggestive	1b
I24	Zhang 2022 [26]	8 FA dose arms 0.4–2.4 mg/d plus enalapril vs enalapril alone	Primary outcome was change in plasma SAH; tHcy was measured at baseline only and as a covariate	Low	Supporting	1b
I25	Frye 2018 [36]	Folinic acid vs placebo in ASD with language impairment	Verbal communication 5.7 (1.0, 10.4) standardized points, d = 0.70	Low	Substantive	1b
I26	Batebi 2021 [38]	Folinic acid adjunct to risperidone in ASD, n = 55	Time × treatment effect on inappropriate speech (p = 0.044), stereotypy (p = 0.036), hyperactivity (p = 0.003)	Low	Supporting	1b
I27	Renard 2020 (EFFET) [39]	Folinic acid 5 mg twice daily vs placebo × 12 wk,	ADOS global improved within the	RoB 2: Some concerns	Suggestive	2b

#	Trial	Comparator	Primary Outcome	RoB tool	Contribution	OCEBM
		n = 19	folinic acid arm (p = 0.003) with no significant placebo change; no between-group p-value was reported			
I28	Zhang 2025 [40]	Folinic acid + TEACCH rehabilitation (n = 49) vs rehabilitation alone (n = 27), open-label	PEP-3 social-reciprocity difference 1.20 (0.29–2.10), p = 0.010	RoB 2: Some concerns	Substantive	1b
I29	Giorlandino 2025a [126]	Folinic acid preconception through gestation; dose not stated in the report	Offspring neurotypical at 3 y (case series, n = 2)	ROBINS-I: Critical ¶	Supporting	4
I30	Giorlandino 2026 [126] (FRAA-FOL pilot) [128]	Leucovorin 0.5 mg twice daily vs FA 0.4 mg/d in FRAA-positive pregnancy (ISRCTN96269039); 29 randomized, 18 completed	Offspring ASD 1/10 vs 5/8 (p = 0.043)	RoB 2: Some concerns	Supporting	2b
I31	Martinez-Morata 2024 [79]	Metabolomics substudy of the Folic Acid and Creatine Trial, Bangladesh (a different trial from I6)	UMFA associated with lower plasma arachidonic acid	Low	Supporting	1b
I32	Figueiredo 2009 (AFPPS) [57]	FA 1 mg/d vs placebo, 643 men;	Prostate cancer HR 2.63 (1.23–	Low	Suggestive	1b

#	Trial	Comparator	Primary Outcome	RoB tool	Contribution	OCEBM
		contained within the Vollset et al. pooled analysis [109]	5.65); UMFA not measured			

† The entry previously numbered I27 (Rossignol & Frye 2021 [37]) is a systematic review. Consistent with the eligibility rule that reviews and other secondary syntheses are used for citation chasing only, it has been removed from this table and from the interventional count. The individual primary trials it summarizes are listed separately where they met eligibility criteria. One entry was transferred into this table from Supplementary Table S2 during the source-verification re-extraction (deviation D17) because the cited publication reports a randomized allocation of folic acid and therefore does not belong in an observational table: I32 (Figueiredo 2009 [57], the placebo-controlled Aspirin/Folate Polyp Prevention Study, previously O14). The entry previously numbered O32 (Vollset 2013 [109]) was likewise found to be randomized rather than observational, but it is a pooled analysis of thirteen trials rather than a primary trial; under the eligibility rule stated in Section 2.2 of the main text it has been withdrawn from the ledger entirely and is retained only as a contextual citation [109] (deviation D19). The interventional count is reconciled to 32 contributions. Full row-level disposition is given in Supplementary Table S10.

*‡ I11 (Troesch 2019 [82]) was appraised with RoB 2, and the overall judgement is outcome-specific. Domain judgements: randomization process — some concerns (randomized and double-blind, but the per-protocol groups differed at baseline in age, p = 0.0056, and in MTHFR A1289C distribution, p = 0.0168); deviations from intended interventions — some concerns (the primary analysis was per protocol, restricted to infants completing all visits without protocol deviations, with a concordant modified intention-to-treat analysis reported confirmatorily); missing outcome data — low for erythrocyte folate (238 of 244 per-protocol infants analysed) but **high for plasma UMFA**, which was analysed in only 124 of 244 per-protocol infants because many samples fell below the limit of quantification; measurement of the outcome — low (blinded assessors, LC-MS/MS); selection of the reported result — some concerns (no analysis plan was available for the folate-form biomarkers). Overall: **high risk of bias for plasma UMFA** and **some concerns for erythrocyte folate**.*

*§ I13 (Draicchio 2026 [84]) was appraised with RoB 2. Domain judgements: randomization process — some concerns (randomized, but baseline imbalance between arms is reported); deviations from intended interventions — **high** (the only reported analysis was per protocol, 31 of 40 randomized participants per arm, and no intention-to-treat analysis was reported); missing outcome data — some concerns (22.5% post-randomization exclusion per arm, with incompletely characterized reasons); measurement of the outcome — low (LC-MS/MS); selection of the reported result — some concerns. Overall: **high risk of bias**, driven by the per-protocol-only analysis. The non-equimolar comparator (approximately 588 μg (6S)-5-MTHF versus 800 μg folic acid) is treated as an indirectness and applicability limitation rather than as a risk-of-bias domain, and is the reason this trial is removed in the prespecified-criterion equimolar-only sensitivity analysis.*

*¶ I29 (Giorlandino 2025a) is a non-randomized case series (n = 2) and was appraised with ROBINS-I rather than RoB 2, consistent with the design-tailored appraisal rule in Section 2.6 of the main text. Domain judgements: confounding — **critical** (no comparator group, so no confounding control is possible); selection of participants — serious; classification of interventions — low; deviations from intended interventions — moderate; missing data — low; measurement of outcomes*

— serious (unblinded outcome ascertainment by the treating clinicians); selection of the reported result — serious (reported outcomes were favourable and no protocol was available). Overall: ***critical risk of bias****. This contribution is descriptive only and contributes to no pool.*

Supplementary Table S4 — Pool P1 study-level inputs (plasma UMFA, FA vs (6S)-5-MTHF head-to-head trials in the retrieved evidence set).

Trial	Population	Dose	Duration	n (FA / 5-MTHF)	Assay	Hedges' g [95% CI]
Troesch 2019 [82] ‡	Infants	Equivalent to AI	4 mo	42 / 36 assumed for UMFA ‡ (120 / 120 randomized)	LC-MS/MS	+1.12 [+0.64, +1.60]
Cochrane 2024 [83]	Pregnancy	0.6 mg/d	16 wk	25 / 29 at endline § (30 / 30 randomized)	LC-MS/MS	+1.26 [+0.67, +1.84]
Draicchio 2026 [84] †	Pregnancy	1,330 μg DFE FA vs 1,000 μg DFE 6S-5-MTHF (within prenatal MVI)	24 wk	31 / 31 completers	LC-MS/MS	+0.64 [+0.13, +1.15]
Obeid 2024 [85] (excluded from Pool P1)	Non-pregnant women	400 vs 800 μg/d 1:1 FA/(6S)-5-MTHF mixture	8 wk	85 / 87	LC-MS/MS	Excluded: not a head-to-head FA vs 5-MTHF contrast
**Pooled (DL + HKSJ)**						+0.99 [+0.20, +1.79]; p = 0.033; I^2 = 30%; τ^2 = 0.031

† Declared post-registration amendment: Draicchio 2026 [84] (Front Nutr 2026;13:1679067; NCT05673070) is retained in Pool P1 only despite a non-equimolar multivitamin contrast: approximately 588 µg (6S)-5-MTHF versus 800 µg FA (approximately 40% more folate on a molar basis in the FA arm). The products also differed in other B vitamins, choline, and vitamin D. The analysis was per protocol (31/31 completers of 40/40 randomized); no intention-to-treat analysis was reported. Prespecified-criterion equimolar-only sensitivity analysis excluding Draicchio (leave-one-out prespecified in the registered protocol as an analysis type, applied at the registered equimolar-dose criterion): Hedges' g +1.17 [+0.32, +2.03], p = 0.036, I^2 = 0%, k = 2. The two remaining equimolar trials are directionally concordant, heterogeneity is eliminated,

and the interval excludes the null, so the registered effect does not depend on retaining the non-equimolar trial. The interval remains wide and its lower bound is compatible with a small effect.

‡ Analysed denominator for Troesch 2019 [82] — an explicit assumption, not a recovered quantity. Plasma UMFA at visit 4 was analysed in 124 of the 244 per-protocol infants (Troesch et al. 2019 [82], Table 3), because many samples fell below the limit of quantification (LOQ); the source reports only this pooled total and does not state how the 124 quantifiable observations were distributed across the three groups. The per-protocol group sizes for the trial as a whole are separately derivable from the reported MTHFR C677T genotype percentages against the stated total of n = 238 (the only integer denominators consistent with those percentages and summing to 238 are 69 L-5-MTHF, 81 folic acid, and 88 breastfed reference), but applying that ratio to the 124 UMFA observations is an assumption and not an arithmetic consequence, and it is a conservative one: because missingness was driven by values falling below the LOQ, and the L-5-MTHF arm had systematically lower UMFA, that arm is expected to have retained proportionally fewer quantifiable observations than a proportional split implies. The analysis therefore reports 42 (folic acid) / 36 (L-5-MTHF) as an assumed allocation and quantifies the consequences of that assumption in Supplementary Table S4b. The pooled Pool P1 estimate varies only between +0.96 and +1.00 across the full range of allocations tested, so the registered conclusion does not depend on it.

§ Analysed denominator for Cochrane 2024 [83]. Table 3 of the source states that n = 54 participants were included for all endline analyses (60 completed baseline visits, 54 completed endline visits, a 90% retention rate), distributed as 29 (6S)-5-MTHF and 25 folic acid. The originally submitted analysis used the randomized allocation of 30 / 30, which is corrected here; the same n = 54 endline denominator is used for erythrocyte folate in Supplementary Table S5, so the two tables are now consistent for this trial.

Effect-size derivation (deviation D13). Both median-reported trials were re-derived for this revision using Wan et al. 2014 [77] Method 3 exactly as specified in Supplementary Methods Section 1: mean = (Q1 + median + Q3)/3 and SD = (Q3 − Q1)/$\eta(n)$ with $\eta(n) = 2\cdot\Phi^{-1}((0.75n - 0.125)/(n + 0.25))$. The originally submitted values (Troesch +1.33 [+0.84, +1.82]; Cochrane +1.05 [+0.50, +1.60]) had been computed with the median substituted directly for the mean and the standard deviation approximated as IQR/1.35, which is the asymptotic constant rather than the sample-size-dependent $\eta(n)$. Correcting the estimator moves the two trials closer together (+1.12 and +1.26), which is why heterogeneity falls from I^2 = 46% to I^2 = 30% and τ^2 from 0.058 to 0.031. The pooled estimate is essentially unchanged (+1.01 to +0.99), but the equimolar-only sensitivity analysis now excludes the null; the GRADE imprecision downgrade for Pool P1 has been re-derived accordingly (Supplementary Table S8).

Supplementary Table S4b — Pool P1 sensitivity analyses for the assumed per-arm allocation of the 124 quantifiable plasma-UMFA observations in Troesch 2019 [82], and for the limit-of-quantification censoring that produced them.

Panel A — allocation sensitivity. The source reports 124 quantifiable UMFA observations at visit 4 across three groups without an arm-specific breakdown. The reference analysis assumes 42 folic acid / 36 L-5-MTHF, obtained by apportioning 124 in the ratio of the derived per-protocol group sizes (81 / 69 / 88) and assigning 46 to the breastfed reference group. Panel A varies the folic acid / L-5-MTHF split across the full plausible range while holding the reference group at 46, and recomputes the trial estimate, the three-trial Pool P1 estimate, and the prespecified-criterion equimolar-only estimate.

Assumed FA / 5-MTHF split	Troesch Hedges' g [95% CI]	Pool P1, k = 3 [95% HKSJ CI]	Equimolar-only, k = 2 [95% HKSJ CI]
26 / 52	+1.14 [+0.63, +1.64]	+1.00 [+0.18, +1.81]	+1.19 [+0.46, +1.92]
36 / 42	+1.13 [+0.65, +1.61]	+1.00 [+0.20, +1.79]	+1.18 [+0.37, +1.98]
42 / 36 **(reference)**	+1.12 [+0.64, +1.60]	+0.99 [+0.20, +1.79]	+1.17 [+0.32, +2.03]
48 / 30	+1.11 [+0.62, +1.60]	+0.99 [+0.20, +1.78]	+1.17 [+0.26, +2.07]
58 / 20	+1.10 [+0.56, +1.64]	+0.98 [+0.18, +1.78]	+1.17 [+0.17, +2.17]
68 / 10	+1.09 [+0.40, +1.77]	+0.96 [+0.13, +1.79]	+1.18 [+0.12, +2.25]

Every allocation tested yields a Pool P1 estimate between +0.96 and +1.00 with a 95% HKSJ interval excluding the null, and an equimolar-only estimate between +1.17 and +1.19. The registered conclusion for plasma UMFA is therefore insensitive to the allocation assumption across its entire plausible range. Precision, not direction or magnitude, is what the assumption affects: the lower bound of the three-trial interval falls from +0.20 to +0.13 at the most extreme allocation tested, and the equimolar-only lower bound from +0.32 to +0.12.

Panel B — limit-of-quantification censoring. Because the 124 quantifiable observations are those exceeding the assay LOQ, the missingness is outcome-dependent rather than random, and the direction of the resulting bias can be established. Values were simulated from lognormal arm distributions calibrated to the reported medians (folic acid 1.15, L-5-MTHF 0.73 nmol/L) across a range of dispersions, censored at the reported LOQ of 0.53 nmol/L, and the Wan Method 3 estimator was applied to the censored samples in the same way as to the observed data (4,000 replicates per scenario).

Dispersion (log-scale SD)	Samples retained above LOQ	True Hedges' g	g estimated after censoring	Bias
0.45	85.9%	+0.98	+0.81	−0.18
0.60	80.2%	+0.74	+0.58	−0.17
0.75	75.7%	+0.60	+0.45	−0.15

LOQ censoring biases the estimated standardized mean difference toward the null, by approximately 0.15 to 0.18 across the scenarios tested. The mechanism is that the low-UMFA L-5-MTHF arm loses proportionally more observations to censoring, which raises its apparent mean and compresses the between-arm contrast. The censoring in Troesch 2019 [82] therefore works against the direction reported in Pool P1 rather than generating it, and the reported estimate for that trial is, if anything, conservative. This analysis also motivates the statement in the Table S4 footnote that a proportional allocation is conservative, since the same mechanism implies the L-5-MTHF arm retained fewer than 36 of the 124 observations.

Both panels are post-registration analyses added in revision in response to peer review; they are declared as deviation D14 in Supplementary Methods Section 5. Neither panel alters any registered conclusion.

Supplementary Table S5 — Pool P2 (erythrocyte folate) and Pool P3 (plasma total homocysteine) study-level inputs.

Pool (outcome, scale)	Trial	Study-level estimate
P2 (erythrocyte folate, SMD)	Troesch 2019 [82]	907.0 ± 192.8 (n = 69) vs 839.4 ± 142.4 (n = 81) nmol/L; Hedges' g = +0.40 [+0.08, +0.73] †
	Cochrane 2024 [83]	1826 ± 471 (n = 29) vs 1998 ± 421 (n = 25) nmol/L; Hedges' g = −0.38 [−0.92, +0.16] †
	**Not pooled, k = 2**	Q = 5.89; I^2 = 83%; directionally discordant trials; no pooled estimate ‡
P3 (plasma tHcy, µmol/L)	Cochrane 2024 [83]	Randomized, equimolar, intention-to-treat; endline/postpartum tHcy values not retrievable §
	Lamers 2006 [87]	No tHcy in the 2006 report; companion Lamers et al. 2004 [141] reports time × treatment p < 0.001 and no difference among supplemented arms (p > 0.05) ¶
	Houghton 2006 [88] (excluded)	Week-16 geometric means (95% CI): 5-MTHF 8.1 [7.4, 9.0], FA 8.7 [8.0, 9.4], placebo 8.4 [7.7, 9.2] µmol/L; FA reference arm non-randomized #
	**Not pooled, k = 2**	Pooling abandoned: neither contributing report supplies recoverable per-arm arithmetic means with SDs at the outcome time point. Qualitative finding: neither report detected a statistically significant between-form difference in tHcy; all reported concentrations remained below 13 µmol/L **

† *Per-arm analysed n were recovered from the source publications and are no longer assumed.* ***Troesch 2019 [82]:*** *the per-protocol group sizes are not stated numerically, but the reported MTHFR C677T genotype percentages are consistent with only one set of integer denominators summing to the stated total of n = 238 — 69 (L-5-MTHF), 81 (folic acid), and 88 (breastfed reference). This derivation is corroborated by the reported between-group p = 0.0095 for erythrocyte folate, which is consistent with approximately 150 participants in the two randomized arms and not with the 238 previously assumed.* ***Cochrane 2024 [83]:*** *60 participants were randomized 30/30, and 54 completed the endline visit; the participant-flow caption states that the folic-acid group contributed n 19 at the postpartum visit, comprising n 18 who attended endline plus n 1 who delivered prematurely and did not attend endline, and that n 7 endline participants declined the postpartum draw, giving 25 analysed at endline in the folic-acid arm and therefore 29 in the (6S)-5-MTHF arm. Recomputing with these denominators changes the Troesch interval from +0.40 [+0.14, +0.65] to +0.40 [+0.08, +0.73] and leaves the Cochrane estimate unchanged at −0.38 [−0.92, +0.16].*

‡ *Pool P2 is not pooled: the two trials are statistically and directionally discordant (Q = 5.89; I^2 = 83%), and the two-study Hartung–Knapp interval is uninformative. The evidence is insufficient to support an equivalence or non-inferiority claim for erythrocyte folate.*

§ Cochrane 2024 [83] is the only randomized, equimolar, intention-to-treat head-to-head tHcy contrast. Endline and postpartum tHcy were reported only in an online supplementary table that could not be retrieved; no effect estimate was constructed.

¶ The Lamers 2006 [87] publication reports no tHcy. The companion report is Lamers Y, Prinz-Langenohl R, Moser R, Pietrzik K. Am J Clin Nutr 2004;79:473–8 [141]; per-arm means and SDs were not recoverable, so no effect estimate was constructed.

# Houghton 2006 [88] is excluded from Pool P3. Only 5-MTHF versus placebo was randomized; the FA arm was a separately assigned, non-randomized reference group, so the folate-form contrast is not a randomized comparison and does not meet the registered eligibility criterion. tHcy was reported as geometric means with 95% CIs, not arithmetic means with SDs; no effect estimate was constructed.

*** Pool P3 is reported without a pooled estimate. Because neither of the two eligible contributing reports supplies the per-arm arithmetic means and standard deviations that a mean-difference synthesis requires, any pooled value would rest on imputed distributional form and imputed variance. The withholding rule stated in Section 2.8 of the main text (declared as deviation D11) therefore applies, and the registered tHcy outcome is reported qualitatively. The evidence is insufficient to support an equivalence or non-inferiority claim for plasma tHcy.*

Supplementary Table S6 — Cord-blood UMFA × ASD evidence from the retrieved cohort source (single cohort; no meta-analysis).

Cohort	Sample	Comparison	Adjusted OR [95% CI]
Boston Birth Cohort	n = 567 (92 ASD cases)	Q4 vs Q1 cord UMFA	2.26 [1.08, 4.75]
MARBLES	No cord-UMFA category contrast in the retrieved evidence	Not pooled	Not applicable
EARLI	No cord-UMFA category contrast in the retrieved evidence	Not pooled	Not applicable
No meta-analysis performed			Single eligible retrieved cohort

Supplementary Table S7 — Pool S3 (high-dose vs standard FA, UMFA, nmol/L) and Pool S4 (MTHFR × FA, tHcy) study-level inputs.

Pool (outcome, unit)	Trial or stratum	Comparison	n (high / low)	Estimate
S3 (UMFA, nmol/L)	Murphy/FACT 2021 [43]	1 vs 4–5 mg/d, pregnancy	19 / 31	+11.3
	Tam 2012 [80] †	1 vs 5 mg/d	19 / 19	−16.1
	Fleming 2024 [86]	400 vs 800 µg/d,	12 / 16	+1.0

Pool (outcome, unit)	Trial or stratum	Comparison	n (high / low)	Estimate
		pregnancy		
	Pooled (HKSJ), k = 3	High − low FA arm	—	−1.2 [−33.7, +31.4]; I^2 = 85%
	Sensitivity: Tam 2012 [80] excluded, k = 2	High − low FA arm	—	+4.6 [−58.0, +67.2]
S4 (tHcy, μmol/L) ‖	Miyaki 2005 [90] — CC	FA 1 mg/d vs placebo, 3 mo	35 / 34	−2.15 [−3.02, −1.28]
	Miyaki 2005 [90] — CT	FA 1 mg/d vs placebo, 3 mo	50 / 50	−2.02 [−2.55, −1.49]
	Miyaki 2005 [90] — TT	FA 1 mg/d vs placebo, 3 mo	17 / 17	−11.71 [−20.39, −3.03] ¤
	CSPPT / Huang 2018 [144] — CC	FA 0.8 mg/d vs none, ≈4.5 y	2238 / 2204	−0.99 [−1.21, −0.76]
	CSPPT / Huang 2018 [144] — CT	FA 0.8 mg/d vs none, ≈4.5 y	4029 / 4024	−1.31 [−1.49, −1.12]
	CSPPT / Huang 2018 [144] — TT	FA 0.8 mg/d vs none, ≈4.5 y	1951 / 1967	−3.27 [−3.84, −2.70]
	PFAT-Hcy 2024 [145] — CC	FA 0.8 mg/d vs 0 mg, 8 wk	—	−2.8 §§
	PFAT-Hcy 2024 [145] — CT	FA 0.8 mg/d vs 0 mg, 8 wk	—	−1.5 §§
	PFAT-Hcy 2024 [145] — TT	FA 0.8 mg/d vs 0 mg, 8 wk	—	−5.1 §§
	**Not pooled, k = 3**	—	—	Monotone gradient with 677T allele count in all three trials; ≈1.0 to 1.2 μmol/L additional tHcy reduction per T allele in the two trials with derivable intervals ‖

† Tam 2012 [80] was converted to the mean scale using the Wan range-based estimator; the source paper reports z = −0.11, P = 0.91 at Week 12, and the negative point estimate is driven by a single 86.4 nmol/L low-dose outlier. The three Pool S3 trials are not directionally concordant at the point-estimate level, so the pooled estimate is reported as directionally null and hypothesis-generating; the trial-level dose–response argument is

anchored on the positive point estimates in Murphy/FACT 2021 [43] and Fleming 2024 [86] and on the per-occasion pharmacology (10,11). ‖ **Pool S4 was reconstructed in revision and is no longer pooled (deviation D16).** As originally submitted, Pool S4 reported pooled genotype-specific estimates of −0.99 [−1.42, −0.56] (CC), −1.26 [−1.61, −0.91] (CT) and −1.90 [−2.41, −1.39] (TT) with a linear trend of −0.46 μmol/L per 677T allele, attributed to Huo 2015 [120], Zhang 2022 [26] and Miyaki 2005 [90]. Source re-verification in response to peer review established that this attribution could not be sustained. Huo 2015 [120] is the CSPPT primary stroke-outcome report; it uses MTHFR C677T as a randomization stratifier and reports genotype-stratified serum folate, but no genotype-stratified change in tHcy. Zhang 2022 [121] specifies change in S-adenosylhomocysteine as its primary outcome and reports tHcy only as a baseline characteristic and model covariate, so it contributes no tHcy estimate; it is retained elsewhere in this Review for the SAH finding it does report. Only Miyaki 2005 [90] of the three cited reports supplies genotype-stratified tHcy change. The previously reported pooled estimates and the −0.46 μmol/L per-allele trend have therefore been withdrawn in full and are not reported anywhere in this revision. Pool S4 has been rebuilt from the three randomized reports that do supply the outcome, each entered once: Miyaki 2005 [90], the CSPPT genotype-stratified post hoc analysis [144] (which supersedes [120] for this outcome, the two being the same trial population), and PFAT-Hcy 2024 [145]. Because these trials differ in population, dose and duration to a degree that makes a single pooled estimate uninterpretable, no pooled estimate is reported, under the same rule applied to Pools P2 and P3 (deviation D11). Miyaki estimates are placebo-corrected between-group differences computed from the printed Table 2 means and SDs, with $SE = \sqrt{(SD_1^2/n_1 + SD_2^2/n_2)}$; CSPPT values are the adjusted between-group differences printed in [144]. ¤ The Miyaki TT estimate is reported for completeness but is not interpretable at face value: the TT placebo arm rose by +4.87 μmol/L with an SD of 16.2 in n = 17, so the difference is dominated by placebo-arm variability. The corresponding 1-month value is −4.52 [−9.02, −0.02]. §§ PFAT-Hcy 2024 [83] prints arm-wise change with 95% CIs but not the between-arm difference; the values shown are differences computed from the printed arm means, and no interval is derivable without additional assumptions, so none is given. Corresponding values at the highest dose tested (2.4 mg/d) are −2.6 (CC), −2.0 (CT) and −8.0 (TT) μmol/L.

Supplementary Table S8 — GRADE Summary-of-Findings (focused FA-versus-(6S)-5-MTHF PICO)

This is the **only** table in the supplementary package that reports a formal GRADE certainty rating. GRADE is applied here to the focused PICO question — whether substitution of (6S)-5-methyltetrahydrofolate for synthetic folic acid alters plasma unmetabolized folic acid (UMFA), red-blood-cell folate, or plasma total homocysteine in head-to-head randomized comparisons — and is not extended to mechanistic, observational, or exploratory pool evidence reported elsewhere in this Review.

Outcome (Pool)	Studies (k)	Effect / finding	Certainty (GRADE)
Plasma UMFA (Pool P1)	3	Hedges' g +0.99 [+0.20, +1.79]; p = 0.033; I^2 = 30%; τ^2 = 0.031	⨁⨁○○ **Low**
Red-blood-cell folate (Pool P2)	2	Not pooled; +0.40 [+0.08, +0.73] and −0.38 [−0.92, +0.16]; I^2 = 83%	⨁○○○ **Very low**
Plasma total	2	Not pooled; no report detected a statistically	⨁○○○ **Very low**

Outcome (Pool)	Studies (k)	Effect / finding	Certainty (GRADE)
homocysteine (Pool P3)		significant between-form difference; all reported values below 13 μmol/L	

No patient-important clinical outcome appears in this table because none was eligible: no head-to-head trial of folate forms reported a clinical outcome, so clinical benefit and harm were not graded and no certainty rating for them is offered anywhere in this Review. All three ratings below describe biochemical outcomes. Certainty for plasma total homocysteine was downgraded twice for very serious imprecision — no pooled estimate is derivable, because no contributing report supplies recoverable per-arm means and standard deviations, and no non-inferiority margin was prespecified — and once for risk of bias, arising from incomplete outcome reporting in both contributing reports — endline values available only in an unretrievable online supplement in Cochrane 2024 [83], and no recoverable per-arm variances in Lamers 2006 [87] and its companion report. Neither the erythrocyte-folate nor the homocysteine finding supports an equivalence or non-inferiority claim.

GRADE domain judgements. Pool P1 (UMFA): downgraded once for serious risk of bias (two of the three contributing trials are at high risk of bias for the UMFA outcome — Troesch 2019 [82] because plasma UMFA was analysed in only 124 of 244 per-protocol infants, and Draicchio 2026 [84] because the only reported analysis was per protocol) and once for serious imprecision (k = 3; the pooled interval extends from a small to a very large effect on a combined analysed sample of fewer than 200 participants for this outcome, and its lower bound is additionally conditional on an assumed per-arm allocation in the largest trial, falling from +0.20 to +0.13 across the allocations tested in Supplementary Table S4b). Following correction of the effect-size derivation (deviation D13) the prespecified-criterion equimolar-only sensitivity analysis excludes the null and is therefore no longer cited in support of the imprecision downgrade, which rests on interval width and the fragility of the lower bound rather than on non-significance. Not downgraded for inconsistency (I^2 = 30%, eliminated when the non-equimolar trial is removed). Not downgraded for indirectness with respect to the registered analyte outcome, with one qualification stated explicitly: Draicchio 2026 [84] delivered a non-equimolar, multi-nutrient intervention and is indirect relative to the registered equimolar comparison. Rather than resolve this by aggregate judgement, the prespecified equimolar-only analysis provides the estimate restricted to directly relevant evidence (+1.17 [+0.32, +2.03]; k = 2), concordant with the three-trial pool. Had indirectness been rated serious for the body of evidence as a whole, Pool P1 would fall to very low. Low certainty concerns lower circulating UMFA, not clinical benefit. **Pool P2 (erythrocyte folate):** downgraded once for serious inconsistency (directionally opposed estimates; I^2 = 83%) and twice for very serious imprecision (k = 2; no pooled estimate is derivable, the two trial intervals point in opposite directions, the combined analysed sample is 204 participants, and no non-inferiority margin was prespecified), giving three downgrades from an initial rating of high and therefore very-low certainty. The arithmetic is set out explicitly because one single-level downgrade for inconsistency combined with one single-level downgrade for imprecision would yield low, not very low; the second imprecision level is justified on the grounds stated and not assumed (correction made in revision, deviation D15). **Pool P3 (tHcy):** downgraded twice for very serious imprecision (no pooled estimate is derivable from any contributing report, and no non-inferiority margin was prespecified) and once for risk of bias arising from incomplete outcome reporting in both contributing reports (Cochrane 2024 [83], endline and postpartum values available only in an online supplement that could not be retrieved; Lamers 2006 [87] and companion Lamers 2004 [141], no recoverable per-arm variances), yielding very-low certainty.

Houghton 2006 [88] was removed from this pool in revision because its folic-acid arm was not randomized (deviation D18), so it no longer contributes to any domain judgement here. Publication bias could not be formally assessed because no pool contained ten studies; funnel plots and Egger's test were not performed.

Interpretation. Substitution of (6S)-5-MTHF for FA appears to reduce circulating UMFA. Erythrocyte-folate and tHcy evidence cannot support an equivalence or non-inferiority claim: erythrocyte-folate findings are directionally discordant and not pooled, and the tHcy synthesis has no pooled estimate. No minimal clinically important difference has been established for plasma tHcy; absence of a detected difference must not be interpreted as clinical equivalence.

Supplementary Figure S1 — Pool S1 forest plot (plasma 5-MTHF, FA vs (6S)-5-MTHF). Random-effects HKSJ-adjusted SMD = +0.48 (95% CI -0.80 to +1.76), $k = 3$.

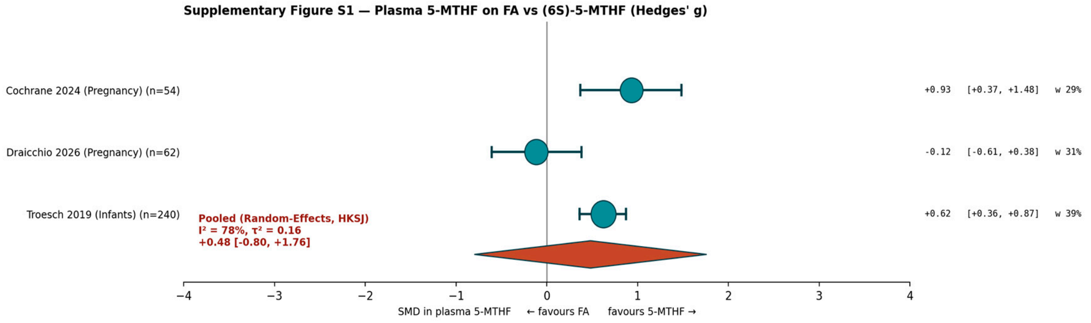

Supplementary Figure S1 — plasma 5-MTHF (FA vs 5-MTHF)

Supplementary Figure S2 — Cord-blood UMFA quartile 4 versus quartile 1 and ASD risk in the Boston Birth Cohort. No meta-analysis was performed and no pooled estimate is shown; the figure was regenerated in revision to remove a pooled diamond that contradicted that statement. The Boston Birth Cohort (n = 567, 92 ASD cases) is the only retrieved cohort providing a comparable cord-blood UMFA quartile contrast for ASD (deviation D5), with an adjusted OR of 2.26 (95% CI 1.08–4.75) as printed in the source. Author-generated using Matplotlib; not adapted from any published figure.

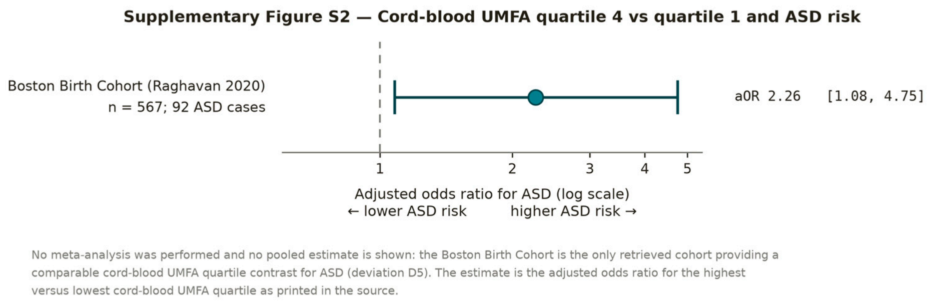

Supplementary Figure S2 — cord UMFA × ASD

Supplementary Figure S3 — Pool S3 forest plot (high-dose vs standard FA, raw mean difference UMFA, nmol/L). Random-effects HKSJ pooled MD = −1.2 nmol/L (95% CI −33.7 to +31.4), $I^2 = 85\%$, k = 3 (one secondary FACT-cohort 2024 [86] entry removed as duplicate of Murphy/FACT 2021 [43]; see Supplementary Table S7). Pool-level estimate is directionally null; per-trial mean differences were Murphy/FACT 2021 [43] +11.3,

Tam 2012 [80] −16.1 (driven by a single outlier; primary paper reports no significant between-dose difference), and Fleming 2024 [86] +1.0 nmol/L. A post-hoc exploratory sensitivity analysis excluding Tam 2012 [80] is shown in Supplementary Figure S3b.

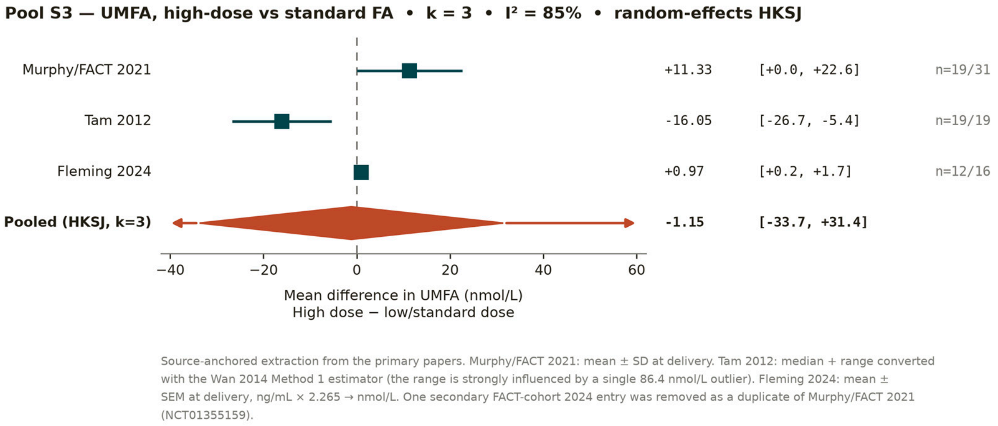

Supplementary Figure S3 — high-dose vs standard FA on UMFA

Supplementary Figure S3b — Pool S3 sensitivity analysis (Tam 2012 [80] excluded), high-dose vs standard FA, raw mean difference UMFA, nmol/L. Random-effects HKSJ pooled MD = +4.6 nmol/L (95% CI −58.0 to +67.2 with df = 1; corresponding DL normal-CI estimate +4.6, −5.1 to +14.2), I² = 69%, k = 2. Tam 2012 [80] was excluded because the Wan range estimator for that trial is dominated by a single 86.4 nmol/L low-dose outlier and the primary paper reports z = −0.11, P = 0.91 for the between-dose UMFA comparison at Week 12. The diamond width represents the DL normal CI for legibility; the formal HKSJ CI with df = 1 is shown as out-of-range arrows.

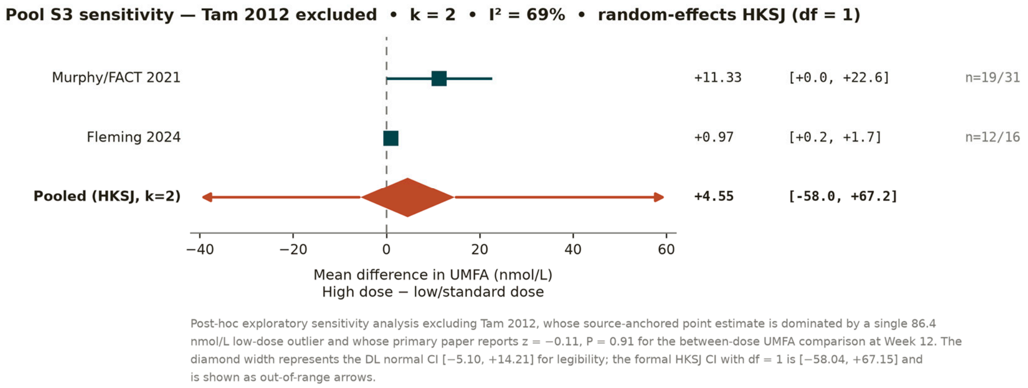

Supplementary Figure S3b — Tam 2012 [80] excluded sensitivity

Supplementary Figure S4 — Pool S4 rebuilt three-genotype panel (*MTHFR* 677C→T × FA on plasma total homocysteine, mean difference µmol/L). Nine study-genotype rows from the three randomized reports that supply the outcome, each trial entered once. **No pooled estimate or per-allele trend is reported (deviation D16);** the pooled values and the −0.46 µmol/L per-allele trend given in the originally submitted version have been withdrawn in full. Miyaki 2005 [90] values are placebo-corrected between-group differences derived from the printed means and SDs; CSPPT/Huang 2018 [120] values are the adjusted between-group differences as

printed; PFAT-Hcy 2024 [83] values are computed from printed arm means and have no derivable interval, so they are drawn as point markers without whiskers. The Miyaki 2005 [90] TT point estimate (−11.71 [−20.39, −3.03]) lies outside the plotted range and is shown as a rust out-of-range arrow; it is not interpretable at face value because the TT placebo arm rose by +4.87 μmol/L with an SD of 16.2 in n = 17. Across the two trials with derivable intervals the genotype gradient is approximately 1.0 to 1.2 μmol/L of additional tHcy reduction per 677T allele. Author-generated using Matplotlib; not adapted from any published figure.

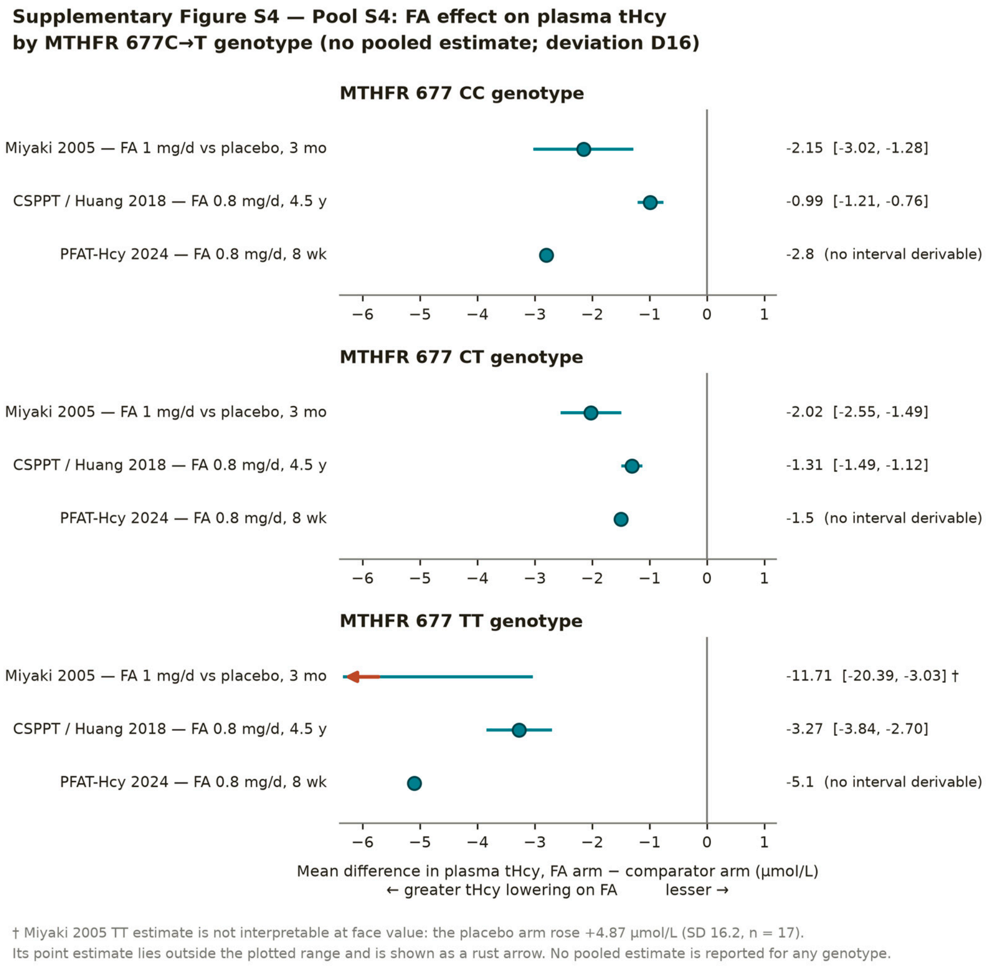

Supplementary Figure S4 — MTHFR 677 × FA on homocysteine

Supplementary Table S10 — Row-level disposition of the originally submitted evidence ledger

Supplementary Table S10: Disposition of every one of the 101 analytic contributions in the evidence ledger as originally submitted, following complete source re-verification (deviation D17). Each row was re-checked against the publication itself. Of the 101 submitted contributions, 21 were confirmed exactly as written, 48 were retained with one or more cells corrected, 1 was reclassified into a different evidence stratum under a new identifier (O14 to I32), and 31 were withdrawn. Two withdrawn mechanistic entries had verified content that belonged in a different stratum: M31 was relocated to a new row O36, and M27 was folded into the existing row O30. The table therefore lists 103 rows — the 101 originally submitted contributions plus the two new identifiers created in revision (O36, I32). The resulting ledger contains 71 analytic contributions (69 retained plus O36 and I32). Original row identifiers are preserved and are not reused, so identifiers absent from

Supplementary Tables S1–S3 are withdrawn entries and can be traced here. The 31 withdrawals fall into four classes: publication predating the 1 January 1995 eligibility window (n = 3: M10, M11, O26); review, meta-analysis, or other secondary synthesis cited as a primary study (n = 8: M15, M21, M22, M23, O21, O23, O25, O32); no publication locatable by author, DOI, title or topic search (n = 8: M14, M16, M25, M28, M30, M33, O18, O33); and a cited publication whose verified content does not support a distinct contribution in that stratum, with the publication's genuine contribution retained elsewhere in the ledger (n = 12: M6, M17, M19, M24, M27, M29, M31, M35, O6, O7, O29, O34).

Original ID	Original design / cohort cell	Original key-finding cell	Disposition and basis
M1	Human ex vivo enzyme kinetics	Hepatic DHFR ~1.4% of rat; 5x variation	**Retained, wording refined.** Source confirmed (PNAS 2009;106:15424). Design and finding correct; sample basis (6 donor livers) now stated.
M2	Human portal vein PK (n=6)	80% of 220 μg FA enters portal vein unmodified	**Retained, wording refined.** Source confirmed (Am J Clin Nutr 2014;100:593). Figure restated as 80 ± 12% of labelled portal folate at 15 min.
M3	Crossover human PK	UMFA at ≥200 μg/dose	**Retained, design corrected.** Kelly 1997 [15] (Am J Clin Nutr 65:1790) reports acute within-subject feeding and single-dose sub-studies; randomization is not stated, so the design is no longer described as a crossover.
M4	Crossover human PK	200 μg/occasion threshold	**Retained, finding corrected and direction reversed.** Sweeney 2007 [16] (BMC Public Health 7:41) detected UMFA at 400 μg/d (2 × 200 μg per occasion) but **not** at 200 or 100 μg/d. The submitted entry stated the threshold at the dose that produced no UMFA.
M5	NHP PET	FA blocks FR*α* tracer uptake at 63–90 ng/mL	**Retained.** Source confirmed (J Cereb Blood Flow Metab 2026;46:976).
M6	Human NK + UMFA (in vitro)	UMFA-FBP reduces NK cytotoxicity	**Withdrawn.** Troen 2006 [23] (J Nutr 136:189) is a cross-sectional study in 105 postmenopausal women and contains no in-vitro folate-binding-protein experiment. The publication's actual observational contribution is retained at O16, so no evidence is lost; only the non-existent mechanistic contribution is removed.
M7	Pre-post NK assay	NK ↓ with UMFA ↑	**Retained, direction clarified.** Paniz 2017 [24] (J Nutr 147:1677) is a single-arm uncontrolled pre-post study in which UMFA **rose** (detectable in 96.6% at day 45) while NK number and cytotoxicity fell.

Original ID	Original design / cohort cell	Original key-finding cell	Disposition and basis
M8	Mouse periconceptional FA	Altered Igf2 methylation	**Retained, finding corrected.** Barua 2014 [27] (PLoS One 9:e101674) reports altered offspring brain gene expression and behaviour. *Igf2* methylation was not an outcome of this study.
M9	Mouse periconceptional FA	Aberrant cardiac dev	**Retained.** Source confirmed (Birth Defects Res A 2013;97:47).
M10	Cell culture	MTX polyglutamates enhance thymidylate synthase inhibition	**Withdrawn.** Allegra 1985 [93] (J Biol Chem 260:9720) predates the 1 January 1995 eligibility window and is cell-free purified-enzyme kinetics rather than cell culture.
M11	Human cancer + marrow cell culture	Leucovorin rescues MTX toxicity	**Withdrawn.** Jolivet 1994 [94] (Biochem Pharmacol 47:659) predates the 1 January 1995 eligibility window.
M12	Population genetics DHFR	19-bp del reduces activity	**Retained, citation disambiguated and finding corrected.** Two Kalmbach 2008 papers exist [45,91]; this row is *J Nutr* 138:2323 [91]. No enzyme activity was measured; the supported finding is a higher prevalence of high plasma UMFA in del/del carriers at folic acid intake ≥500 μg/d (47.0% vs 24.4%; p for interaction = 0.03).
M13	Mouse	NTD transcriptomic changes	**Retained, source resolved and finding corrected.** No Christensen 2015 [148] paper reports neural-tube-defect transcriptomics. The row is resolved to *Am J Clin Nutr* 2015;101:646 (10× folic acid × *Mthfr* genotype), whose finding is MTHFR inhibition, reduced 5-methylTHF and SAM/SAH, and hepatocyte degeneration.
M14	Cell culture	UMFA cytotoxicity supraphysiologic	**Withdrawn — no locatable source.** No cell-culture study by Roth in 2011 on UMFA cytotoxicity could be retrieved by author, DOI or topic search. The only folate-related Roth 2011 publication is a prospective cohort (JAMA 306:1566) with unrelated content.
M15	Cell methylation	DNMT activity with high FA	**Withdrawn.** The closest match, Sauer, Mason & Choi 2009 (Curr Opin Clin Nutr Metab Care 12:30), is a narrative review and is therefore eligible for citation chasing only.

Original ID	Original design / cohort cell	Original key-finding cell	Disposition and basis
M16	Animal ex vivo	DHFR isoform tissue expression	**Withdrawn — no locatable source.** No 2008 Stover primary study on DHFR isoform tissue expression could be retrieved; the nearest 2008 Stover publications are narrative reviews.
M17	Human plasma kinetics	Inter-individual variation	**Withdrawn.** The only Lucock-authored 2003 [30] record (Yates & Lucock, Mol Genet Metab 79:201) is a cross-sectional genotype-by-vitamin-status analysis in 152 patients treated for thromboembolic events; it is not a plasma-kinetics study and did not measure UMFA.
M18	LC-MS/MS validation	UMFA detection method	**Retained.** Source confirmed (Clin Chem 2004;50:423), 42 human sera.
M19	NHANES re-analysis	Assay comparison	**Withdrawn.** Bailey 2020 [48] (Am J Clin Nutr 112:1547) is a cross-sectional NHANES 2011–2014 cognitive analysis, not an assay comparison; that publication's actual contribution is retained at O2.
M20	Affinity-HPLC	UMFA detection; MeFox caveat	**Retained, citation corrected.** No Selhub 2008 affinity-HPLC method paper exists; the affinity-HPLC UMFA data were published as Kalmbach et al., *Am J Clin Nutr* 2008;88:763 [45] (Framingham Offspring, before and after fortification), which is now the cited source.
M21	PCFT cell biology	PCFT prefers reduced folates	**Withdrawn.** Visentin et al. 2014 [7] (Annu Rev Physiol 76:251) is a narrative review.
M22	RFC kinetics	Reduced folates > FA at RFC	**Withdrawn.** Sirotnak & Tolner 1999 [22] (Annu Rev Nutr 19:91) is a narrative review.
M23	FRα transport	FA blocks FRα supraphysiologically	**Withdrawn.** Zhao, Aluri & Goldman 2017 [124] (Mol Aspects Med 53:57) is a narrative review of PCFT and hereditary folate malabsorption.
M24	MTHFR kinetics	UMFA inhibits MTHFR	**Withdrawn.** Clément et al. 2020 [129] (J Gynecol Obstet Hum Reprod 49:101622) is an open clinical treatment study of 5-MTHF in 89 infertile couples with a homocysteine endpoint; it is neither an enzyme-kinetics study nor a study of UMFA.
M25	Mouse Mthfr+/−	Methylation susceptibility	**Withdrawn — no locatable source.** No Hubner 2008 mouse *Mthfr* methylation study could be retrieved; the Hubner 2008–09 record consists of human

Original ID	Original design / cohort cell	Original key-finding cell	Disposition and basis
			colorectal epidemiology and a review.
M26	Mouse FA excess + Mthfr	Embryonic delay/growth retardation	**Retained.** Source confirmed (Birth Defects Res A 2011;91:8).
M27	Cell methylation	UMFA vs MeFox handling	**Withdrawn from S1 and relocated.** Bailey 2010 [46] (Am J Clin Nutr 92:383) is a cross-sectional NHANES 2001–2002 analysis in 1,121 adults, not a cell-methylation study. Its verified content is now carried at O30.
M28	Cell culture	UMFA + B12 interaction	**Withdrawn — no locatable source.** No Vidal 2013 cell-culture study of a UMFA-by-B12 interaction could be retrieved; the Vidal 2013 record is human epigenetic epidemiology.
M29	Mouse fetal growth	Maternal FA dose-response	**Withdrawn.** No Christensen 2018 publication reports a maternal folic-acid dose-response; the closest match (J Nutr 2018;148:501) studies folate **deficiency**. The row is also ambiguous with M13 within a single mouse research programme, so that programme is now represented once, at M13.
M30	NHP gut DHFR	Comparative reductive capacity	**Withdrawn — no locatable source.** No publication matching a Henderson 2017 non-human-primate intestinal DHFR study could be retrieved by author, topic or keyword search, and the entry has no reference-list counterpart.
M31	Human	Repeated post-prandial peaks	**Withdrawn from S1 and relocated.** Sweeney 2009 [96] (BMC Public Health 9:295) is a cross-sectional observational biomarker survey with no post-prandial dosing protocol. Its verified content is now carried at O36.
M32	Human pre-post	SAH elevated in MTHFR TT	**Retained, design and finding corrected.** Zhang 2022 [26] (J Clin Biochem Nutr 71:238) is an 8-arm randomized double-blind controlled trial, not a pre-post study. The TT-specific SAH effect was **not** significant ($p = 0.095$); the significant effects were at 2.4 mg/d overall, in CT ($p = 0.027$) and in CT+TT combined ($p = 0.005$). UMFA was not measured, so the contribution label is reduced to Suggestive.

Original ID	Original design / cohort cell	Original key-finding cell	Disposition and basis
M33	Cohort + mech	UMFA-methylation associations	**Withdrawn — no locatable source.** No Crider 2018 publication combines an NHANES cohort analysis with UMFA-methylation mechanism; the 2018 first-author record is a modelling study of erythrocyte folate and neural-tube-defect risk.
M34	RCT metabolomics	UMFA-arachidonate links	**Retained.** Source confirmed (J Nutr 2024;154:670).
M35	Cohort + cell methylation	UMFA-LINE-1 methylation	**Withdrawn.** Pauwels 2017 [150] measured *IGF2* DMR, *DNMT1*, *LEP* and *RXRA* methylation; LINE-1 and UMFA were not measured, and the study is human observational rather than mechanistic. Its verified content is carried at O19.
O1	NHANES 2007-10	25–40% fasting UMFA detectable	**Retained, both cells corrected.** Pfeiffer 2015 [18] analyses NHANES **2007–2008**. UMFA was detectable in >95% of samples; the 33.2% and 21.0% figures refer to UMFA >1 nmol/L overall and in fasting adults ≥20 y. The submitted entry inverted a near-universal detection rate into a minority prevalence.
O2	NHANES 2011-14	UMFA × low B12 cognition OR 2.16	**Retained.** Source confirmed (Am J Clin Nutr 2020;112:1547).
O3	NHANES 1999-02	Folate-B12 cognition interaction	**Retained, relevance caveat added.** Serum folate, not UMFA, was measured.
O4	NHANES	UMFA-cognitive impairment	**Retained, survey years specified** (NHANES 1999–2002, n = 1,858).
O5	Toronto pregnancy	Pregnancy UMFA prevalence	**Retained, quantified** (97% maternal and 93% cord detection).
O6	UK pregnancy (cohort arm)	400 µg/d safe for cord UMFA	**Withdrawn from S2.** Pentieva 2016 [97] is a randomized placebo-controlled trial; the parenthetical “cohort arm” label does not make it observational. The publication is already carried as trial I7, so this row was a duplicate contribution in the wrong stratum.
O7	US pregnancy	800 µg ↑ maternal UMFA	**Withdrawn from S2.** Fleming 2024 [86] is a double-blind randomized dose-comparison trial, already carried as I8.

Original ID	Original design / cohort cell	Original key-finding cell	Disposition and basis
O8	Boston Birth Cohort	High maternal plasma folate/B12 and ASD risk	**Retained.** Source confirmed (Paediatr Perinat Epidemiol 2018;32:100). Cohort overlap with O9 is now stated.
O9	BBC update	Cord UMFA Q4 aOR 2.26	**Retained.** Source confirmed (Am J Clin Nutr 2020;112:1304).
O10	MARBLES	First-month prenatal vitamin use and lower ASD recurrence	**Retained, relevance caveat added.** Exposure was supplement use; UMFA was not measured.
O11	MoBa, ASM-exposed pregnancies	Maternal plasma UMFA and offspring autistic traits	**Retained, direction corrected.** Husebye 2022 [44] is a **null** study. The contribution label is changed from Supporting to Neutral.
O12	MoBa, AED-exposed pregnancies	Periconceptional FA and autistic traits	**Retained, relevance caveat added.** Plasma folate, not UMFA, was measured.
O13	FOCUS CRC	HR 1.31 per twofold increase	**Retained.** Source confirmed (JNCI Cancer Spectr 2020;4:pkaa051); the estimate applies within the folic-acid-detectable subgroup (n = 296).
O14	AFPPS post-hoc	FA HR 2.63 prostate Ca	**Reclassified to S3 as I32.** Figueiredo 2009 [57] is the placebo-controlled Aspirin/Folate Polyp Prevention Study, a randomized trial, and is contained within the Vollset et al. pooled analysis [109].
O15	EPIC breast cancer	UMFA × alcohol	**Retained, finding corrected.** Matejcic 2017 [100] measured plasma folate and B12 but **not** UMFA; the reported alcohol interaction is for B12. No UMFA-by-alcohol result exists in this publication.
O16	Framingham OOA	UMFA ↓ NK cytotoxicity	**Retained, cohort corrected.** Troen 2006 [23] studied 105 healthy postmenopausal women, not the Framingham or Old Order Amish cohorts.
O17	NHANES	FA & immune markers	**Retained, both cells corrected.** Hirsch 2009 [65] (Eur J Gastroenterol Hepatol 21:436) is an ecological before-and-after analysis of Chilean national hospital-discharge rates with a colon-cancer outcome; it is not an NHANES analysis and reports no immune marker.
O18	NHANES	UMFA &	**Withdrawn — no locatable source.** No Crider 2018

Original ID	Original design / cohort cell	Original key-finding cell	Disposition and basis
		methylation	NHANES analysis of UMFA and methylation exists; the searched 2018 output covers antibiotics and birth defects, corn masa flour fortification, and erythrocyte-folate modelling.
O19	MAVIDOS	UMFA-LINE-1 methylation	**Retained, cohort and finding corrected.** The cohort is MANOE (Belgium), not MAVIDOS; the methylation targets were *IGF2* DMR, *DNMT1*, *LEP* and *RXRA*, and neither LINE-1 nor UMFA was measured.
O20	ASD case series	FRAA 60–75% prevalence	**Retained, figure corrected.** Frye 2013 [34] reports a single prevalence of 75.3%, not a 60–75% range.
O21	NTD case-control	FRAA in NTD mothers	**Withdrawn.** Sequeira et al. 2013 [111] (Clin Chem Lab Med 51:545) is indexed as a review and contains no original case-control dataset.
O22	CFD cohort + cases	FRAA-CFD-CSF 5-MTHF	**Retained, citation and population corrected.** The source is Ramaekers et al., *Neuropediatrics* 2007;38:276, a case series of 25 children with early-onset low-functioning autism; it is added to the reference list, which previously contained no 2007 Ramaekers entry.
O23	ASD case-control	FRAA-autism phenotype	**Withdrawn.** The row resolves to Frye & Rossignol 2014 [112] (Front Pediatr 2:66), a narrative review with no original cohort.
O24	Multi-site	FRAA standardization	**Retained, both cells corrected.** Quadros 2018 [131] is a single-region family-based prevalence study in Brooklyn and Staten Island, not a multi-site assay-standardization study. FRAA positivity was 76% in ASD children versus 29% in controls.
O25	Cohort	FRAA + dietary triggers	**Withdrawn.** The nearest match, Bobrowski-Khoury et al. 2021 [125] (J Pers Med 11:710), is a narrative review with no human cohort and no dietary-trigger dataset.
O26	Pregnancy	Folate forms (pre-fortification)	**Withdrawn — pre-1995 and no locatable source.** The stated year (1994) falls outside the eligibility window, and no folate-related Lubec 1994 publication could be retrieved.
O27	Older adults	UMFA accumulation	**Retained, population and finding corrected.** Obeid

Original ID	Original design / cohort cell	Original key-finding cell	Disposition and basis
		with age	2010 [151] (Am J Clin Nutr 92:1416) studied 87 pregnant women at delivery and 29 cord samples, not older adults, and reports no age-related accumulation. Folic acid >0.20 nmol/L was found in 44% of women and 55% of cord samples.
O28	NHANES MeFox	MeFox vs UMFA distinction	**Retained, authorship and design corrected.** Reference [72] is Fazili et al., *J Nutr* 2014;144:1873 (not Pfeiffer), a laboratory specimen-handling and stability experiment rather than an NHANES population survey. No 2014 NHANES MeFox analysis exists.
O29	Pregnancy forms	UMFA-5-MTHF correlations	**Withdrawn.** No Plumptre 2018 folate publication exists. The only candidate (Barzilay et al., Nutr Res 2018;55:57) is a gestational-diabetes comparison in the same Toronto cohort as O5 and does not report UMFA.
O30	NHANES	High serum folate prevalence	**Retained, citation corrected.** No Bailey 2018 NHANES folate paper exists. The row is resolved to Bailey et al., *Am J Clin Nutr* 2010;92:383 [46] (NHANES 2001–2002, 1,121 adults ≥60, UMFA detected in 38%), which also absorbs the verified content of the withdrawn M27.
O31	CRC cohort	Folate timing hypothesis	**Retained, design corrected.** Mason 2007 [66] (Cancer Epidemiol Biomarkers Prev 16:1325) is an ecological time-series hypothesis paper with no enrolled participants; ROBINS-I is set to Serious accordingly.
O32	Meta-cohort	FA & cancer outcomes	**Withdrawn.** Vollset 2013 [109] is a meta-analysis of 13 randomized folic acid trials (n = 49,621), not an observational cohort, and its overall result is **null** (rate ratio 1.06, 0.99–1.13, p = 0.10) rather than supporting. As a pooled secondary synthesis it is ineligible as included evidence under Section 2.2, so it is withdrawn from the ledger rather than reclassified, and is retained only as a contextual citation [109] where the randomized cancer-safety evidence is discussed (deviation D19).
O33	EarlyBird	FA exposure	**Withdrawn — no locatable source.** No publication

Original ID	Original design / cohort cell	Original key-finding cell	Disposition and basis
		cognitive trajectory	links the EarlyBird cohort to folate or UMFA, and no Wang 2019 folate-cognition study in that cohort could be retrieved.
O34	Aus pregnancy	UMFA & multivitamins	**Withdrawn from S2.** Sulistyoningrum 2024 [50] is a double-blind randomized trial, already carried as I9.
O35	Single-center cohort	Maternal FRAA × NT ≥3.5 mm → ASD	**Retained, denominators stated.** 3,600 scans yielded 27 with nuchal translucency ≥3.5 mm, of whom 11 genetically negative pregnancies were followed; the analytic n of 11 is now explicit.
O36	(new)	–	**New row.** Carries the verified content of the withdrawn M31 (Sweeney 2009 [96], BMC Public Health 9:295) in the correct stratum: a cross-sectional biomarker survey of 50 blood donors and 20 fasted mother–cord pairs.
I1	FA dose-escalation crossover	UMFA at ≥200 µg	**Retained, design corrected.** Randomization is not stated in Kelly 1997 [15], so the entry no longer describes a crossover; OCEBM level lowered from 1b to 2b.
I2	FA fortification crossover	200 µg/occasion threshold	**Retained, design corrected.** Sweeney 2007 [16] is a non-randomized fixed-order dose-descending study with no comparison arm; OCEBM level lowered from 1b to 2b.
I3	FA vs 5-formylTHF crossover	Portal UMFA 80%	**Retained.** Source confirmed.
I4	5 mg/d FA × 90 d single arm	UMFA & NK ↓	**Retained, direction corrected.** UMFA **increased** while NK number and cytotoxicity fell.
I5	1 vs 5 mg FA	UMFA at 6 wk	**Retained, finding completed.** Detectable UMFA rose in **both** arms with no between-dose difference at any time point; tHcy was not measured.
I6	1 vs 4–5 mg FA pregnancy	Maternal UMFA	**Retained, citation and status corrected.** This is an ancillary biomarker analysis of the Folic Acid Clinical Trial, not a primary trial report. Reference [43] carried the wrong authors, title, DOI and pagination and has been rebuilt (Murphy et al., Am J Clin Nutr 2021;113:1361, doi 10.1093/ajcn/nqaa407).

Original ID	Original design / cohort cell	Original key-finding cell	Disposition and basis
I7	400 µg vs placebo pregnancy	UMFA maternal/cord	**Retained.** Source confirmed.
I8	400 vs 800 µg FA pregnancy	UMFA dose-response	**Retained.** Source confirmed; tHcy was not measured.
I9	Multivit ± FA after 12 wk gest	UMFA	**Retained.** Source confirmed.
I10	Cochrane 2023 [81]	UMFA + RBC folate	**Retained, year corrected to 2024** (Br J Nutr 131:92; e-published 2023). Shared parent trial with I14 is now stated on both rows.
I11	FA vs L-5-MTHF infant formula	UMFA + growth	**Retained.** Source confirmed; outcome-specific RoB 2 judgement unchanged.
I12	400 vs 800 µg/d mixture	Serum folate forms	**Retained, risk of bias raised.** The trial is open-label, so the submitted "Low" judgement is replaced by Some concerns.
I13	FA vs (6S)-5-MTHF pregnancy 24 wk	UMFA + placenta	**Retained.** Source confirmed; RoB 2 High retained.
I14	Cochrane 2024 (human milk) [83]	Human milk UMFA	**Retained, year corrected to 2023** (Sci Rep 13:11298). The submitted ledger had the I10 and I14 years transposed.
I15	FA in older adults RCT	UMFA accumulation	**Retained, intervention corrected.** The active arm was folic acid 5 mg **plus** vitamin B6 40 mg and vitamin B12 2 mg, so the observed effect cannot be attributed to folic acid alone.
I16	FA vs B-complex RCT	UMFA prevalence	**Retained, risk of bias raised.** Single-blind with no placebo control; Low is replaced by Some concerns.
I17	FA × MTHFR genotype	Hcy response	**Retained.** Source confirmed (J Hum Genet 2005;50:241); genotype-stratified randomized allocation verified against Figure 1 and Table 2.
I18	FA + B-vit CV outcomes	CV events	**Retained.** Source confirmed.
I19	FA + B-vit post-stroke	Stroke	**Retained.** Source confirmed.
I20	FA + B-vit post-stroke	Stroke	**Retained, comparator clarified.** The control arm received a low-dose vitamin formulation, not

Original ID	Original design / cohort cell	Original key-finding cell	Disposition and basis
			placebo.
I21	800 µg FA × 3 y cognition	Cognition	**Retained, endpoint status clarified.** Cognition was a pre-specified secondary endpoint.
I22	B-PROOF / de Jager 2012 [119]	Cognition	**Retained, trial identity corrected.** The publication reports **VITACOG**, not B-PROOF, which is a separate Dutch trial. Vitamin B6 20 mg, omitted from the submitted entry, is part of the regimen.
I23	FA + enalapril × MTHFR	Stroke	**Retained.** Source confirmed.
I24	FA dose × MTHFR CT/TT	SAH/SAM	**Retained, outcome corrected.** The primary outcome is change in plasma SAH only; no S-adenosylmethionine treatment effect is reported, and tHcy appears only as a baseline characteristic and covariate.
I25	Folinic acid vs placebo ASD	Verbal communication	**Retained.** Source confirmed.
I26	Folinic acid adjunct ASD	Inappropriate speech	**Retained.** Source confirmed; per-arm n is not reported in the source.
I27	Folinic acid vs placebo ASD	ADOS global (RCT)	**Retained, inference downgraded.** The significant ADOS result is a within-arm pre-post change; no between-group p-value is reported. Risk of bias raised to Some concerns and the contribution label reduced to Suggestive.
I28	Folinic acid + rehabilitation	PEP-3 social reciprocity	**Retained, risk of bias raised.** The trial is open-label with no placebo and unequal allocation; Low is replaced by Some concerns. The genotype analysis was conducted within the intervention arm only.
I29	Folinic acid 7.5 mg/d preconception	Offspring neurotypical at 3 y	**Retained, dose withdrawn.** The stated dose of 7.5 mg/d could not be verified; the publisher full text was inaccessible and no bibliographic record states a dose. The dose is now reported as not stated.
I30	Leucovorin 15 mg BID vs FA 0.4 mg/d	Offspring ASD at 24–30 mo	**Retained, dose corrected 30-fold and risk of bias raised.** Trial registry ISRCTN96269039 specifies leucovorin **0.5 mg twice daily**, not 15 mg twice daily. Attrition from 29 randomized to 18 completed raises

Original ID	Original design / cohort cell	Original key-finding cell	Disposition and basis
			the RoB 2 judgement from Low to Some concerns.
I31	FA metabolomics substudy	Metabolomic profile	**Retained, trial identity disambiguated.** This "FACT" is the Folic Acid and Creatine Trial in Bangladeshi adults, a different trial from the Folic Acid Clinical Trial underlying I6.
I32	(new)	–	**New row.** Carries the reclassified O14 (Figueiredo 2009 [57], Aspirin/Folate Polyp Prevention Study) in the correct stratum, flagged as contained within the Vollset et al. pooled analysis [109].

Note. The eight withdrawals in the "no publication locatable" class are reported as unresolved rather than replaced. Where a plausible alternative publication exists, it is named in this table for transparency, but no substitute source was adopted to preserve a submitted claim. Any of these entries can be reinstated if the underlying reference is supplied.
